# Prodrugs of γ‐Alkyl‐Modified Nucleoside Triphosphates: Improved Inhibition of HIV Reverse Transcriptase

**DOI:** 10.1002/anie.202003073

**Published:** 2020-09-30

**Authors:** Chenglong Zhao, Stefan Weber, Dominique Schols, Jan Balzarini, Chris Meier

**Affiliations:** ^1^ Organic Chemistry Department of Chemistry University of Hamburg Martin-Luther-King-Platz 6 20146 Hamburg Germany; ^2^ Laboratory of Virology and Chemotherapy Department of Microbiology and Immunology Rega Institute for Medical Research KU Leuven Herestraat 49 3000 Leuven Belgium

**Keywords:** antiviral agents, biological activity, DNA polymerases, nucleoside triphosphates, prodrugs

## Abstract

The development of nucleoside triphosphate prodrugs is one option to apply nucleoside reverse transcriptase inhibitors. Herein, we report the synthesis and evaluation of d4TTP analogues, in which the γ‐phosphate was modified covalently by lipophilic alkyl residues, and acyloxybenzyl prodrugs of these γ‐alkyl‐modified d4TTPs, with the aim of delivering of γ‐alkyl‐d4TTP into cells. Selective formation of γ‐alkyl‐d4TTP was proven with esterase and in CD4^+^‐cell extracts. In contrast to d4TTP, γ‐alkyl‐d4TTPs proved highly stable against dephosphorylation. Primer extension assays with HIV reverse transcriptase (RT) and DNA‐polymerases α, β or γ showed that γ‐alkyl‐d4TTPs were substrates for HIV‐RT only. In antiviral assays, compounds were highly potent inhibitors of HIV‐1 and HIV‐2 also in thymidine‐kinase‐deficient T‐cell cultures (CEM/TK^−^). Thus, the intracellular delivery of such γ‐alkyl‐nucleoside triphosphates may potentially lead to nucleoside triphosphates with a higher selectivity towards the viral polymerase that can act in virus‐infected cells.

## Graphical abstract

**γ‐Alkyl‐nucleoside triphosphates** were studied as DNA‐polymerase inhibitors. A strong selectivity for HIV reverse transcriptase over DNA polymerases α, β, and γ was observed. γ‐Alkyl‐nucleoside triphosphates proved highly stable in cell extracts in contrast to the parent NTPs. Lipophilic prodrugs were also prepared. High anti‐HIV activity was detected in infected CEM/TK‐deficient cells, making these compounds promising potential antivirals.

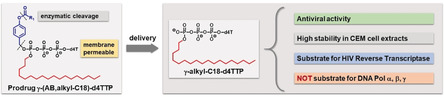

## Introduction

**Scheme 1:**
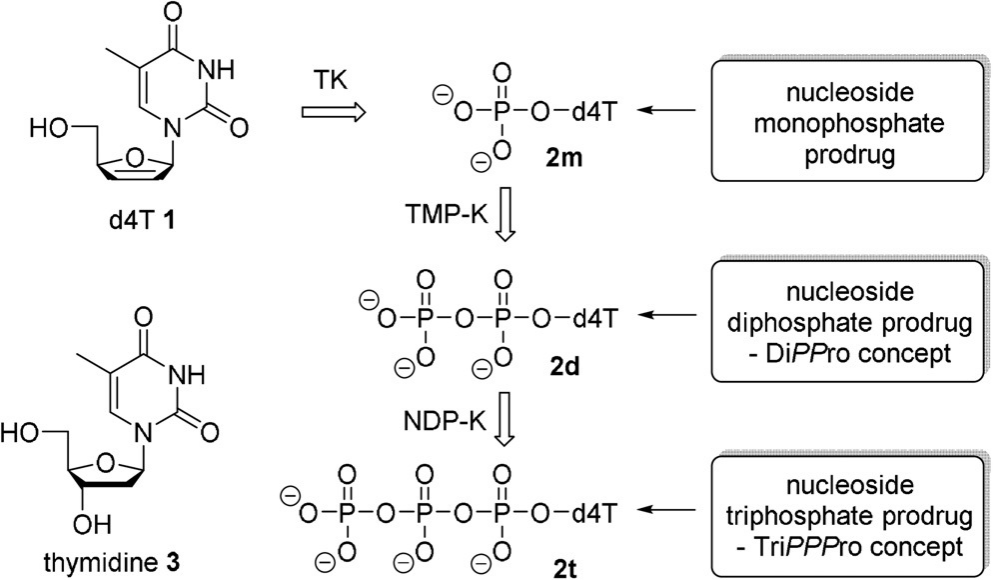
Metabolism of nucleoside analogues such as d4T **1** and the corresponding nucleotide prodrugs thereof.

Over the last decades a variety of nucleoside analogues have been applied in antitumor and antiviral therapy and still play an important role in combatting HIV, herpes viruses, and hepatitis B and hepatitis C viral infections.[^1^, ^2^] The targets of these nucleoside analogue drugs are the virus‐encoded DNA‐ or RNA polymerases, such as HIV reverse transcriptase (RT)[^3^, ^4^] or the hepatitis C‐encoded RNA‐dependent RNA‐polymerase NS5B.^[5]^ To date, several nucleoside analogues have been approved as HIV reverse transcriptase inhibitors (NRTIs)^[6]^ and they are used as the backbone of the combined antiretroviral therapy (cART). However, the antiviral activity of nucleoside analogues, such as 3′‐deoxy‐2′,3′‐didehydrothymidine **1** (d4T), a thymidine (**3**) analogue, is dependent on the intracellular phosphorylation by host cell kinases into their nucleoside triphosphates (NTPs), for example, d4TTP **2 t** via the nucleoside mono‐ (**2 m**, here: d4TMP) and the diphosphate (**2 d**, here: d4TDP).[^7^, ^8^] This stepwise transformation into the corresponding triphosphates, such as **2 t**, often occurs insufficiently by the involved kinases due to structural differences to the natural nucleosides (for example, TMP **4 m**, TDP **4 d**, and TTP **4 t**). Furthermore, limitations, such as poor biological half‐lives due to catabolic elimination, variable bioavailability after oral administration or selection of drug resistance, counteract the use of nucleoside analogues.^[9]^ To overcome some of these hurdles, prodrugs of the first phosphorylated metabolite (nucleoside monophosphate, NMP; Scheme 1) have been explored in the past.[^10^, ^11^, ^12^, ^13^, ^14^, ^15^, ^16^] This resulted in orally applicable forms of a few antiviral NMPs that are used as powerful drugs. Recently, we have reported a successful NDP‐delivery approach using lipophilic, but still partially charged, NDP prodrugs (Di*PP*ro approach; Scheme 1).[^17^, ^18^, ^19^]

For the delivery of the ultimately active nucleoside analogue triphosphate several approaches using nanoparticles or nanogels (nanocarriers) were tried.^[20]^ This was mainly done because it was thought that it is impossible to design chemical NTP prodrugs.^[21]^ However, we recently reported the first chemical delivery system for NTPs through a prodrug technology (Tri*PPP*ro approach **5**; Scheme 1).[^22^, ^23^, ^24^, ^25^] We proved for d4T **1** and other nucleoside analogues that the corresponding Tri*PPP*ro compounds even retained pronounced anti‐HIV activity in thymidine‐kinase‐deficient T‐cell cultures (CEM/TK^−^), whereas the parent d4T **1** was virtually inactive in these cells due to the lack of phosphorylation. The membrane permeability was achieved by two covalently but bioreversibly attached 4‐acceptor‐substituted benzyl esters at the γ‐phosphate group. The enzyme‐driven cleavage of both masks from Tri*PPP*ro compounds **5** by an initial cleavage of the acyloxy ester moiety and a subsequent spontaneous cleavage of the remaining part of the mask led to the formation of d4TTP **2 t** (Supporting Information, Scheme SI1). The cellular uptake of these compounds was proven by a using fluorescent nucleoside analogue.^[24]^

The development of NTP prodrugs is highly desirable because this would lead to the bypass of all steps of phosphorylation and would in principle maximize the intracellular concentration of the ultimately bioactive NTP.^[26]^ However, there are two important unsolved issues. The first drawback can be related to the intracellular metabolic instability of NTPs due to fast enzymatic dephosphorylation. Assuming an efficient delivery of NTPs from the prodrug form, a rapid enzymatic dephosphorylation would counteract the original idea of producing high intracellular concentrations of the NTPs because rephosphorylation is often inefficient in the case of nucleoside analogues.[^7^, ^8^] Thus, a metabolic stabilization of the triphosphate unit is needed in order to keep the concentration as high as possible. Secondly, an as high as possible selectivity of the nucleoside analogue triphosphate for viral polymerases and not for cellular polymerases, such as DNA polymerases α, β, and γ, should be achieved. It was observed previously that pyrimidine nucleoside triphosphates, including d4TTP inhibited DNA polymerase β and even more DNA polymerase γ, while not being a substrate for DNA polymerase α.^[27]^ Particularly, the inhibition of the mitochondrial DNA polymerase γ is crucial and often associated with marked toxicity effects.[^28^, ^29^]

**Figure 1:**
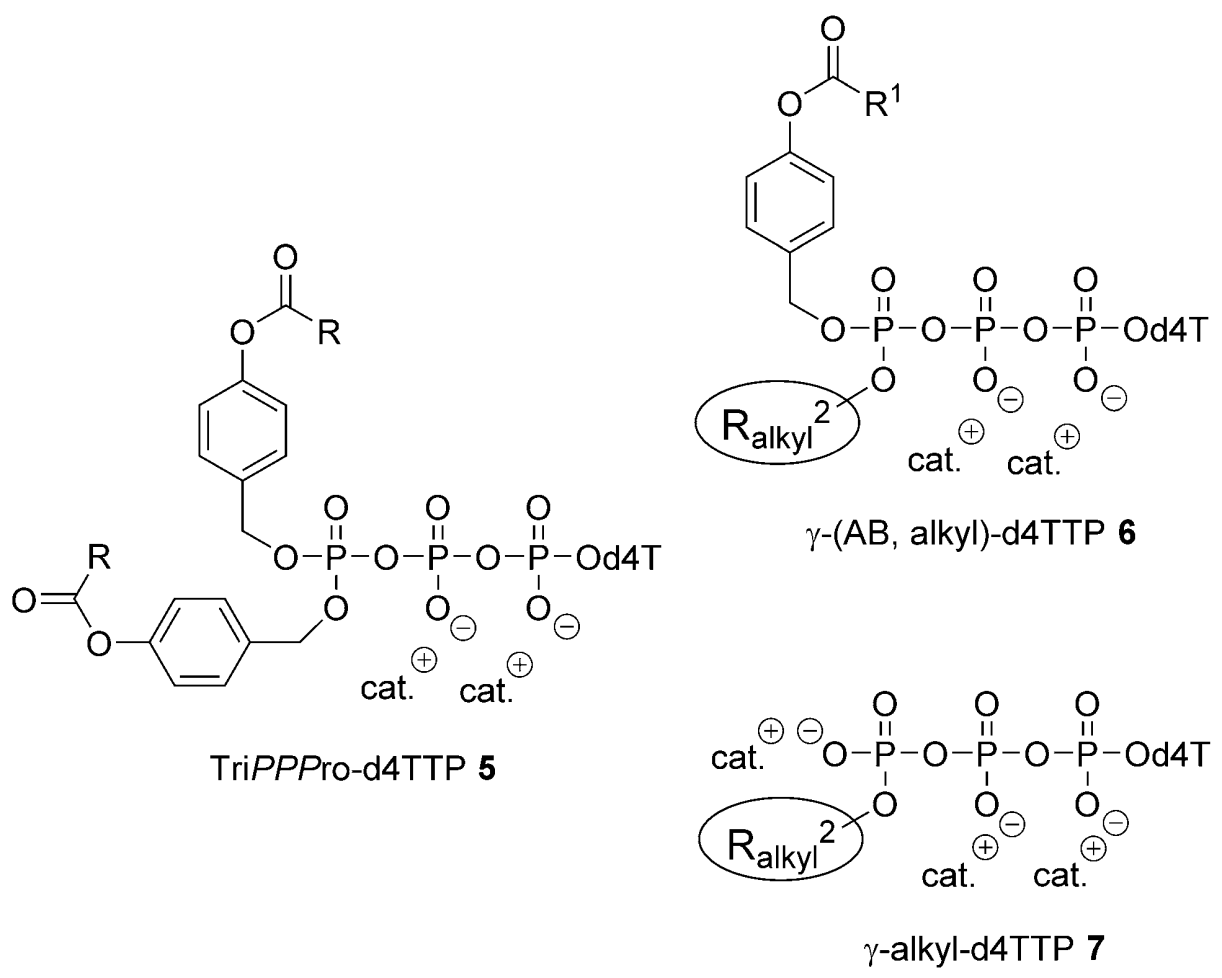
Acyloxybenzyl prodrugs of γ‐alkyl‐d4TTPs **6** and the demasked γ‐alkyl‐d4TTP derivatives **7**.

Although the described compounds were not fully characterized, Krayevsky et al. reported that the replacement of the γ‐phosphate group by a methyl‐ or a phenylphosphonate moiety had almost no effect on the substrate properties towards retroviral RT, but abolished their ability to function towards DNA polymerases α and β.[^30^, ^31^] Moreover, the authors reported that such compounds led to an increase in stability of the triphosphate unit in human serum (about 10‐fold).^[32]^ However, their compounds proved to be completely inactive in antiviral tests and no prodrug forms were studied. Thus, we decided to explore lipophilic prodrugs of γ‐alkyl‐NTPs. A further motivation that such an approach is worth exploring was obtained from our own previous work. In contrast to Tri*PPP*ro compounds **5** (unpublished results and refs. [33] and [34]), the intermediate that comprised only one masking group (Supporting Information, Scheme SI1) proved to be a substrate for HIV‐RT and d4TMP was incorporated by HIV‐RT in a primer extension assay into the DNA strand. Although to a lesser extent than the Tri*PPP*ro derivatives **5**, the monomasked d4TTP derivative comprising a lipophilic octadecyloxybenzyl moiety surprisingly proved to be anti‐HIV active in the cell assay using CEM/TK^−^ cells (EC_50_=1.5 μm).^[34]^ These observations guided us to design NTP prodrugs **6** that comprise different length γ‐alkyl chains in combination with a biodegradable acyloxybenzyl group. The aim was the intracellular delivery of γ‐alkyl‐NTPs **7** (Figure 1).

## Results and Discussion

### Synthesis of γ‐(AB,Alkyl)‐d4TTP Prodrugs 6

**Scheme 2:**
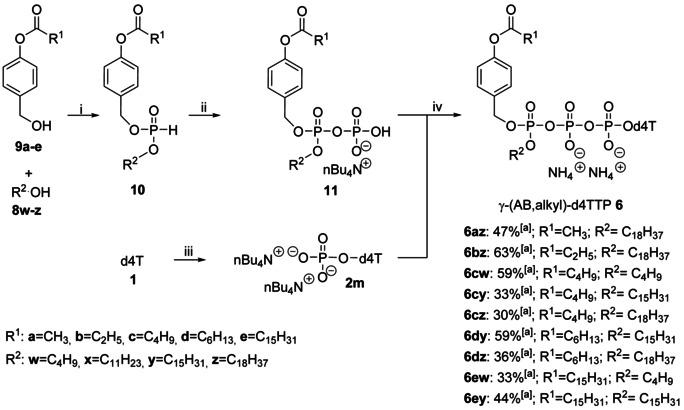
Reagents and conditions: i) DPP, pyridine, 0 °C–38 °C, 3.3 h; ii) a) NCS, CH_3_CN, RT, 2 h, b) (H_2_PO_4_)Bu_4_N, CH_3_CN, RT, 1 h; iii) d4T **1**, POCl_3_, pyridine, H_2_O, CH_3_CN, 0 °C–RT, 5 h; iv) a) TFAA, Et_3_N, CH_3_CN, 0 °C, 10 min, b) 1‐methylimidazole, Et_3_N, CH_3_CN, 0 °C–RT, 10 min, c) d4TMP **2 m**, RT, 3–5 h, rp‐chromatography, Dowex 50WX8 (NH_4_^+^ form) ion exchange, rp‐chromatography. [a] Yields are given for the conversion of **2 m** to **6**.

Title compounds **6** were synthesized using the *H*‐phosphonate route (Scheme 2).^[24]^ First, d4TMP **2 m** was prepared from d4T **1** as described before.^[35]^ The d4TMP 2×NH_4_^+^ salt was isolated and converted into its acid form by a Dowex 50WX8 (H^+^) ion exchange and then titrated with tetra‐*n*‐butylammonium hydroxide solution to pH 7 followed by freeze drying.

The highly hygroscopic di(*n*Bu_4_N)^+^ salt of d4TMP **2 m** was rigorously dried before use. Next, diphenyl *H*‐phosphonate (DPP) was reacted with alcohols **8** and 4‐acyloxybenzyl alcohols **9** to form mixed *H*‐phosphonates **10**.^[36]^

This *H*‐phosphonate route offered two main advantages as compared to the phosphoramidite method: i) *H*‐phosphonates **10** were found to be stable at −20 °C for more than two years and ii) d4TMP was easier to synthesize and additionally more stable than the corresponding d4TDP. Next, compounds **10** were reacted with *N*‐chlorosuccinimide (NCS). The formed corresponding phosphorochloridates were further reacted with tetra‐*n*‐butylammonium phosphate to give pyrophosphates **11** in almost quantitative yields. Due to their chemical lability, pyrophosphates **11** were quickly purified by extraction and immediately used in the next step. The final reaction was started with an activation of pyrophosphates **11** (1.3 equiv) with trifluoroacetic acid anhydride (TFAA) followed by *N*‐methylimidazole and then coupled with d4TMP **2 m** (1 equiv) to form γ‐(AB,alkyl)‐NTPs **6** (*n*‐Bu_4_N^+^ form). After a reversed‐phase (rp) column chromatography step and a Dowex 50WX8 (NH_4_^+^) ion exchange step, followed by a second rp‐column chromatography step, γ‐alkyl‐NTP prodrugs (NH_4_^+^ form) **6** were isolated as diastereomeric mixtures reliably in 33–63 % yield (Scheme 2). These diastereomeric mixtures were inseparable by chromatography and indistinguishable in the ^31^P‐NMR spectra.

### Synthesis of γ‐Alkyl‐d4TTPs 7 and γ‐Alkyl‐TTP 15

**Scheme 3:**
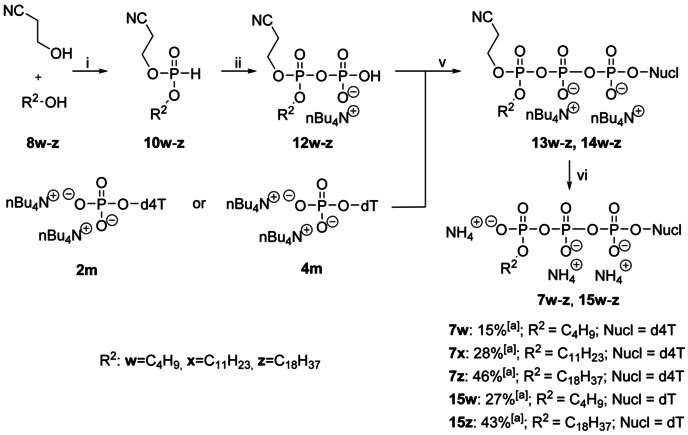
Reagents and conditions: i) DPP, pyridine, 0 °C–38 °C, 3.3 h; ii) a) NCS, CH_3_CN, RT, 2 h, b) (H_2_PO_4_)Bu_4_N, CH_3_CN, rt, 1 h; iii) a) TFAA, Et_3_N, CH_3_CN, 0 °C, 10 min, b) 1‐methylimidazole, Et_3_N, CH_3_CN, 0 °C–RT, 10 min, c) NMP, RT, 3–5 h, rp chromatography, d) *n*‐Bu_4_N^+^OH^−^, Dowex 50WX8 (NH_4_^+^ form) ion exchange, rp‐column chromatography. [a] yields are calculated for the conversion from **2 m**, **4 m** to **7**, **15**.

To synthesize γ‐alkyl‐d4TTPs **7**, the β‐cyanoethyl group was used during the synthesis as protection group for the γ‐phosphate group. After the coupling reaction of compounds **12 w**–**z** and d4TMP **2 m** to give the γ‐protected nucleoside triphosphates **13 w**–**z**, the crude reaction product was stirred in a mixture of 40 % nBu_4_N^+^OH^−^ (water solution) and acetonitrile at room temperature to yield target compounds **7 w**–**z**. The reaction was monitored by HPLC and stopped after 8–20 hours. γ‐Alkyl‐d4TTPs **7 w**–**z** were isolated in 15–46 % yield (Scheme 3). By using the same method which led to γ‐alkyl‐d4TTP **7**, γ‐alkyl‐TTPs **15** were synthesized as well for the primer extension experiment.^[37]^

### Stability Studies

**Scheme 4:**
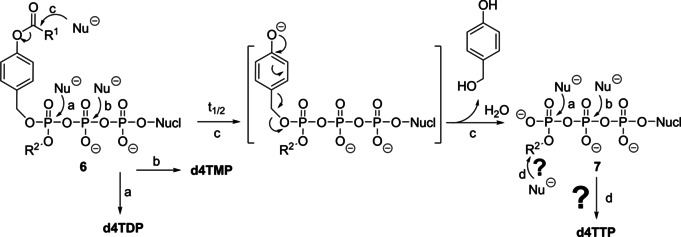
Hydrolysis and delivery mechanism of γ‐(AB,alkyl)‐NTP prodrugs **6**.

γ‐(AB,alkyl)‐NTP prodrugs **6** and γ‐alkyl‐NTPs **7** were incubated in aqueous phosphate buffer (PBS, 25 mm, pH 7.3), or were exposed to pig liver esterase (PLE) in phosphate buffer (PBS) and to human CD4^+^ T‐lymphocyte cell extracts to study their stability and the product distribution. The hydrolysis mixtures were analyzed by means of analytical rp‐18‐HPLC. The calculated half‐lives of prodrugs **6** (Table 1, *t*_1/2_) reflect the removal of the bioreversible AB group to yield γ‐alkyl‐NTPs **7**. Possible hydrolysis pathways and products are summarized in Scheme 4.

**Table 1:** Hydrolysis half‐lives of γ‐alkyl‐d4TTP prodrugs **6**, γ‐alkyl‐NTPs **7**,**15** and Tri*PPP*ro compounds **5** in different media and their antiviral activity in CEM‐cells. 
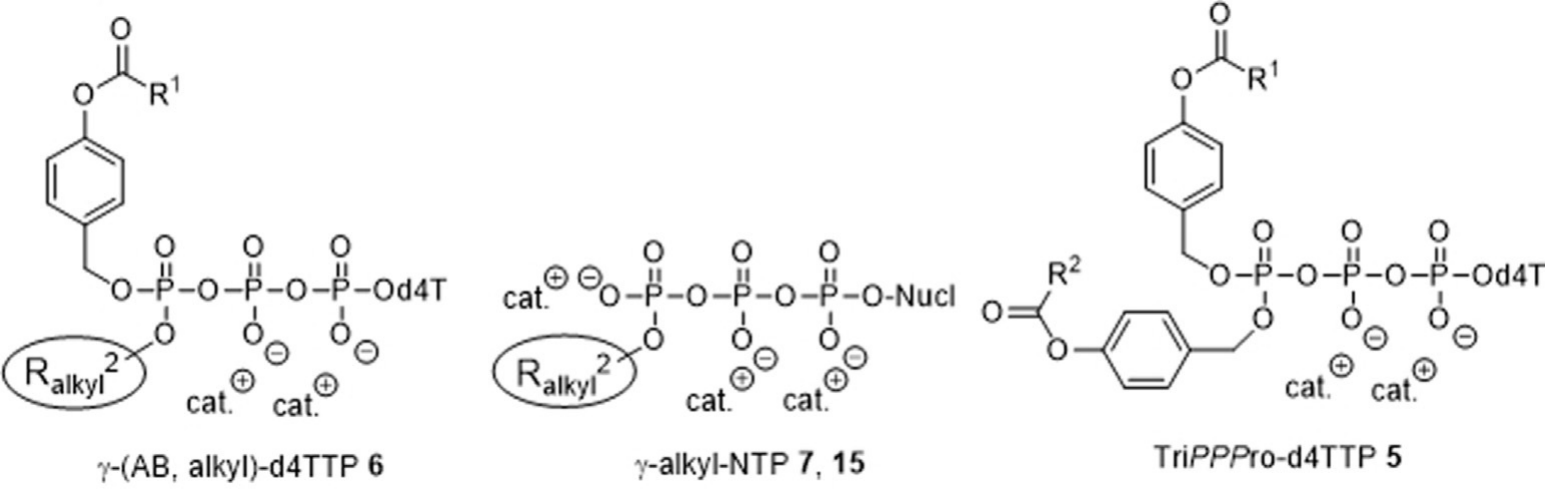

Compound	Nucleos(t)ide	R^1^	R^2^	PBS pH 7.3	CEM/0 cell extracts	EC_50_ [μm]^[a]^		EC_50_ [μm]^[a]^	CC_50_ [μm]^[b]^
								CEM/TK^−^	CEM/0
						CEM/0			
				*t*_1/2_ [h]	*t*_1/2_ [h]	HIV‐1	HIV‐2	HIV‐2	
**6 az**	d4T	CH_3_	C_18_H_37_	196	0.4	0.22±0.05	0.50±0.14	0.39±0.30	17±5
**6 bz**	d4T	C_2_H_5_	C_18_H_37_	206	1.6	0.19±0.09	0.25±0.00	0.35±0.25	11±4
**6 cw**	d4T	C_4_H_9_	C_4_H_9_	94	0.7	0.50±0.21	0.67±0.88	9.74±3.44	64±11
**6 cy**	d4T	C_4_H_9_	C_15_H_31_	197	3.4	0.25±0.00	0.23±0.18	0.39±0.30	21±7
**6 cz**	d4T	C_4_H_9_	C_18_H_37_	237	4.8	0.18±0.09	0.16±0.10	0.17±0.00	13±1
**6 dy**	d4T	C_6_H_13_	C_15_H_31_	212	3.6	0.18±0.01	0.16±0.11	0.92±0.12	18±4
**6 dz**	d4T	C_6_H_13_	C_18_H_37_	269	5.2	0.17±0.00	0.21±0.06	0.76±0.11	27±4
**6 ew**	d4T	C_15_H_31_	C_4_H_9_	198	2.2	0.33±0.11	0.24±0.01	>10	21±2
**6 ey**	d4T	C_15_H_31_	C_15_H_31_	249	2.5	0.62±0.30	0.86±0.04	3.9±3.4	48±31
**7 w**	d4T	–	C_4_H_9_	n.h.^[c]^	>30	0.95±0.51	0.29±0.15	36±29	>100
**7 x**	d4T	–	C_11_H_23_	n.h.^[c]^	>30	0.89±0.28	0.26±0.083	16±5	78±10
**7 z**	d4T	–	C_18_H_37_	n.h.^[c]^	>30	0.11±0.042	0.11±0.084	0.05±0.027	86±3
**15 w**	T	–	C_4_H_9_	n.h.^[c]^	>30	>21	>5	>21	21±0
**15 z**	T	–	C_18_H_37_	n.h.^[c]^	>30	>54	>5	>54	54±4
**5 a**	d4T	C_8_H_17_ (AB)	C_8_H_17_ (AB)	52	1.6	0.31±0.01	0.62±0.30	2.26±1.03	52±1
**5 b**	d4T	C_4_H_9_ (AB)	C_17_H_35_ (AB)	50	3.3	0.12±0.05	0.10±0.03	0.54±0.41	33±7
**1**	d4T	–	–	n.d.^[d]^	n.d.^[d]^	0.43±0.25	0.22±0.05	>50	>50
**2 t**	d4TTP	–	–	n.d.^[d]^	n.d.^[d]^	1.05±0.35	0.40±0.26	>100	>100

The hydrolysis experiments of γ‐alkyl‐NTP **6**,**7** were performed in aqueous 25 mm phosphate buffer (PBS, pH 7.3) and in CEM/0 cell extracts; [a] Antiviral activity of the compounds in CD4^+^ T‐lymphocyte CEM cell cultures: 50 % effective concentration; values are the mean±SD of *n*=2–3 independent experiments. [b] Cytotoxicity: 50 % cytostatic concentration or compound concentration required to inhibit CD4^+^ T‐cell (CEM) proliferation by 50 %; values are the mean±SD of *n*=2–3 independent experiments. [c] n.h.: no hydrolysis observed through the cleavage of AB‐mask. [d] n.d.: not determined.

### Chemical Stability in Aqueous Phosphate Buffer (PBS, pH 7.3)

**Figure 2:**
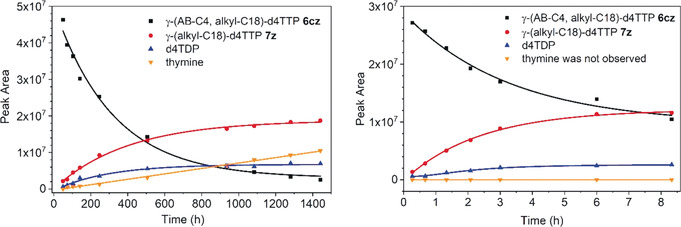
Hydrolysis study of **6 cz** in PBS (pH 7.3) and CEM/0 cell extracts.

In PBS, the half‐lives of γ‐(AB,alkyl)‐d4TTP prodrugs **6** were found to be between 94 h and 269 h without showing a clear trend. The lowest half‐life was observed for compound **6 cw** comprising short alkyl residues in the AB group and as the alkyl moiety (94 h, Table 1). As hydrolysis products, a predominant formation of γ‐alkyl‐d4TTPs **7** and a very small amount of d4TDP **2 m** was detected (approximately 3 %). After consumption of the starting material, no further increase of d4TDP concentration was observed. This supports the hypothesis that only the double‐γ‐esterified d4TTP prodrugs **6** were prone to a bond breakage between the γ‐ and β‐phosphate. As an example, the hydrolysis of compound **6 cz** is shown in Figure 2 A. The formed γ‐alkyl‐d4TTPs **7** proved to be entirely stable at pH 7.3 in PBS (Table 1). In contrast to previous studies with Tri*PPP*ro‐NTPs **5** containing a second cleavable mask (first generation NTP prodrugs),[^23^, ^24^, ^25^] no d4TTP was detected in these studies using **6** or **7**. A side reaction occurred during the studies using compounds **7** that led to the formation of thymine by the cleavage of the glycosidic bond as reported before.[^19^, ^22^] As shown in Figure SI1 in the Supporting information, this is the main reason for the decrease of **7 z** in PBS hydrolysis and the calculated half‐life of **7 z** is about 1500 h.

### Hydrolysis Study Using Esterase

Next, the enzymatic stability of prodrugs γ‐(AB‐C4,alkyl‐C18)‐d4TTP **6 cz** and γ‐(AB‐C15,alkyl‐C4)‐d4TTP **6 ew** was examined by incubation with PLE, which is used as a model enzyme for studying the esterase activation of the prodrugs, in PBS, pH 7.3 at 37 °C. The cleavage of the acyloxybenzyl masking unit in **6 cz** occurred readily under the experimental conditions (*t*_1/2_=8.1 min) leading selectively to the γ‐alkyl‐d4TTP derivative. For prodrug **6 ew** containing a long alkyl chain mask, the half‐life was found to be 25.5 min. This was in full agreement with the results from the studies of Tri*PPP*ro compounds **5** described before[^23^, ^24^] and proved a significant contribution of the enzyme. In these studies, no cleavage of the alkyl residue in compounds **7 w**,**z** was observed. This proves our initial concept of introducing an enzyme (esterase)‐stable group to the γ‐phosphate unit.

### Incubation of Compounds 6, 7, and 15 in Cell Extracts: A Comparison with the TriPPPro NTPs 5

The stability of prodrugs **6** and the γ‐alkyl‐NTPs **7** was further investigated in human CD_4_^+^ T‐lymphocyte CEM cell extracts. The half‐lives of prodrugs **6** were found to be significantly lower than the half‐lives in PBS buffer (up to 500‐fold; Table 1). Thus, an enzymatic cleavage reaction took place. Half‐lives as low as 0.4 h (**6 az**) to 5.2 h (**6 dz**) were determined. Here, compounds **6 az** and **6 bz** comprising an acetyl‐ or a propanoylester moiety, respectively, were found to be the least stable compounds (t_1/2_ of 0.4 and 1.6 h, respectively). This is in accordance to our previous results for the Di*PP*ro‐[^17^, ^18^] or the Tri*PPP*ro‐compounds **5**.[^22^, ^23^] In all cases we observed the predominant formation of γ‐alkyl‐d4TTPs **7** and d4TDP **2 d** in very low concentration (Figure 2 B) but no NTP formation was detected, in contrast to the studies using the previously described Tri*PPP*ro compounds **5**.[^23^, ^24^] However, the difference to the compounds bearing long lipophilic acyl groups was not very pronounced (2‐ to 3‐fold). In the case of γ‐(AB‐C4,alkyl‐C18)‐d4TTP **6 cz**, γ‐C18‐d4TTP **7 z** was detected as the main product. After 8 h incubation, the ratio of γ‐C18‐d4TTP **7 z** and d4TDP **2 d** was 5:1. This was in sharp contrast to the studies performed with Tri*PPP*ro compounds **5**.[^23^, ^24^] There, it was almost impossible to detect significant concentrations of d4TTP due to its fast dephosphorylation to form first d4TDP and finally d4TMP **2 m**. Even after very long incubation periods (up to 30 h), compounds **7** proved to be entirely stable, for example, 96 % of the thymidine bearing compound **15** was still detected after 30 h. The formation of thymine or d4TTP **2 t** from γ‐C18‐d4TTP **7 z** was not detected (Supporting Information, Figure SI2).

### Antiviral Evaluation

γ‐(AB,alkyl)‐d4TTP prodrugs **6** and the γ‐alkyl‐d4TTPs **7** were evaluated for their ability to inhibit the replication of HIV in HIV‐1‐ or HIV‐2‐infected wild‐type CEM/0 as well as mutant thymidine‐kinase‐deficient CEM cell cultures (CEM/TK^−^). For comparison, two Tri*PPP*ro derivatives **5** were also included in this assay. The results are summarized in Table 1. As can be seen, all compounds showed virtually similar or even slightly better activities against HIV‐1 and HIV‐2 than the parent nucleoside d4T **1**. Because the lipophilicity of compounds **6** is similar due to the combination of the introduced aliphatic alkyl groups in the masking unit as well as in the stable γ‐alkyl moiety, the antiviral activity did not differ much. More importantly almost all prodrugs **6** were also highly potent in CEM/TK^−^ cell cultures whereas d4T **1** lacked any relevant anti‐HIV activity in this thymidine‐kinase‐deficient cell line (EC_50_: 150 μm). Only prodrug **6 ey** (C15/C15) lost some of the activity displayed in the wild‐type CEM cell cultures compared to the other prodrugs, while **6 ew** (C15/C4) was surprisingly found to be inactive in the HIV‐2‐infected CEM/TK^−^ cell line. Also, prodrug **6 cw** (C4‐AB and C4 chain) showed a marked loss of activity in the CEM/TK^−^ cell cultures. This might be due to an insufficient lipophilicity of the compound combined with a relatively fast cleavage of the bioreversible moiety that led to the formation of short‐chain γ‐C4‐d4TTP **7 w**. Compound **7 w** was almost inactive in this assay. It should also be noticed that none of the prodrugs **6** were significantly more cytotoxic than the parent d4T **1** (Table 1). Interestingly, also the γ‐C18‐alkyl‐d4TTP **7 z** was highly potent against HIV‐2‐infected CEM/TK^−^ cell cultures (EC_50_: 0.055 μm). Surprisingly, compound **7 z** is the most active compound of all the listed derivatives (2700‐fold more active than d4T), although this compound is more negatively charged than the prodrugs. However, compound **7 z** is highly lipophilic due to the C18‐chain. Also, in the case of the Tri*PPP*ro compounds **5**, we found for the intermediates comprising a C17 aliphatic chain, antiviral activity in HIV‐2‐infected CEM/TK^−^ cells (1.5 μm).^[34]^ However, the antiviral activity of compound **7 z** was even improved by 30‐fold. Consequently, it seems that even one long aliphatic chain provides enough lipophilicity to enable the cellular uptake of the compounds. This is supported by the results obtained for compounds **7 w** (C4 chain) and **7 x** (C11 chain). Both compounds (as well as **6 ew**) showed a marked loss in antiviral activity in CEM/TK^−^ cell cultures (35 μm and 16 μm, respectively, Table 1). Most probably their lipophilicity is not sufficient to enable an effective uptake into the cells. As expected, both thymidine‐containing compounds **15 w** and **15 z** as well as d4TTP were found to be inactive in the CEM/TK^−^ cells. The two Tri*PPP*ro compounds bearing either two C8 chains (**5 a**) or a mixture of a short C4 and a long C17 alkyl group (**5 b**) were also active in the CEM/TK^−^ cells. Again, the compound bearing the C17 chain proved to be more active than the second one (C8 chain). However, both do not reach the excellent activity of compound **7 z**.

### Primer Extension Assays

All compounds described here showed significant antiviral activity against HIV‐1‐ and HIV‐2‐infected wild‐type CEM/O cell cultures. This activity was also virtually retained in CEM/TK^−^ cell cultures. Taking the results from the hydrolysis studies, it can be concluded that the delivered γ‐alkyl‐d4TTPs **7** were responsible for the inhibitory effect of these compounds because we have proven that no d4TTP was formed. This is a striking difference to the formerly reported Tri*PPP*ro compounds **5**.[^23^, ^24^] Further studies showed that the γ‐dimasked Tri*PPP*ro compounds **5** were not substrates for DNA polymerases, such as HIV‐RT or DNA polymerase β (unpublished results).

To shed more light on these results, primer extension assays were performed to investigate the substrate properties of the γ‐alkyl‐triphosphates for three different DNA polymerases: reverse transcriptase (RT; an HIV‐encoded RNA‐dependent DNA polymerase), cellular DNA polymerases α, β, and the mitochondrial DNA polymerase γ, which is often responsible for toxic side‐effects caused by nucleoside triphosphate analogues.[^28^, ^29^]

Two different set‐ups have been used. First, a mixture of dATP, dCTP, dGTP, and γ‐alkyl‐TTP (N*) or ‐d4TTP (T*) was used as a mixture. The results obtained from these experiments were compared to those in which just γ‐alkyl‐TTP (N) or d4TTP (T) was used. In each experiment a primer extension without added polymerase (negative control, ‐ lane) and an experiment in which all four canonical NTPs were added (positive control, +lane) were used as controls.

### Studies Using γ‐Alkyl‐TTPs 15 and γ‐Alkyl‐d4TTP 7 with HIV‐1 Reverse Transcriptase (RT)

**Figure 3:**
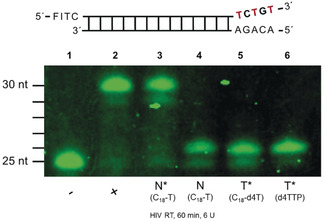
Primer extension assay using HIV‐RT and the γ‐alkyl‐dNTP **7** and **15**. Lane 1 (−): dATP, dGTP, dCTP, TTP without HIV RT; lane 2 (+): dATP, dGTP, dCTP, and TTP with HIV‐RT; lane 3 (N*, C_18_‐T): dATP, dGTP, dCTP, TTP, and γ‐C18‐TTP **15 z**; lane 4 (N, C_18_‐T): γ‐C18‐TTP **15 z**; lane 5 (T*, C_18_‐d4T): dATP, dGTP, dCTP, and γ‐C18‐d4TTP **7 z**; lane 6 (T*, d4TTP): dATP, dGTP, dCTP, and d4TTP. Assay condition: 50 mm Tris‐HCl (pH 8.6 at 22 °C), 10 mm MgCl_2_, 40 mm KCl, dNTPs 66 μm, HIV‐RT 6 U, primer‐template‐hybrid 0.32 μm in a reaction volume of 10 μL, incubated 37 °C for 60 min, 80 °C for 3 min; 50 mA, 45 w for 4 h.

Figure 3 summarizes the results of the primer extension using HIV‐RT and 60 minutes incubation time. No elongation occurred without HIV‐RT (lane 1). Full extension was observed when all four canonical triphosphates were present (lane 2). Replacement of TTP **4 t** by γ‐C18‐TTP **15 z** also led to full extension of the primer proving that HIV‐RT accepted the alkylated TTP as a substrate (lane 3). This was confirmed by the single incorporation assay using just γ‐C18‐TTP without the other three canonical triphosphates (*n*+1 band; lane 4). Next, γ‐C18‐d4TTP **7 z** was used in combination with the other three canonical triphosphates (lane 5). Only the *n*+1 band was detected because d4TMP was incorporated and acted as an immediate chain terminator. Lane 6 shows the result obtained when d4TTP **2 t** and the three other nucleoside triphosphates were used. Again, only the *n*+1 band is visible. Thus, both γ‐C18‐alkylated nucleoside triphosphates were accepted by HIV‐RT as a substrate, and were properly incorporated in the growing DNA chain.

Next, an identical series of experiments was conducted using 15 minutes of incubation time (Supporting Information, Figure SI3). The control experiments showed identical results as above (lane 1,2,7). However, in contrast to the first set of experiments only a very weak spot for the full extension product was detected when γ‐C18‐TTP **15 z** was used (lane 3). When γ‐C4‐TTP **15 w** was used, again clearly the full extension product (lane 4) was detected. Thus, the shorter alkyl chain led to a compound with much better substrate properties for HIV‐RT. Single incorporation experiments proved this as well (lane 5). γ‐C4‐d4TTP **7 w** in the mixture with the other three nucleoside triphosphates led to a single incorporation and an immediate chain termination. An incubation time of 30 minutes showed also for γ‐C18‐TTP **15 z** incorporation and elongation with strong stalls for incompletely elongated primers (Supporting Information, Figure SI4, lane 3). Again, single incorporation of γ‐C18‐TTP **15 z** proceeded but some of the starting primer was still present (lane 4). Incorporation of d4TMP from γ‐C18‐d4TTP **7 z** again led to an immediate chain termination (lane 5).

Finally, a comparison of the three γ‐alkyl‐modified d4TTP was performed (Supporting Information, Figure SI5). Different incubation periods were studied as well. The first two lanes are the control reactions. In all cases, the conversion of the primer to the *n*+1 product continued over time. Again, γ‐C4‐d4TTP **7 w** and γ‐C11‐d4TTP **7 x** were found to be better substrates for HIV‐RT compared to γ‐C18‐d4TTP **7 z**.

### Studies Using γ‐Alkyl‐TTPs 15 and γ‐Alkyl‐d4TTP 7 with Human DNA Polymerase β (Pol β)

**Figure 4:**
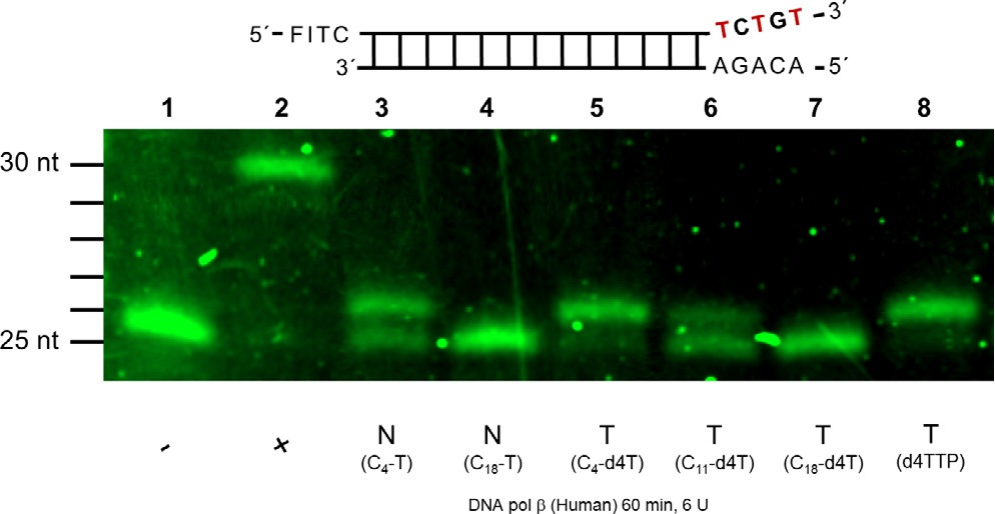
Primer extension assay using DNA polymerase β and the γ‐alkyl‐dNTP **7** and **15**. Lane 1 (−): dATP, dGTP, dCTP and TTP without human DNA polymerase β; lane 2 (+): dATP, dGTP, dCTP, and TTP with human Pol β; lane 3 (N, C_4_‐T): γ‐C4‐TTP **15 w**; lane 4 (N, C_18_‐T): γ‐C18‐TTP **15 z**; lane 5 (T, C_4_‐d4T): γ‐C4‐d4TTP **7 w**; lane 6 (T, C_11_‐d4T): γ‐C11‐d4TTP **7 x**; lane 7 (T, C_18_‐d4T): γ‐C18‐d4TTP **7 z**; lane 8 (d4TTP): d4TTP. Assay condition: 50 mm Tris‐HCl (pH 8.7), 10 mm MgCl_2_, 100 mm KCl, 1.0 mm dithiothreitol, 0.4 mg mL^−1^ of bovine serum albumin, 15 % glycerol, dNTPs 66 μm, DNA polymerase β 6 U, hybrid 0.32 μm in a reaction volume of 10 μL, incubated 37 °C for 60 min, 80 °C for 3 min; 50 mA, 45 w for 4 h.

The results obtained for HIV‐RT were compared to primer extension assays using human DNA polymerase β (Figure 4).

The control experiments proved that the experimental set‐up was functional (lanes 1,2). d4TTP was efficiently incorporated into the primer by DNA polymerase β and caused an immediate chain termination (lane 8). However, it was interesting to observe that with γ‐C18‐d4TTP **7 z** no incorporation was detected (lane 7), γ‐C11‐d4TTP **7 x** was a substrate (lane 6) and γ‐C4‐d4TTP **7 w** behaved as a good substrate (lane 5) for the polymerase (lanes 3–7). The same was observed with γ‐C18‐TTP **15 z** (lane 4, no incorporation) and γ‐C4‐TTP **15 w** (lane 3, about 60 % incorporation). Interestingly, γ‐C4‐d4TTP **7 w** seems to be a more effective substrate than γ‐C4‐TTP **15 w** (lanes 3 and 5, respectively).

### Studies Using γ‐Alkyl‐TTP 15 and γ‐Alkyl‐d4TTP 7 with Human DNA Polymerase γ (Pol γ)

In contrast to the results obtained with DNA polymerase β, all compounds **15** (thymidine analogue) and **7** (d4TTP analogue) as well as d4TTP itself were not substrates for the mitochondrial DNA polymerase γ (Supporting Information, Figure SI6). Only the primer was detected after the assay. Thus, this polymerase does not tolerate any modification at the γ‐phosphate moiety.

### Studies Using γ‐Alkyl‐TTP 15 and γ‐Alkyl‐d4TTP 7 with Human DNA Polymerase α (Pol α)

More importantly, all γ‐modified compounds **7** and **15** were also not substrates for DNA polymerase α, which is one of the most important replication polymerases. In contrast, nucleoside analogues without a γ‐phosphate alkyl residue were incorporated by the polymerase in our assays. (Supporting Information, Figure SI7).

Consequently, nucleoside triphosphates modified at the γ‐position are substrates for HIV‐RT, while they fail to act as substrates when the modification has a sufficiently high size/length. Thus, by these structural changes, d4TTP, which was originally a substrate for DNA polymerase α and β, can be converted into a non‐substrate for this enzyme, while still being a substrate for HIV‐RT.

## Conclusion

In summary, we have disclosed a second generation of lipophilic nucleoside triphosphate prodrugs which differ from the previously described Tri*PPP*ro derivatives[^23^, ^24^, ^25^] by comprising a non‐cleavable moiety in addition to a biodegradable prodrug moiety at the γ‐phosphate group. As non‐cleavable group lipophilic alkyl residues of different lengths were attached in addition to the previously used acyloxybenzyl‐prodrug moiety. The synthesis was achieved in satisfying chemical yields using *H*‐phosphonate chemistry. We have proven that the prodrug group was selectively cleaved to give γ‐alkyl‐modified nucleoside triphosphates by chemical hydrolysis (slow process) or by enzymes present in cell extracts (fast process). The γ‐alkyl‐nucleoside triphosphates proved to be stable in cell extracts for at least 30 h while the corresponding nucleoside triphosphates d4TTP or TTP were rapidly enzymatically dephosphorylated. In contrast to d4T itself, all these compounds showed marked antiviral activity against HIV‐2 in thymidine‐kinase‐deficient cell cultures (CEM/TK^−^ cells) indicating the successful cell membrane passage of these compounds. Thus, although such second‐generation nucleoside triphosphate prodrugs are still negatively charged at the phosphate groups, obviously the modification at the γ‐phosphate group by one lipophilic, bioreversible moiety and the stable γ‐alkyl group gives the molecule sufficient lipophilicity to penetrate the cell membrane. γ‐Alkylated‐TTPs were substrates for HIV‐RT as shown in primer extension assays, while they proved not to be substrates for the cellular DNA polymerases α, β, and γ. The corresponding d4TTP derivatives were also found to be substrates for HIV‐RT but were still not substrates for DNA polymerases depending on the alkyl chain length. Thus, these results point to an increased selectivity of the compounds to different polymerases with a marked preference for the viral enzyme HIV‐RT.

Thus, here we have disclosed the development of an advanced prodrug concept for NTP derivatives that delivers γ‐alkyl‐NTPs with high selectivity by an enzyme‐triggered mechanism that then allows i) the bypass of all phosphorylation steps usually needed for the activation of a nucleoside analogue, ii) the delivery of compounds which act as substrates for a viral polymerase (RT) but not substrates for cellular DNA polymerases, and iii) which show very high stability against dephosphorylation of the triphosphate unit compared to natural NTPs. We are currently working on whether this increased selectivity towards the viral polymerase is a general property of these γ‐modified compounds by using different nucleoside analogues and different viral polymerases.

**Please note**: Minor changes have been made to this manuscript since its publication as an Accepted Article. The Editor.

## Conflict of interest

The authors declare no conflict of interest.

## Supporting information

As a service to our authors and readers, this journal provides supporting information supplied by the authors. Such materials are peer reviewed and may be re‐organized for online delivery, but are not copy‐edited or typeset. Technical support issues arising from supporting information (other than missing files) should be addressed to the authors.

Supplementary
