# Supplementary material for: Prodrugs of γ‐Alkyl‐Modified Nucleoside Triphosphates: Improved Inhibition of HIV Reverse Transcriptase
Source: Angew Chem Int Ed Engl. 2020 Sep 30;59(49):22063–71. doi: 10.1002/anie.202003073 (PMC7756582; doi:10.1002/anie.202003073)
Supplement: Supplementary file 1 — Supplementary [file ANIE-59-22063-s001.pdf]

Angewandte Chemie
Eine Zeitschrift der Gesellschaft Deutscher Chemiker

Supporting Information

Prodrugs of γ-Alkyl-Modified Nucleoside Triphosphates: Improved Inhibition of HIV Reverse Transcriptase

*Chenglong Zhao, Stefan Weber, Dominique Schols, Jan Balzarini, and Chris Meier**

anie_202003073_sm_miscellaneous_information.pdf

Table of Contents

General Experimental Procedures 2
Syntheses and characterization 5
Results and Discussion 35
Scheme SI1 35
Figure SI1 35
Figure SI2 36
Figure SI3 36
Figure SI4 37
Figure SI5 37
Figure SI6 38
Author Contributions 38

Experimental Procedures

General: All reactions were carried out under dry conditions and at room temperature.

Solvents and Reagents: CH_3CN, THF and DMF were purchased from Acros Organics (Extra Dry over molecular sieves) and dried with activated molecular sieves. Triethylamine (NEt_3) was refluxed over CaH_2 for three days and distilled under nitrogen. All further reagents commercially available were used as received. *Thin layer chromatography (TLC):* For thin layer chromatography Macherey-Nagel pre-coated TLC sheets Alugram® Xtra SIL G/UV254 were used. Phosphomolybdic Acid (PMA) stain was used and prepared by dissolving 10 g of phosphomolybdic acid in 100 mL of ethanol. *Column chromatography:* Normal phase column chromatography were performed with Macherey-Nagel silica gel 60 M (0.04-0.063 mm). *Automatic RP-18 chromatography:* For reversed phase chromatography an Interchim Puriflash 430 in combination with Chromabond® Flash RS40 C_{18} ec was used. *High Performance Liquid Chromatography (HPLC)*: HPLC was required for analytical studies and monitoring reactions. A VWR-Hitachi LaChromElite HPLC system (L-2130, L-2200, L-2455), EzChromElite software and equipped with a Nucleodur 100-5 C_{18}ec or Nucleodur 100-5 C_8ec (Macherey-Nagel) was available. Acetonitrile for HPLC was obtained from VWR (HPLC grade) and ultrapure water was produced by a Sartorius Aurium® pro (Sartopore 0.2 µm, UV). 2 mM tetra-*n*-butylammonium acetate solution (TBAA, pH 6.3) or 10 mM triethylammonium acetate (TEAA, pH 6.2) were used for buffering. Method: Nucleodur 100-5 C18ec; 0-20 min: TBAA buffer/acetonitrile gradient (5-80%); 20-30 min: buffer/acetonitrile (80%); 30-33 min: buffer/acetonitrile (80-5%); 33-38 min: buffer/acetonitrile (5%); flow: 1 mL/min.

Nuclear Magnetic Resonance (NMR): NMR spectra were recorded at room temperature in automation mode with a Varian Gemini 2000BB, Bruker Fourier 300, Bruker AMX 400, Bruker DRX 500 or Bruker AVIII 600. All ^{1}H- and ^{13}C-NMR chemical shifts (δ) are quoted in parts per million (ppm) downfield from tetramethylsilane (TMS) and calibrated on solvent signal. The ^{31}P-NMR chemical shifts (proton decoupled) are also quoted in ppm using phosphoric acid as the external standard. *Mass Spectrometry (MS)*: HRMS (ESI) mass spectra were acquired with a VG Analytical Finnigan ThermoQuest MAT 95 XL or an Agilent 6224 EIS-TOF spectrometer. MALDI measurements (matrix: 9-aminoacridine [9AA] or 2,5-dihydroxybenzoic acid [DHB]) were performed with a Bruker UltrafelXtreme spectrometer. *Infrared spectroscopy (IR)*: IR spectra were recorded on a Bruker Alpha P FT-IR at room temperature in the range of 400-4000 cm^{-1}. *Freeze dryer*: Alpha 2-4 LDPlus freeze dryer from Christ Co. and chemistry hydrid pump RC6 from Vacuumbrand Co. was used. *Ultrapure water*: Arium®pro ultrapure water system was used. *pH meter*: pH value was tested with

ProLab 300 from Schott Co.. *Thermomixer*: Eppendorf Thermomixer 5436 and CellMedia Thermoschüttler basic were used. *Fluorescent Imager*: ChemiDoc™ MP Imaging System (170-8280) from Bio-Rad Co. was used for primer extension study. *Electrophoresis*: Thermo Fisher OwlTM S4S Aluminum-Backed Sequencer System. Power supply: Consort EV2230 Gel Electrophoresis (1500 V, 300 mA, 150 W).

Chemical hydrolysis of prodrugs of γ-(AB,alkyl) d4TTP 6 as well as the γ-alkyl-NTPs 7,15:

Stock solutions (50mM in DMSO-d_6) of compounds **6**,**7** were prepared. After dilution of 11 µL stock solution with 100 µL milliQ water and 189 µL DMSO-d_6 to 1.9mM hydrolysis solutions the reaction was started by addition of 300 µL phosphate buffer saline (PBS, 50mM, pH 7.3). The solution was incubated at 37 C in a thermomixer. An initial aliquot (25 µL) was taken directly and analyzed by analytical HPLC at 265-266 nm. Further aliquots were taken for monitoring the kinetic hydrolysis. The exponential decay curves (pseudo-first order) based on absolute integral values were calculated with commercially available software (OriginPro 9.0G) and yielded the half-lives $t_{1/2}$ of the prodrugs via one determination.

Enzymatic hydrolysis of compounds 6,7 with *pig liver esterase* (PLE):

10 µL 50 mM DMSO stock solution of compounds **6**,**7** was diluted to 6.0 mM hydrolysis solution by addition of 31.7 µL DMSO and 41.7 µL ultrapure water. Then 83.3 µl of the 6.0 mM solution was diluted with 125 µL DMSO and 833 µl 50 mM PBS buffer (pH 7.3). The reaction was started by addition of 62.5 µl of PLE in PBS buffer (3 mg/mL) and the mixture was incubated with 800 rpm at 37 °C in a thermomixer. At different times, aliquots (100 µl) were taken and the reaction was stopped by addition to 106 mL MeOH. The mixture was kept for 5 min on ice followed by centrifugation for 5 min (13000 rpm). The mixture was filtered (Chromafil RC-20/15 MS, 0.2 mm) and stored in liquid nitrogen. When testing, the samples were defrosted and injection volume with 80 µL was used for HPLC analysis.

Enzyme-catalyzed hydrolysis of γ-(AB,alkyl)-d4TTPs 6, γ-alkyl-d4TTPs 7,15 and Tri*PPP*ros 5 in CEM cell extracts:

18 µL of the appropriate 50 mM DMSO-d_6 stock solution was diluted to 6.0 mM hydrolysis solution by addition of 132.0 µL DMSO-d_6. 7-10 different samples including 10 µL water and 10 µL hydrolysis solution were prepared in 2 mL Eppendorf® vials. The reaction was started by addition of 50 µL human CEM cell extract and the mixture was incubated at 37 °C for different time periods. The reactions were stopped by addition of 150 µL MeOH. The solution was kept on ice for 5 min followed by centrifugation for 5 min (13000 rpm). The supernatants were filtered (Chromafil® RC-20/15 MS, 0.2 µm) and stored in liquid nitrogen. When testing, the samples were defrosted and injection volume with 80 µL was used for HPLC analysis. The calculation of t1/2 was performed analogously to that for the chemical hydrolysis studies.

Preparation of cell extracts: Human CD_4^+ T-lymphocyte CEM cells were grown in RPMI-1640-based cell culture medium to a final density of ~$3{\cdot}10^6$ cells/ml. Then, cells were centrifuged for 10 min at 1,250 rpm at 4 °C, washed twice with cold PBS, and the pellet was re-suspended at 10^8 cells/ml and sonicated (Hielscher Ultrasound Techn., 100% amplitude, 3·times for 10 sec) to destroy cell integrity. The resulting cell suspension was then centrifuged at 10,000 rpm to remove cell debris, and the supernatant divided in aliquots before being frozen at -80 C and used.

Anti-HIV activity assay: Inhibition of HIV-1(III_B)- and HIV-2(ROD)-induced cytopathicity in wild-type CEM/0 and thymidine kinase-deficient CEM/TK^- cell cultures was measured in microtiter 96-well plates containing ~$3 \cdot 10^5$ CEM cells/mL infected with 100 $CCID_{50}$ of HIV per milliliter and containing appropriate dilutions of the test compounds. After 4−5 days of incubation at 37 °C in a CO_2-controlled humidified atmosphere, CEM giant (syncytium) cell formation was examined microscopically. The EC_{50} (50% effective concentration) was defined as the compound concentration required to inhibit HIV-induced giant cell formation by 50%.

Primer-extension reactions:

HIV-RT and human DNA polymerase β and γ were obtained from Roboklon, human DNA polymerase α was obtained from Chimerx. The primers and templates were purchased from Life Technologies and Microsynth. The fluorescent labeled primer was purchased from Metabion and Microsynth. The gels were prepared with the size of 450mm×200mm×0.4mm according to the electrophoresis apparatus.

5'-FITC and 5'-Cy3 labeled Primer-sequence:

5´-FITC-CGTTG GTCCT GAAGG AGGAT AGGTT-3´

5´-Cy3-CGTTG GTCCT GAAGG AGGAT AGGTT-3´

Template-sequence:

3´-GCAAC CAGGA CTTCC TCCTA TCCAA AGACA-5´

The primer extension assays were performed under the following conditions:

FITC and Cy3 labeled primer extension assay:

1) Hybridization: After 5 min incubation at 95 °C in 20 mM Tris-HCl (pH 7.6) and 50 mM NaCl, the hybridization/annealing of the primer to the template strand was achieved by a cooling phase from 95 °C to -20 °C lasted more than 2 hours.

2) For HIV- RT assay: The final assay solution (10 µL) consists of 50 mM Tris-HCl (pH 8.6 at 22 °C), 10 mM $MgCl_2$, 40mM KCl, dNTPs 66 µM, HIV RT 6 U, Hybrid 0.32 µM in a reaction volume of 10 µL, incubated 37 °C for 15 min, 80 °C for 3 min; 50 mA, 45w for 4 h. The assays were separated using a denaturating PAGE (15%). The result was visualized by fluorescence imaging.

3) For human DNA pol β assay: The final assay solution (10 µL) consists of 50 mM Tris-HCl (pH 8.7), 10 mM $MgCl_2$, 100mM KCl, 1.0 mM dithiothreitol, 0.4 mg/ml of bovine serum albumin, 15% glycerol, dNTPs 66 µM, DNA Polymerase Beta 6 U, Hybrid 0.32 µM in a reaction volume of 10 µL, incubated 37 °C for 60 min, 80 °C for 3 min; 50 mA, 45w for 4 h. The assays were separated using a denaturating PAGE (15%). The result was visualized by fluorescence imaging.

4) For human DNA pol α assay: The final assay solution (25 µL) consists of 60 mM Tris-HCl (pH 8), 5 mM MgOAc, 100mM KCl, 1.0 mM dithiothreitol, 0.01% (w/v) of bovine serum albumin, dNTPs 250 µM, DNA Polymerase alpha 4U, Hybrid 0.20 µM in a reaction volume of 25 µL, incubated 37 °C for 5 min without dNTPs, then incubated 60 min at 37°C, 80 °C for 3 min; 50 mA, 45w for 3 h. The assays were separated using a denaturating PAGE (15%). The result was visualized by fluorescence imaging.

5) For human DNA pol γ assay: The final assay solution (25 µL) consists of 60 mM Tris-HCl (pH 8), 5 mM MgOAc, 100mM KCl, 1.0 mM dithiothreitol, 0.01% (w/v) of bovine serum albumin, dNTPs 250 µM, DNA Polymerase gamma 4U, Hybrid 0.20 µM in a reaction volume of 25 µL, incubated 37 °C for 5 min without dNTPs, then incubated 60 min at 37°C, 80 °C for 3 min; 50 mA, 45w for 3 h. The assays were separated using a denaturating PAGE (15%). The result was visualized by fluorescent imaging.

Syntheses and characterization

The syntheses and characterization of 4-(hydroxymethyl)phenylalkanoates **9** were described previously. The synthesis of (*n*-$Bu_4N)_2$▪d4TMP **2m** was performed using the Sowa-Ouichi procedure starting from d4T **1**.

General Procedure 1: Preparation of mixed *H*-phosphonate 10

Under dry conditions, diphenyl phosphonate (1.2 equiv.) was dissolved in 3 mL pyridine and cooled to 0 °C. Aliphatic alcohol **8** (1.0 equiv.) was added and stirred at 0 °C for 30 min and then heated up to 38 °C. Following, 4-(hydroxymethyl)phenylalkanoate **9** (1.4 equiv.) was added and the mixture was stirred for 3 h. Then the solvent was removed in vacuum. The crude product was purified by flash column chromatography (silica) with EtOAc/petroleum ether/0.5% acetic acid as eluent.

General Procedure 2: Preparation of γ-(AB,alkyl)-d4TTPs 6

The reactions were performed under dry conditions. a) *H*-phosphonate (1.0 equiv.) was dissolved in 6 mL CH_3CN and *N*-chlorosuccinimide (2.0 equiv.) was added. After stirring for 2 h at room temperature, tetrabutylammonium phosphate monobasic solution (0.4 M in acetonitrile) (3.0 equiv.) was added dropwise. The mixture was stirred for 1 h and the solvent was removed in vacuum. The residue was extracted with CH_2Cl_2/H_2O. The organic phase was dried over sodium sulfate and the solvent was removed by evaporation to afford corresponding pyrophosphate in nearly quantitative yield. b) The corresponding pyrophosphate was dissolved in CH_3CN and cooled down to 0 °C. A mixture of trifluoroacetic anhydride (TFAA, 5.0 equiv.) and Et_3N (8.0 equiv.) in 3 mL CH_3CN was cooled to 0 °C and added to the mixture. After stirring for 10 min, all volatile components were removed in vacuum. The residue was once again co-evaporated with 3 mL CH_3CN and subsequently dissolved in 3 mL CH_3CN at 0 °C. 1-methylimidazole (3.0 equiv.) and Et_3N (Et_3N, 8.0 equiv.) was added. The suspension was warmed up to room temperature and stirred for 10 min. The resulting activated imidazolidate formed and the corresponding NMP (0.7-1.0 equiv.) in 3 mL CH_3CN was added. The reaction was stirred at room temperature for 2-3 h and dried in vacuum. The crude product was purified by automatic RP18 flash chromatography, and then followed by ion-exchange to the ammonium form with Dowex 50WX8 cation-exchange resin and a second RP18 chromatography purification step. Product-containing fractions were collected and the organic solvent evaporated. The remaining aqueous solutions were freeze-dried and the desired product obtained as a white cotton

Synthesis of non-symmetric *H*-phosphonates 10 and γ-modified NTPs 6,7,15

(AB-CH_3,alkyl-$C_{18}H_{37}$)-*H*-phosphonate 10az

According to general procedure 1, with 0.23 mL diphenyl phosphonate (1.2 mmol, 1.2 equiv.) at 0 °C, 0.27 g 1-octadecanol **8z** (1.0 mmol, 1.0 equiv.) was added and following with 0.23 g 4-(hydroxymethyl)phenylacetate **9a** (1.4 mmol, 1.4 equiv.). Reaction time was 3 h at room temperature. Column chromatography (SiO_2, petrol ether/ethylacetate/CH_3COOH 8:2:0.005 v/v/v). Yield: 48%, 0.23 g, as white solid. **^{1}H-NMR** (600 MHz, $CDCl_3$-d_1): δ 7.41 (d, *J* = 8.4 Hz, 2H), 7.10 (d, *J* = 8.5 Hz, 2H), 6.87 (d, *J* = 696 Hz, 1H), 5.09 (d, *J* = 9.4 Hz, 2H), 4.10-3.93 (m, 2H), 2.30 (s, 3H), 1.64 (quint, *J* = 6.9 Hz, 2H), 1.42-1.18 (m, 30H), 0.88 (t, *J* = 7.0 Hz, 3H). **^{13}C-NMR** (151 MHz, $CDCl_3$-d_1): *δ* 169.27, 150.81, 133.32 (d, $^2J_{CP}$ = 6.0 Hz), 129.16, 121.88, 66.49 (d, $^2J_{CP}$ = 5.5 Hz), 66.02 (d, $^2J_{CP}$ = 6.1 Hz), 30.34 (d, $^3J_{CP}$ = 6.3 Hz), 31.91, 29.68, 29.66, 29.64, 29.62, 29.54, 29.47, 29.34, 29.08, 25.44, 22.68, 21.09, 14.10. **^{31}P-NMR** (243 MHz, $CDCl_3$-d_1): *δ* 7.73. **HRMS (ESI-TOF) m/z**: calculated for $C_{27}H_{47}NaO_5P$ $[M+Na]^+$ 505.3053, found 505.3049. **IR**: ν [cm^{-1}] =.2955, 2918, 2870, 2847, 1754, 1609, 1510, 1460, 1420, 1373, 1232, 1200, 1110, 1085, 1052, 1021, 989, 918, 877, 853, 816, 784, 752, 724, 704, 659, 597, 555, 515, 502, 493, 479, 451, 427.

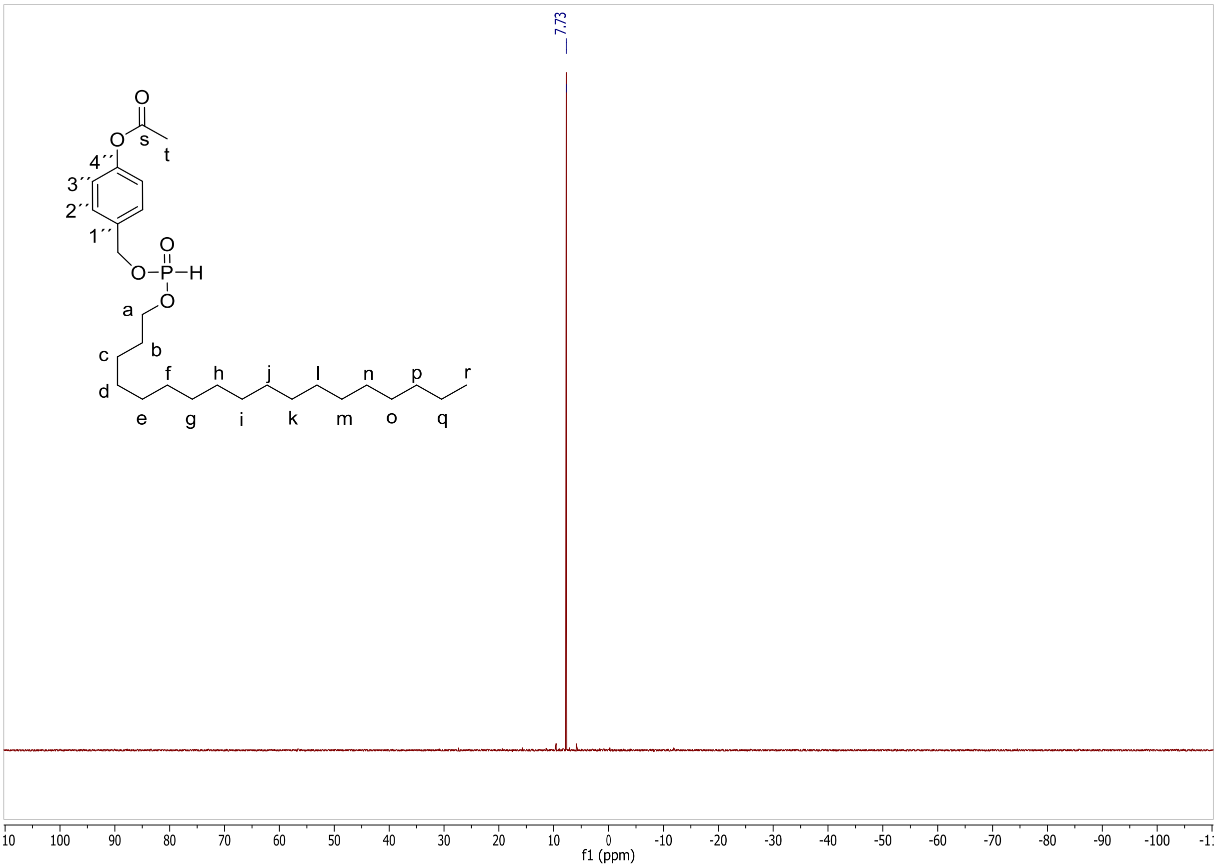

(AB-C_2H_5,alkyl-$C_{18}H_{37}$)-*H*-phosphonate 10bz

According to general procedure 1, with 0.23 mL diphenyl phosphonate (1.2 mmol, 1.2 equiv.) at 0 °C, 0.27 g 1-octadecanol **8z** (1.0 mmol, 1.0 equiv.) was added and following with 0.25 g 4-(hydroxymethyl)phenylpropionate **9b** (1.4 mmol, 1.4 equiv.). Reaction time was 3 h at room temperature. Column chromatography (SiO_2, petrol ether/ethylacetate/CH_3COOH 8:2:0.005 v/v/v). Yield: 52%, 0.26 g, as white solid. **^{1}H-NMR** (600 MHz, $CDCl_3$-d_1): δ 7.40 (d, *J* = 8.3 Hz, 2H), 7.18-7.02 (m, 2H), 6.87 (d, *J* = 702 Hz, 1H), 5.09 (d, *J* = 9.5 Hz, 2H), 4.11-3.92 (m, 2H), 2.59 (q, *J* = 7.5 Hz, 2H), 1.64 (quint, *J* = 6.7 Hz, 2H), 1.45-1.13 (m, 33H), 0.87 (t, *J* = 6.9 Hz, 3H). **^{13}C-NMR** (151 MHz, $CDCl_3$-d_1): *δ* 172.7, 150.9, 133.2, 129.1, 121.9, 66.50 (d, *J* = 5.5 Hz), 65.98 (d, *J* = 6.1 Hz), 30.32 (d, *J* = 6.2 Hz), 31.89, 29.67, 29.64, 29.63, 29.61, 29.52, 29.45, 29.33, 29.06, 27.69, 25.43, 22.66, 14.08, 8.99. **^{31}P-NMR** (243 MHz, $CDCl_3$-d_1): *δ* 7.70. **HRMS (ESI-TOF) m/z**: calculated for $C_{28}H_{53}NO_5P$ $[M+NH_4]^+$ 514.3656, found 514.3663. **IR**: ν [cm^{-1}] = 2955, 2919, 2847, 1753, 1598, 1509, 1460, 1422, 1384, 1358, 1254, 1241, 1222, 1202, 1173, 1158, 1109, 1087, 992, 903, 870, 830, 803, 770, 753, 725, 567, 551, 531, 515, 478, 446, 426, 419.

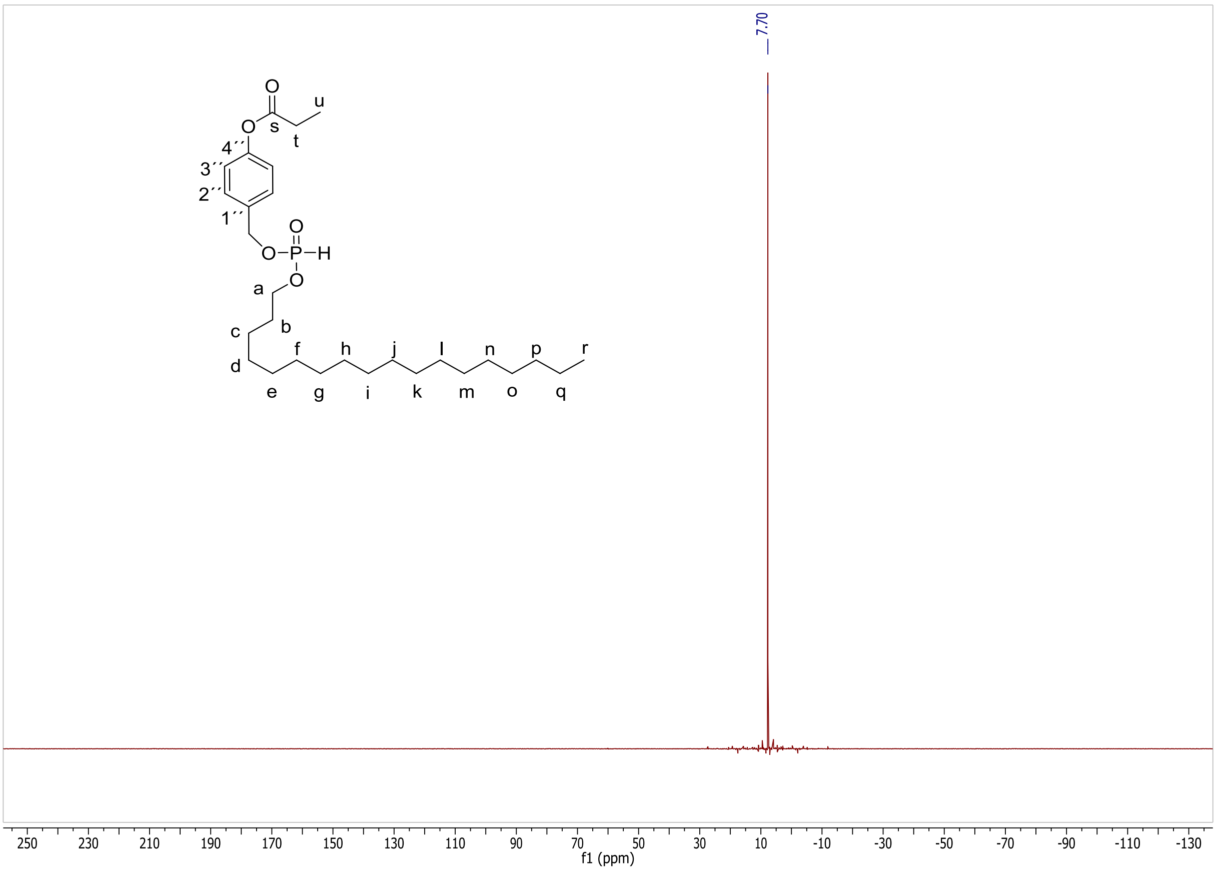

(AB-C_4H_9,alkyl-C_4H_9)-*H*-phosphonate 10cw

According to general procedure 1, with 0.23 mL diphenyl phosphonate (1.2 mmol, 1.2 equiv.) at 0 °C; 0.21 g 4-(hydroxymethyl)phenylpentanoate **9c** (1.0 mmol, 1.0 equiv.) was added and following with 0.10 g 1-butanol **8w** (1.4 mmol, 1.4 equiv.). Reaction time was 3 h at room temperature. Column chromatography (SiO_2, petrol ether/ethyl acetate/CH_3COOH 2:8:0.005 v/v/v). Yield: 31%, 0.103 g, as colorless oil. **^{1}H-NMR** (500 MHz, $CDCl_3$-d_1): δ 7.41 (d, *J* = 8.5 Hz, 2H), 7.09 (d, *J* = 8.5 Hz, 2H), 6.87 (d, *J* = 700 Hz, 1H), 5.09 (d, *J* = 9.6 Hz, 2H), 4.11-3.96 (m, 2H), 2.56 (t, *J* = 7.5 Hz, 2H), 1.74 (quint, *J* = 7.6 Hz, 2H), 1.64 (quint, *J* = 6.7 Hz, 2H), 1.50-1.33 (m, 4H), 0.97 (t, *J* = 7.4 Hz, 3H), 0.92 (t, *J* = 7.4 Hz, 3H). **^{13}C-NMR** (126 MHz, $CDCl_3$-d_1): *δ* 172.12, 150.92, 133.20, 129.15, 121.89, 66.53 (d, $^{2}J_{CP}$ = 5.5 Hz), 65.67 (d, $^{2}J_{CP}$ = 6.2 Hz), 34.07, 32.30 (d, $^{3}J_{CP}$ = 6.3 Hz), 26.94, 22.22, 18.66, 13.70, 13.50. **^{31}P-NMR** (162 MHz, $CDCl_3$-d_1): *δ* 7.70. **HRMS (ESI-TOF) m/z**: calculated for $C_{16}H_{25}NaO_5P$ $[M+Na]^+$ 351.1332, found 351.1338. **IR**: v [cm^{-1}] = 2959, 2933, 2872, 1756, 1612, 1508, 1464, 1418, 1379, 1200, 1165, 1141, 1078, 974, 920, 850, 827, 510, 434.

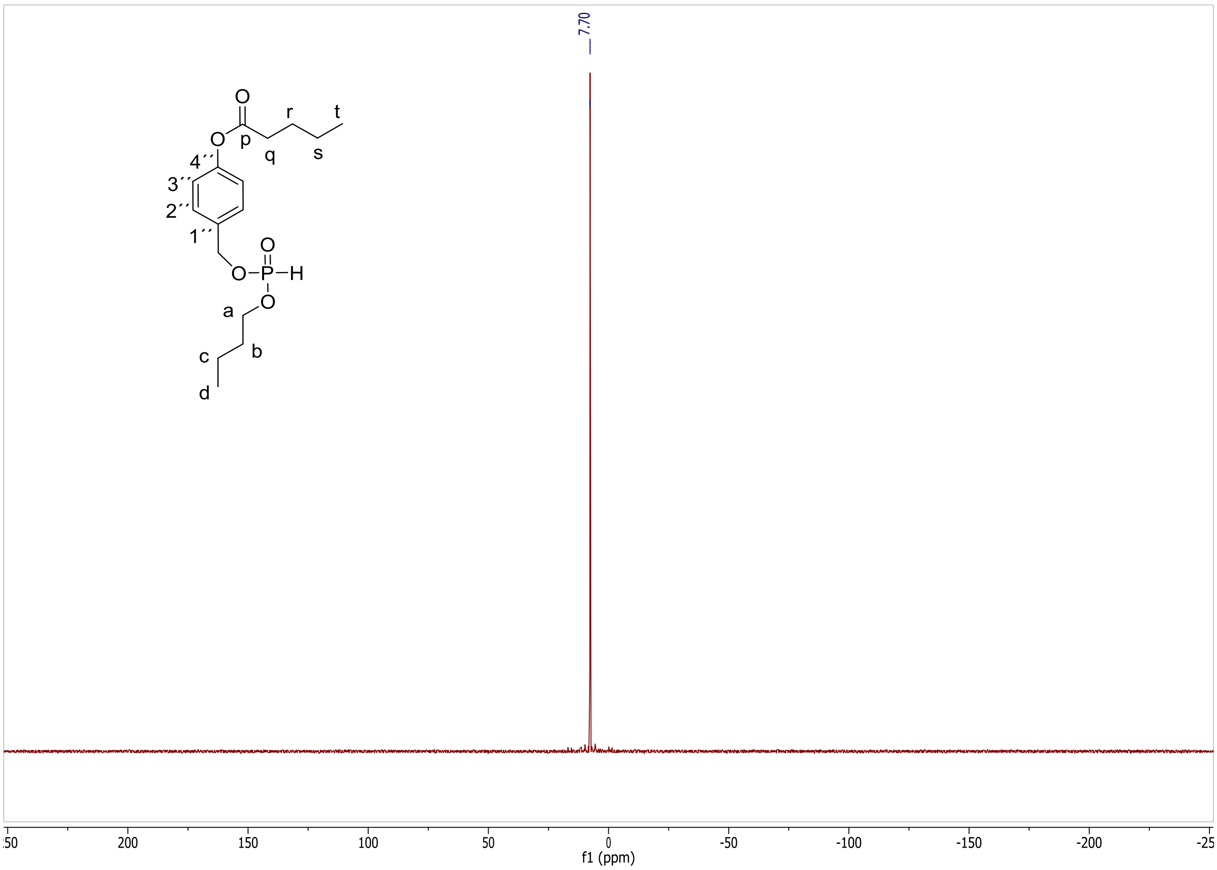

(AB-C_4H_9,alkyl-$C_{15}H_{31}$)-*H*-phosphonate 10cy

According to general procedure 2, with 0.23 mL diphenyl phosphonate (1.2 mmol, 1.2 equiv.) at 0 °C; 0.23 g 1-pentadecanol **8y** (1.0 mmol, 1.0 equiv.) was added followed by 0.29 g 4-(hydroxymethyl)phenylpentanoate **9c** (1.4 mmol, 1.4 equiv.). Reaction time was 3 h at room temperature. Column chromatography (SiO_2, petrol ether/ethylacetate/CH_3COOH 7:3:0.005 v/v/v). Yield: 43%, 0.208 g, as white solid. **^{1}H-NMR** (600 MHz, $CDCl_3$-d_1): δ 7.41 (d, *J* = 8.5 Hz, 2H), 7.12-7.06 (m, 2H), 6.87 (d, *J* = 702 Hz, 1H), 5.09 (d, *J* = 9.5 Hz, 2H), 4.09-3.95 (m, 2H), 2.56 (t, *J* = 7.5 Hz, 3H), 1.74 (quint, *J* = 7.6 Hz, 2H), 1.65 (quint, *J* = 6.6 Hz, 2H), 1.44 (tq, *J* = 7.6, 7.5 Hz, 2H), 1.40-1.19 (m, 24H), 0.97 (t, *J* = 7.4 Hz, 3H), 0.88 (t, *J* = 7.0 Hz, 3H). **^{13}C-NMR** (151 MHz, $CDCl_3$-d_1): *δ* 172.14, 150.92, 133.16, 129.16, 121.90, 66.54 (d, *J* = 5.5 Hz), 66.02 (d, *J* = 6.2 Hz), 34.08, 30.34 (d, *J* = 6.2 Hz), 31.91, 29.69, 29.68, 29.66, 29.65, 29.63, 29.55, 29.48, 29.35, 29.09, 22.23, 26.95, 25.45, 22.68, 14.11, 13.71. **^{31}P-NMR** (243 MHz, $CDCl_3$-d_1): *δ* 7.73. **HRMS (ESI-TOF) m/z**: calculated for $C_{27}H_{51}NO_5P$ $[M+NH_4]^+$ 500.3499, found 500.3510. **IR**: ν [cm^{-1}] = 2958, 2915, 2847, 1751, 1511, 1471, 1462, 1249, 1218, 1203, 1170, 1141, 1098, 1065, 1032, 1010, 992, 957, 949, 925, 859, 848, 823, 727, 720, 551, 492, 420.

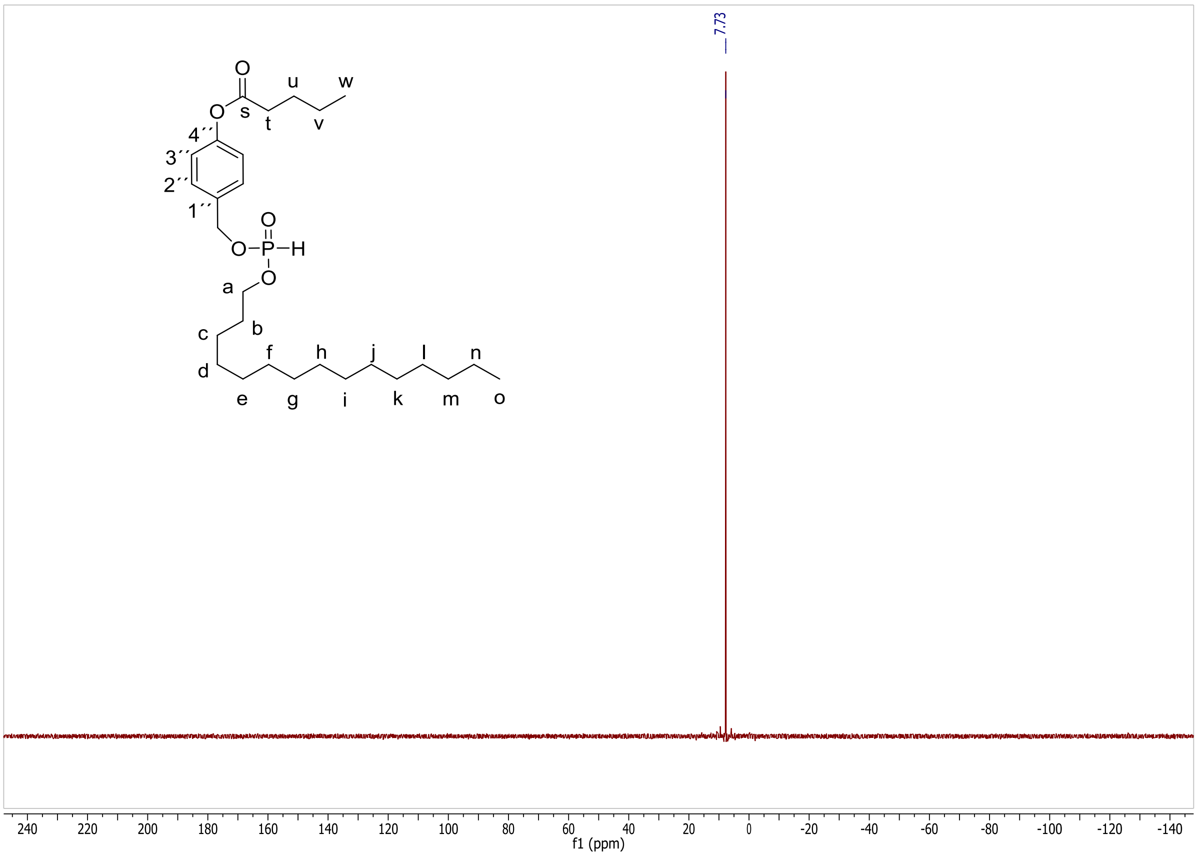

(AB-C_4H_9,alkyl-$C_{18}H_{37}$)-*H*-phosphonate 10cz

According to general procedure 1, with 0.23 mL diphenyl phosphonate (1.2 mmol, 1.2 equiv.) at 0 °C, 0.27 g 1-octadecanol **8z** (1.0 mmol, 1.0 equiv.) was added and following with 0.29 g 4-(hydroxymethyl)phenylpentanoate **9c** (1.4 mmol, 1.4 equiv.). Reaction time was 3 h at room temperature. Column chromatography (SiO_2, petrol ether/ethylacetate/CH_3COOH 8:2:0.005 v/v/v). Yield: 57%, 0.30 g, as white solid. **^{1}H-NMR** (600 MHz, $CDCl_3$-d_1): δ 7.43-7.36 (m, 2H), 7.17-7.04 (m, 2H), 6.87 (d, *J* = 702 Hz, 1H), 5.09 (d, *J* = 9.5 Hz, 2H), 4.10-3.95 (m, 2H), 2.56 (t, *J* = 7.5 Hz, 2H), 1.74 (quint, *J* = 7.5 Hz, 2H), 1.64 (quint, *J* = 7.2 Hz, 2H),1.44 (tq, *J* = 7.6, 7.5 Hz, 2H), 1.38-1.17 (m, 30H), 0.97 (t, *J* = 7.4 Hz, 3H), 0.87 (t, *J* = 7.0 Hz, 3H). **^{13}C-NMR** (151 MHz, $CDCl_3$-d_1): *δ* 172.1, 150.9, 133.19, 129.1, 121.9, 66.52 (d, *J* = 6.0 Hz), 65.98 (d, *J* = 6.0 Hz), 34.1, 30.33 (d, *J* = 6.0 Hz), 31.89, 30.35, 30.31, 29.67, 29.65, 29.63, 29.62, 29.53, 29.46, 29.33, 29.07, 26.93, 25.44, 22.67, 22.21, 14.09, 13.69. **^{31}P-NMR** (243 MHz, $CDCl_3$-d_1): *δ* 7.70. **HRMS (ESI-TOF) m/z**: calculated for $C_{30}H_{53}NaO_5P$ $[M+Na]^+$ 547.3523, found 547.3525. **IR**: ν [cm^{-1}] = 2955, 2918, 2872, 2848, 1753, 1509, 1460, 1414, 1382, 1352, 1248, 1234, 1220, 1204, 1171, 1147, 1106, 1086, 1023, 1003, 991, 971, 952, 930, 896, 876, 852, 822, 723, 552, 514, 454, 426, 415.

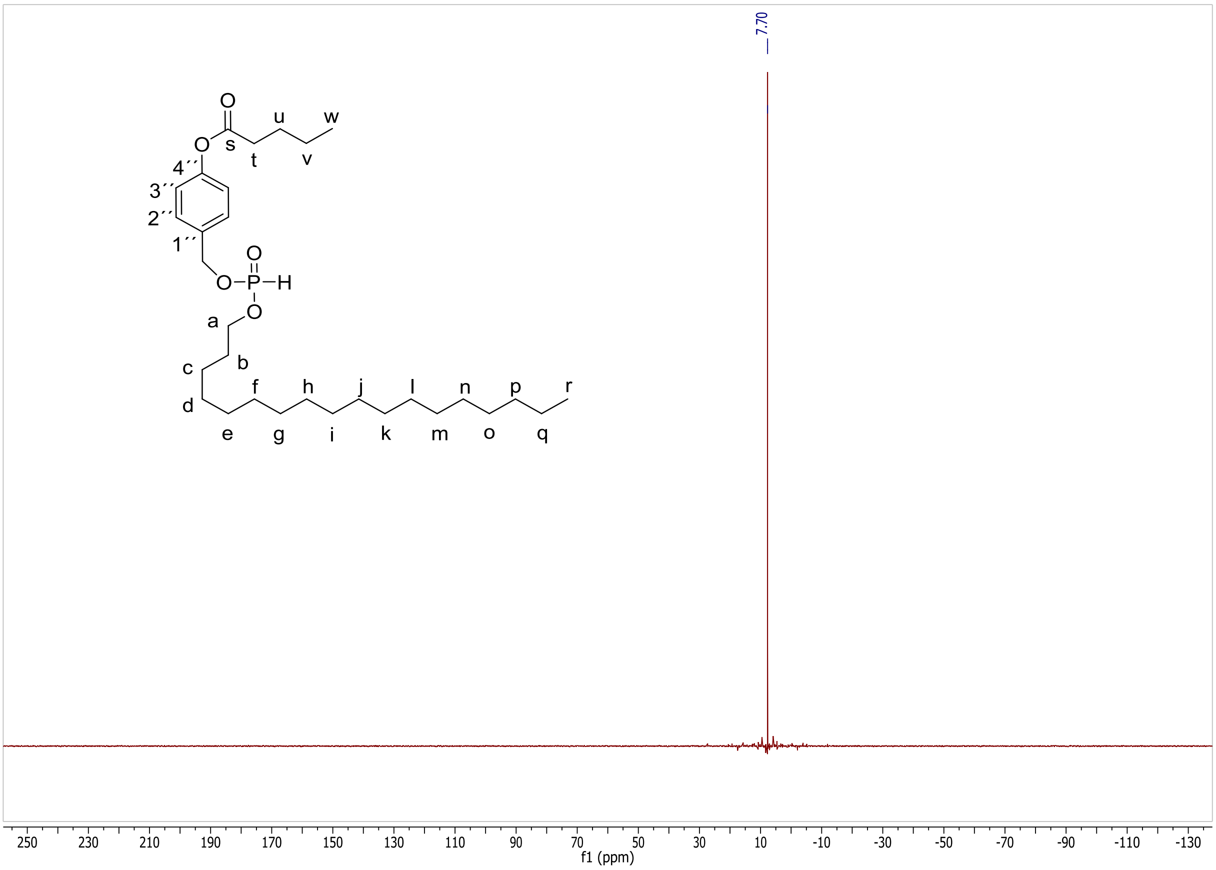

(AB-C_6H_{13},alkyl-$C_{15}H_{31}$)-*H*-phosphonate 10dy

According to general procedure 1, with 0.23 mL diphenyl phosphonate (1.2 mmol, 1.2 equiv.) at 0 °C, 0.23 g 1-pentadecanol **8y** (1.00 mmol, 1.0 equiv.) was added and following with 0.33 g 4-(hydroxymethyl)phenylheptanoate **9d** (1.4 mmol, 1.4 equiv.). Reaction time was 3 h at room temperature. Column chromatography (SiO_2, petrol ether/ethylacetate/CH_3COOH 7:3:0.005 v/v/v). Yield: 31%, 0.158 g, as white solid. **^{1}H-NMR** (600 MHz, $CDCl_3$-d_1): δ 7.42 (d, *J* = 8.4 Hz, 2H), 7.13-7.06 (m, 2H), 6.86 (d, *J* = 708 Hz, 1H), 5.09 (d, *J* = 9.6 Hz, 2H), 4.09-3.95 (m, 2H), 2.55 (t, *J* = 7.5 Hz, 3H), 1.75 (quint, *J* = 7.6 Hz, 2H), 1.64 (quint, *J* = 6.6 Hz, 2H), 1.45-1.36 (m, 2H), 1.36-1.18 (m, 28H), 0.91 (t, $^3J_{HH}$ = 7.0 Hz, 3H), 0.89 (t, $^3J_{HH}$ = 7.1 Hz, 3H). **^{13}C-NMR** (151 MHz, $CDCl_3$-d_1): *δ* 172.14, 150.91, 133.20, 129.15, 121.92, 66.53 (d, $^2J_{CP}$ = 5.5 Hz), 66.01 (d, $^2J_{CP}$ = 6.2 Hz), 34.35, 30.33 (d, $^3J_{CP}$ = 6.3 Hz), 31.90, 29.65, 29.64, 29.62, 29.53, 29.46, 29.34, 29.08, 22.67, 22.45, 28.74, 25.44, 24.82, 14.12, 14.01. **^{31}P-NMR** (243 MHz, $CDCl_3$-d_1): *δ* 7.72. **HRMS (ESI-TOF) m/z**: calculated for $C_{29}H_{55}NO_5P$ $[M+NH_4]^+$ 528.3812, found 528.3825. **IR**: ν [cm^{-1}] = 2955, 2916, 2871, 2849, 1753, 1509, 1466, 1386, 1291, 1236, 1218, 1167, 1153, 1104, 1076, 1039, 985, 947, 925, 866, 821, 721.

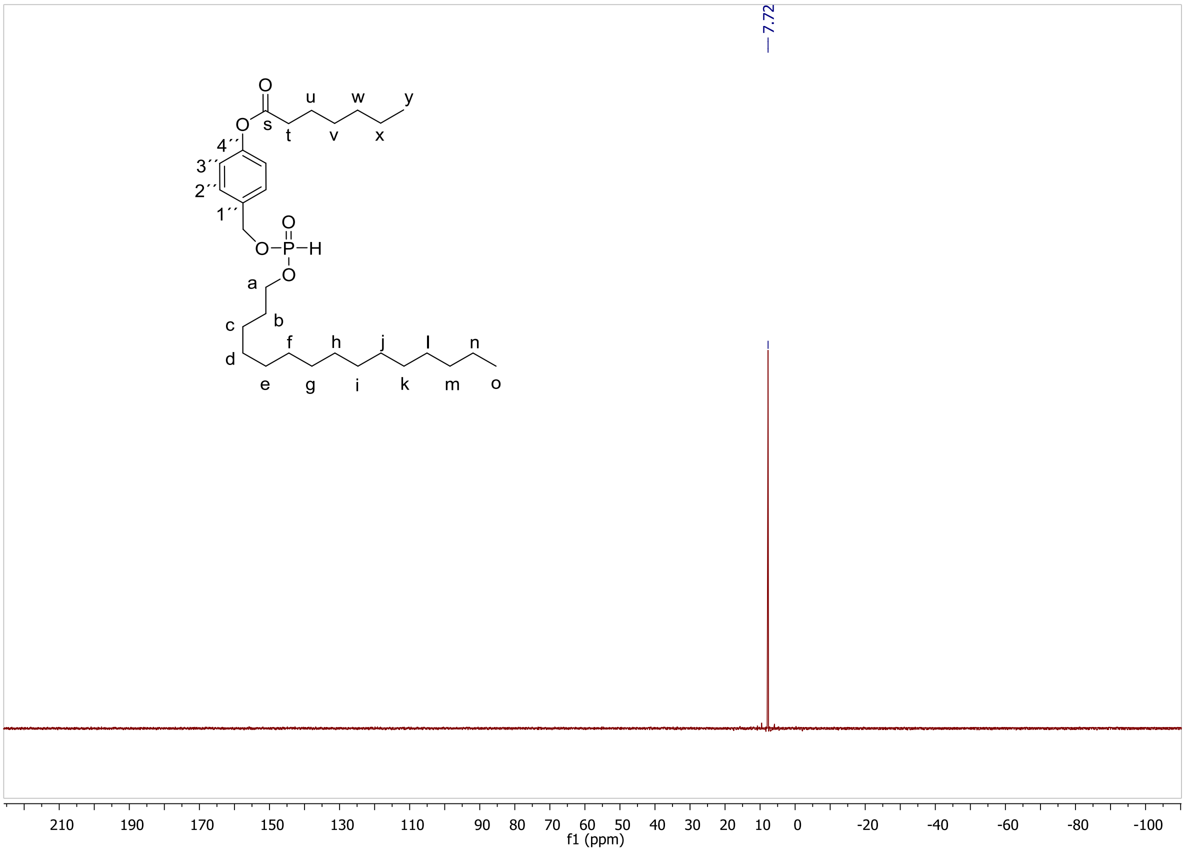

(AB-C_6H_{13},alkyl-$C_{18}H_{37}$)-*H*-phosphonate 10dz

According to general procedure 1, with 0.23 mL diphenyl phosphonate (1.2 mmol, 1.2 equiv.) at 0 °C, 0.27 g 1-octadecanol **8z** (1.0 mmol, 1.0 equiv.) was added and following with 0.33 g 4-(hydroxymethyl)phenylheptanoate **9d** (1.4 mmol, 1.4 equiv.). Reaction time was 3 h at room temperature. Column chromatography (SiO_2, petrol ether/ethylacetate/CH_3COOH 8:2:0.005 v/v/v). Yield: 63%, 0.35 g, as white solid. **^{1}H-NMR** (600 MHz, $CDCl_3$-d_1): δ 7.43-7.36 (m, 2H), 7.12-7.04 (m, 2H), 6.87 (d, *J* = 702 Hz, 1H), 5.09 (d, *J* = 9.6 Hz, 2H), 4.10-3.94 (m, 2H), 2.55 (t, *J* = 7.5 Hz, 2H), 1.75 (quint, *J* = 7.5 Hz, 2H), 1.64 (quint, *J* = 7.2 Hz, 2H),1.46-1.37 (m, 2H), 1.37-1.18 (m, 34H), 0.90 (t, *J* = 6.9 Hz, 3H), 0.87 (t, *J* = 7.0 Hz, 3H). **^{13}C-NMR** (151 MHz, $CDCl_3$-d_1): *δ* 172.1, 150.9, 133.2, 129.1, 121.9, 66.52 (d, *J* = 5.5 Hz), 65.99 (d, *J* = 6.2 Hz), 34.35, 30.33 (d, *J* = 6.3 Hz), 31.90, 31.40, 29.68, 29.65, 29.64, 29.62, 29.53, 29.46, 29.34, 29.08, 22.67, 22.45, 28.74, 25.44, 24.84, 14.09, 13.99. **^{31}P-NMR** (243 MHz, $CDCl_3$-d_1): *δ* 7.70. **HRMS (ESI-TOF) m/z**: calculated for $C_{32}H_{57}NaO_5P$ $[M+Na]^+$ 575.3836, found 575.3831. **IR**: ν [cm^{-1}] = 2956, 2919, 2870, 2847, 1754, 1509, 1460, 1384, 1249, 1235, 1219, 1204, 1171, 1146, 1110, 1086, 1023, 1003, 991, 971, 952, 927, 877, 853, 823, 724, 552, 514, 452, 428, 413.

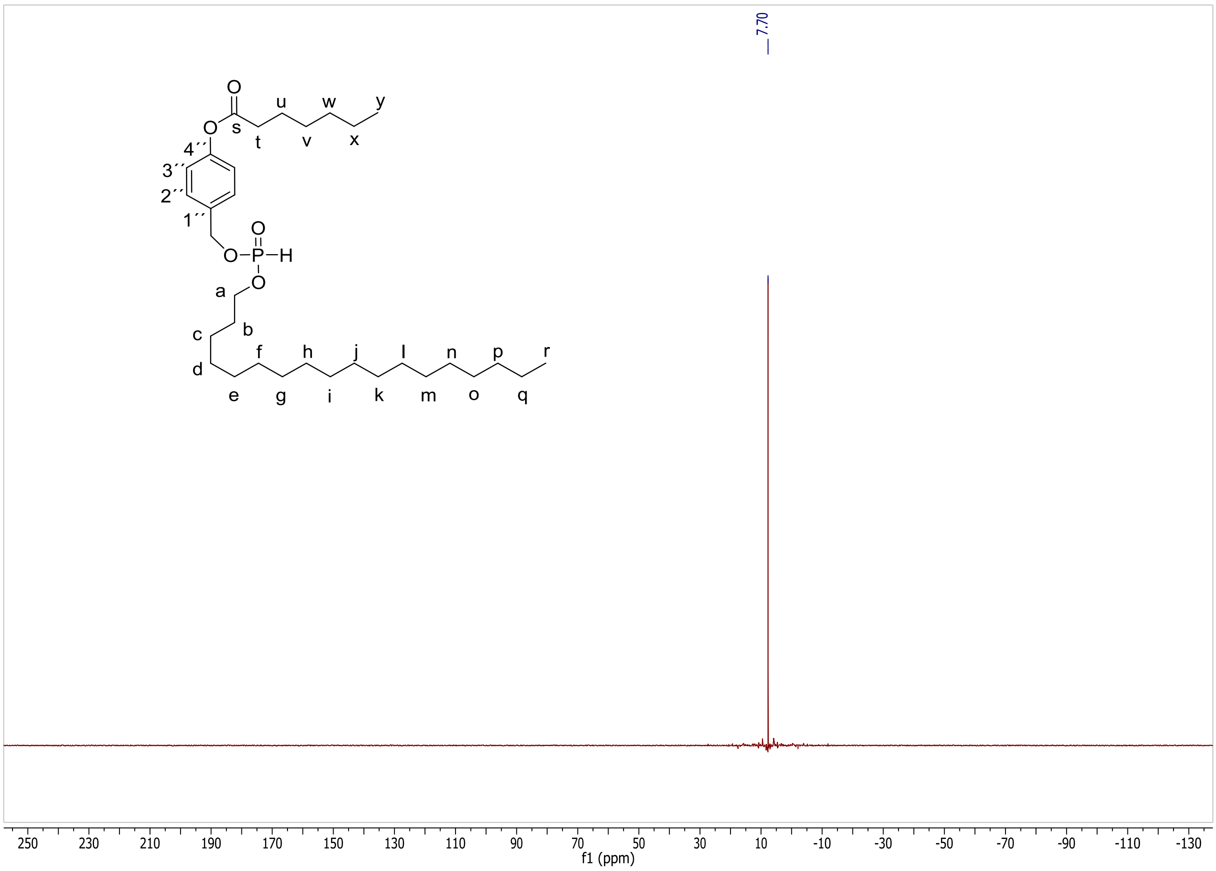

(AB-$C_{15}H_{31}$,alkyl-C_4H_9)-*H*-phosphonate 10ew

According to general procedure 1, with 0.23 mL diphenyl phosphonate (1.2 mmol, 1.2 equiv.) at 0 °C, 0.36 g 4-(hydroxymethyl)phenylhexadecanoate **9e** (1.0 mmol, 1.0 equiv.) was added and following with 0.10 g 1-butanol **8w** (1.4 mmol, 1.4 equiv.). Reaction time was 3 h at room temperature. Column chromatography (SiO_2, petrol ether/ethylacetate/CH_3COOH 8:2:0.005 v/v/v). Yield: 39%, 0.188 g, as white solid.^{1}**H-NMR** (400 MHz, $CDCl_3$-d_1): δ 7.41 (d, *J* = 8.5 Hz, 2H), 7.09 (d, *J* = 8.5 Hz, 2H), 6.87 (d, *J* = 704 Hz, 1H), 5.09 (d, *J* = 9.5 Hz, 2H), 4.12-3.93 (m, 2H), 2.55 (t, *J* = 7.5 Hz, 2H), 1.75 (quint, *J* = 7.4 Hz, 2H), 1.64 (quint, *J* = 8.4 Hz, 2H), 1.48-1.17 (m, 26H), 0.92 (t, *J* = 7.3 Hz, 3H), 0.88 (t, *J* = 7.0 Hz, 3H). ^{13}**C-NMR** (101 MHz, $CDCl_3$-d_1): *δ* 172.16, 150.94, 133.12, 129.17, 121.91, 66.58 (d, $^2J_{CP}$ = 5.6 Hz), 65.71 (d, $^2J_{CP}$ = 6.2 Hz), 34.38, 32.30 (d, $^3J_{CP}$ = 6.3 Hz), 31.91, 29.68, 29.67, 29.64, 29.59, 29.45, 29.35, 29.24, 29.09, 24.90, 22.68, 18.66, 14.11, 13.51. ^{31}**P-NMR** (162 MHz, $CDCl_3$-d_1): *δ* 7.72. **HRMS (ESI-TOF) m/z**: calculated for $C_{27}H_{47}NaO_5P$ $[M+Na]^+$ 505.3053, found 505.3054. **IR**: v [cm^{-1}] = 2956, 2914, 2872, 2848, 1748, 1606, 1510, 1464, 1411, 1385, 1348, 1330, 1308, 1285, 1262, 1241, 1221, 1167, 1150, 1072, 987, 925, 819, 770, 719, 693, 580, 524, 446, 416.

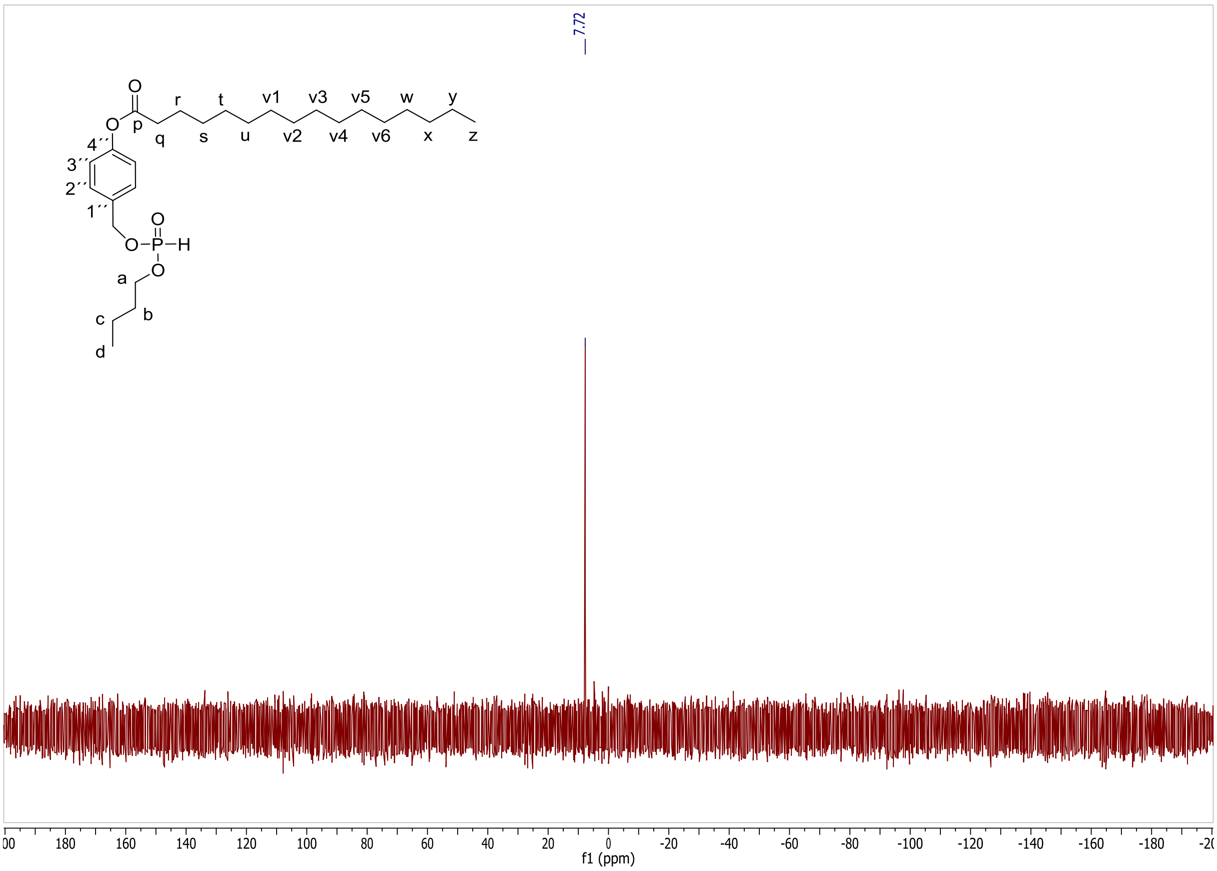

(AB-$C_{15}H_{31}$,alkyl-$C_{15}H_{31}$)-*H*-phosphonate 10ey

According to general procedure 1, with 0.23 mL diphenyl phosphonate (1.2 mmol, 1.2 equiv.) at 0 °C, 0.23 g 1-pentadecanol **8y** (1.0 mmol, 1.0 equiv.) was added and following with 0.51 g 4-(hydroxymethyl)phenylhexadecanoate **9e** (1.4 mmol, 1.4 equiv.). Reaction time was 3 h at room temperature. Column chromatography (SiO_2, petrol ether/ethylacetate/CH_3COOH 8:2:0.005 v/v/v). Yield: 46%, 0.293 g, as white solid. **^{1}H-NMR** (600 MHz, $CDCl_3$-d_1): δ 7.41 (d, *J* = 8.2 Hz, 2H), 7.15-7.05 (m, 2H), 6.87 (d, *J* = 702 Hz, 1H), 5.09 (d, *J* = 9.6 Hz, 2H), 4.10-3.93 (m, 2H), 2.55 (t, *J* = 7.5 Hz, 3H), 1.74 (quint, *J* = 7.5 Hz, 2H), 1.65 (quint, *J* = 6.9 Hz, 2H), 1.40 (quint, *J* = 6.9 Hz, 2H), 1.38-1.18 (m, 46H), 0.97 (t, *J* = 7.4 Hz, 3H), 0.81 (t, *J* = 7.0 Hz, 6H). **^{13}C-NMR** (151 MHz, $CDCl_3$-d_1): *δ* 172.14, 150.92, 133.16 (d, *J* = 6.1 Hz), 129.15, 121.90, 66.54 (d, *J* = 5.5 Hz), 66.02 (d, *J* = 6.2 Hz), 34.37, 30.33 (d, *J* = 6.3 Hz), 31.91, 29.68, 29.66, 29.64, 29.63, 29.59, 29.54, 29.47, 29.45, 29.35, 29.24, 29.09, 29.08, 22.68, 25.44, 24.90, 14.11. **^{31}P-NMR** (243 MHz, $CDCl_3$-d_1): *δ* 7.72. **HRMS (ESI-TOF) m/z**: calculated for $C_{38}H_{73}NO_5P$ $[M+NH_4]^+$ 654.5221, found 654.5215. **IR**: ν [cm^{-1}] = 2961, 2915, 2847, 1750, 1511, 1472, 1463, 1382, 1263, 1249, 1218, 1203, 1010, 992, 957, 949, 925, 859, 848, 823, 728, 720, 551, 492, 421.

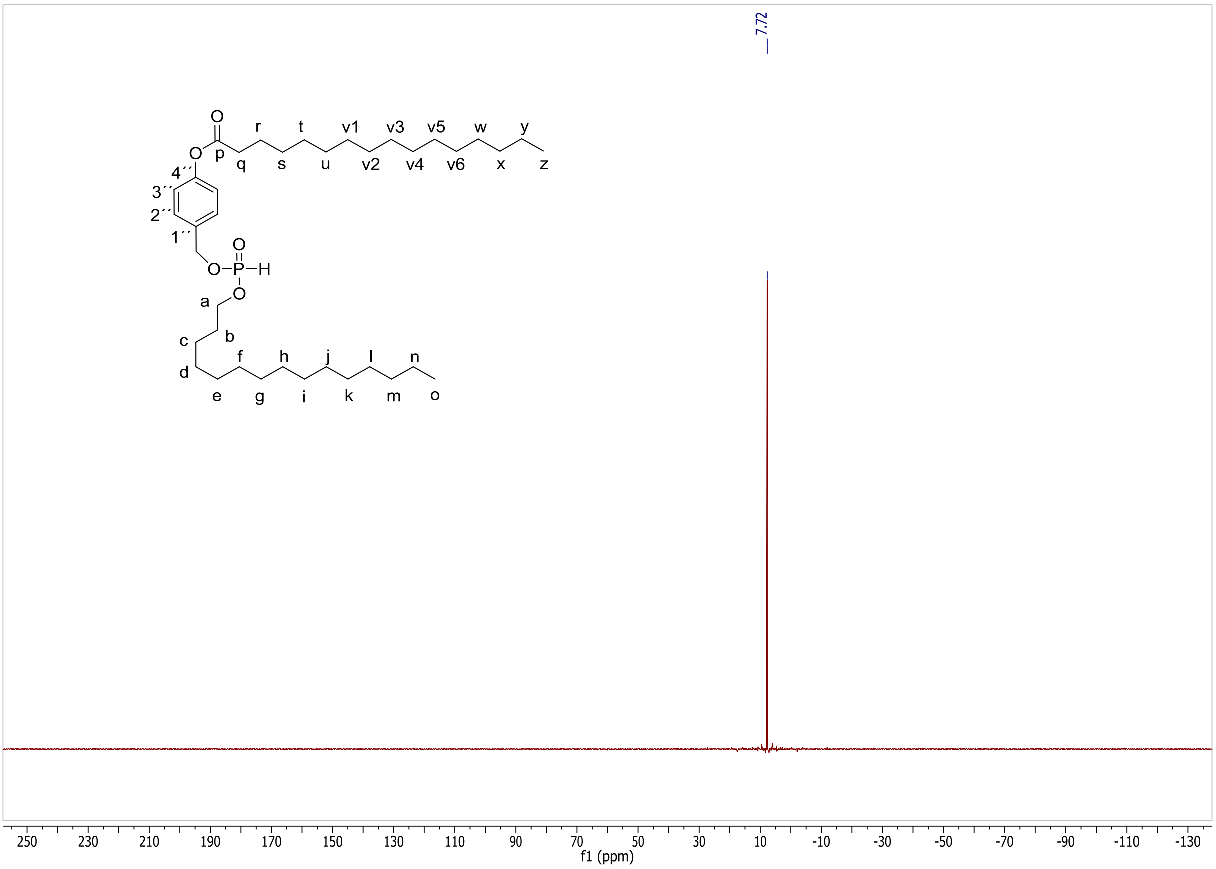

γ-(AB-CH$_3$,alkyl-C$_{18}$H$_{37}$)-d4TTP 6az

According to general procedure 2, the reactions were performed under dry conditions using 72 mg *H*-phosphonate **10az** (0.15 mmol, 1.0 equiv.) and 88.5 mg d4TMP 2×nBu$_4$N$^+$ salt (0.11 mmol, 0.75 equiv.). Yield: 47%, 48 mg, as white cotton. **^{1}H-NMR** (600 MHz, CD$_3$OD-d$_4$): *δ* 7.73 -7.68 (m, 1H), 7.52-7.48 (m, 2H), 7.13-7.09 (m, 2H), 6.96 (dt, *J* = 3.3, 1.6 Hz, 1H), 6.52 (dt, *J* = 6.0 Hz, *J* = 1.8 Hz, 1H), 5.85 (d, *J* = 6.1 Hz, 1H), 5.24-5.20 (m, 2H), 5.02-4.96 (m, 1H), 4.35-4.17 (m, 2H), 4.17-4.08 (m, 2H), 2.29 (s, 3H), 1.93 (d, *J* = 4.1 Hz, 3H), 1.65 (quint, *J* = 6.5 Hz, 2H), 1.40-1.23 (m, 30H**)**, 0.91 (t, *J* = 7.1 Hz, 3H). **^{13}C-NMR** (151 MHz, CD$_3$OD-d$_4$): *δ* 171.00, 166.60, 152.80, 152.32, 138.72, 135.84, 134.05, 130.35 (d, $^4J_{CP}$ = 4.4 Hz), 127.15, 122.88, 122.87, 112.09, 90.85, 87.26 (d, $^3J_{CP}$ = 7.7 Hz), 70.22 (d, $^2J_{CP}$ = 5.8 Hz), 69.84 (d, $^2J_{CP}$ = 6.2 Hz), 67.84 (d, $^2J_{CP}$ = 6.3 Hz), 33.08, 31.25 (d, $^3J_{CP}$ = 7.8 Hz), 30.80, 30.76, 30.73, 30.68, 30.48, 30, 26.56, 23.74, 20.93, 14.44, 12.49. **^{31}P-NMR** (243 MHz, CD$_3$OD-d$_4$): *δ* -11.76 (d, $^2J_{PP}$ = 17.9 Hz, **P-α**), -12.98 (d, $^2J_{PP}$ = 17.3 Hz, **P-γ**), -23.67 (t, $^2J_{PP}$ = 18.6 Hz, **P-β**). **HRMS (ESI-TOF) m/z**: calculated for C$_{37}$H$_{58}$N$_2$O$_{15}$P$_3$ [M-H]$^-$ 863.3056, found 863.3169. **IR**: ν [cm^{-1}] = 3037, 2921, 2851, 1762, 1690, 1508, 1456, 1368, 1248, 1216, 1195, 1167, 1126, 1080, 1007, 905, 837, 808, 783, 767, 720, 647, 484.

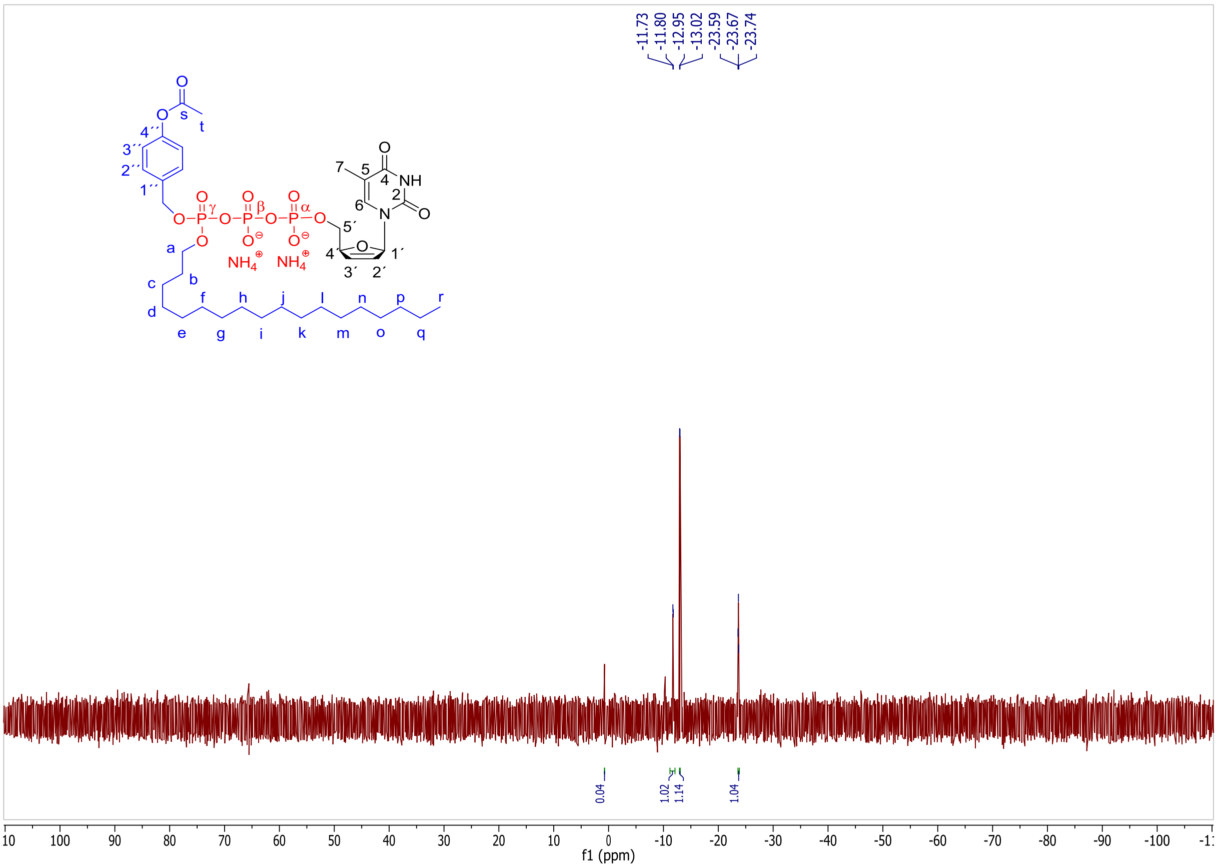

γ-(AB-C_2H_5,alkyl-$C_{18}H_{37}$)-d4TTP 6bz

According to general procedure 2, the reactions were performed under dry conditions using 75 mg *H*-phosphonate **10bz** (0.15 mmol, 1.0 equiv.) and 88 mg d4TMP 2×nBu$_4$N$^+$ salt (0.11 mmol, 0.75 equiv.). Yield: 63%, 64 mg, as white cotton. **^{1}H-NMR** (600 MHz, CD_3OD-d$_4$): *δ* 7.74-7.67 (m, 1H), 7.54-7.46 (m, 2H), 7.11 (d, *J* = 8.1 Hz, 2H), 6.96 (dt, *J* = 3.6, 1.7 Hz, 1H), 6.52 (dt, *J* = 5.9, 1.8 Hz, 1H), 5.85 (d, *J* = 6.0 Hz, 1H), 5.28-5.16 (m, 2H), 5.02-4.96 (m, 1H), 4.35-4.17 (m, 2H), 4.17-4.08 (m, 2H), 2.62 (q, *J* = 7.5 Hz, 2H), 1.93 (dd, *J* = 3.7, 1.3 Hz, 3H), 1.64 (quint, *J* = 7.2 Hz, 2H), 1.39-1.27 (m, 30H), 1.25 (t, *J* = 7.5 Hz, 3H), 0.92 (t, *J* = 7.0 Hz, 3H). **^{13}C-NMR** (151 MHz, CD_3OD-d$_4$): *δ* 174.45, 166.55, 152.79, 152.38, 138.67, 135.77, 135.14, 130.36 (d, *J* = 4.2 Hz), 127.18, 122.87, 122.86, 112.06, 90.85, 87.22 (d, *J* = 9.1 Hz), 70.24 (d, *J* = 5.7 Hz), 69.85 (d, *J* = 6.2 Hz), 67.85 (d, *J* = 5.8 Hz), 31.24 (d, *J* = 7.4 Hz), 33.07, 30.80, 30.76, 30.73, 30.68, 30.47, 30.30, 28.37, 26.56, 23.74, 14.46, 12.50, 9.32. **^{31}P-NMR** (243 MHz, CD_3OD-d$_4$): *δ* -11.84 (d, *J* = 19.7 Hz, **P-α**), -13.05 (d, *J* = 17.3 Hz, **P-γ**), -23.84 (s, **P-β**). **HRMS (ESI-TOF) m/z**: calculated for $C_{38}H_{60}N_2O_{15}P_3$ [M-H]$^-$ 877.3212, found 877.3171. **IR**: ν [cm^{-1}] = 2922, 2852, 1762, 1689, 1509, 1462, 1356, 1248, 1167, 1128, 1079, 1009, 904, 838, 806, 784, 768, 721, 697, 645, 576, 489, 401.

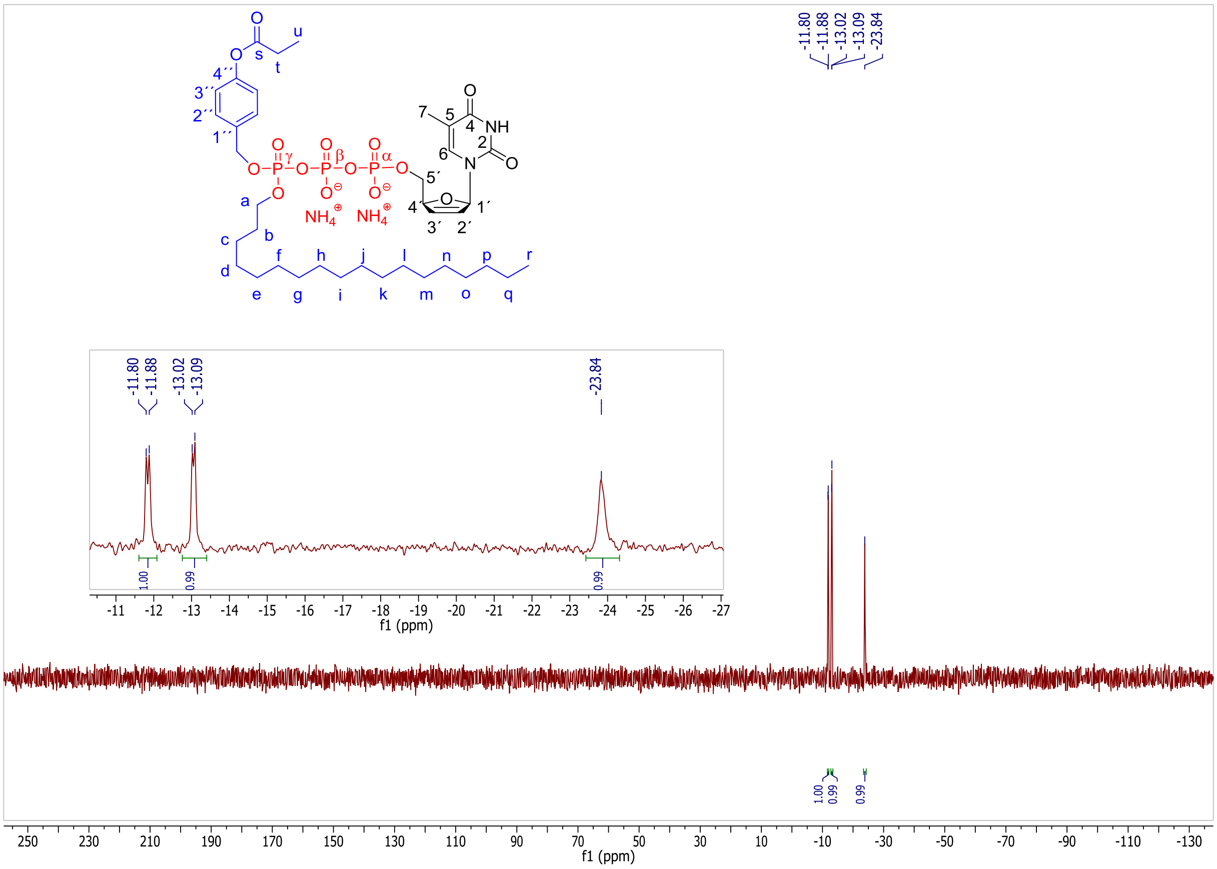

γ-(AB-C_4H_9,alkyl-C_4H_9)-d4TTP 6cw

According to general procedure 2, the reactions were performed under dry conditions using 49.2 mg *H*-phosphonate **10cw** (0.15 mmol, 1.0 equiv.) and 94.4 mg d4TMP 2×nBu_4N^+ salt (0.12 mmol, 0.80 equiv.). Yield: 59%, 53 mg, as white cotton. **^{1}H-NMR** (600 MHz, CD_3OD-d_4): δ 7.74-7.65 (m, 1H), 7.56-7.46 (m, 2H), 7.12-7.07 (m, 2H), 6.98-6.93 (m, 1H), 6.51 (dt, *J* = 5.9, 1.8 Hz, 1H), 5.85 (dt, *J* = 6.0, 1.7 Hz, 1H), 5.27-5.16 (m, 2H), 5.03-4.95 (m, 1H), 4.35-4.18 (m, 2H), 4.18-4.10 (m, 2H), 2.60 (t, *J* = 7.4 Hz, 2H), 1.92 (dd, *J* = 3.7, 1.2 Hz, 3H), 1.73 (quint, *J* = 7.5 Hz, 2H), 1.64 (quint, *J* = 6.7 Hz, 2H), 1.52-1.44 (m, 2H), 1.43-1.34 (m, 2H), 1.00 (t, *J* = 7.4 Hz, 3H), 0.92 (t, *J* = 7.4 Hz, 3H). **^{13}C-NMR** (151 MHz, CD_3OD-d_4): *δ* 173.82, 166.58, 152.81, 152.35, 138.67, 135.78, 135.15 (d, $^3J_{CP}$ = 7.6 Hz), 130.39 (d, $^4J_{CP}$ = 4.2 Hz), 127.15, 122.87, 112.06, 90.88, 87.22 (d, $^3J_{CP}$ = 9.2 Hz), 70.23 (d, $^2J_{CP}$ = 6.9 Hz), 69.52 (d, $^2J_{CP}$ = 6.3 Hz), 67.85 (d, $^2J_{CP}$ = 5.7 Hz), 34.74, 33.26 (d, $^3J_{CP}$ = 7.3 Hz), 28.05, 23.23, 19.68, 14.06, 13.92, 12.47. **^{31}P-NMR** (162 MHz, CD_3OD-d_4): *δ* -11.73 (d, $^2J_{PP}$ = 19.1 Hz, **P-α**), -12.97 (d, $^2J_{PP}$ = 16.8 Hz, **P-γ**), -23.60 (t, $^2J_{PP}$ = 18.0 Hz, **P-β**). **MALDI-MS** (m/z): calculated $C_{26}H_{37}N_2NaO_{15}P_3$ $[M+Na]^+$ 733.130, found 733.090; calculated $C_{26}H_{36}N_2O_{15}P_3$ $[M-H]^-$ 709.133, found 709.198. **IR**: ν [cm^{-1}] = 3167, 3030, 2961, 2873, 1757, 1689, 1453, 1245, 1201, 1168, 1126, 1080, 1008, 903, 835, 805, 783, 721, 695, 642, 520, 480, 452, 430, 423, 416, 402.

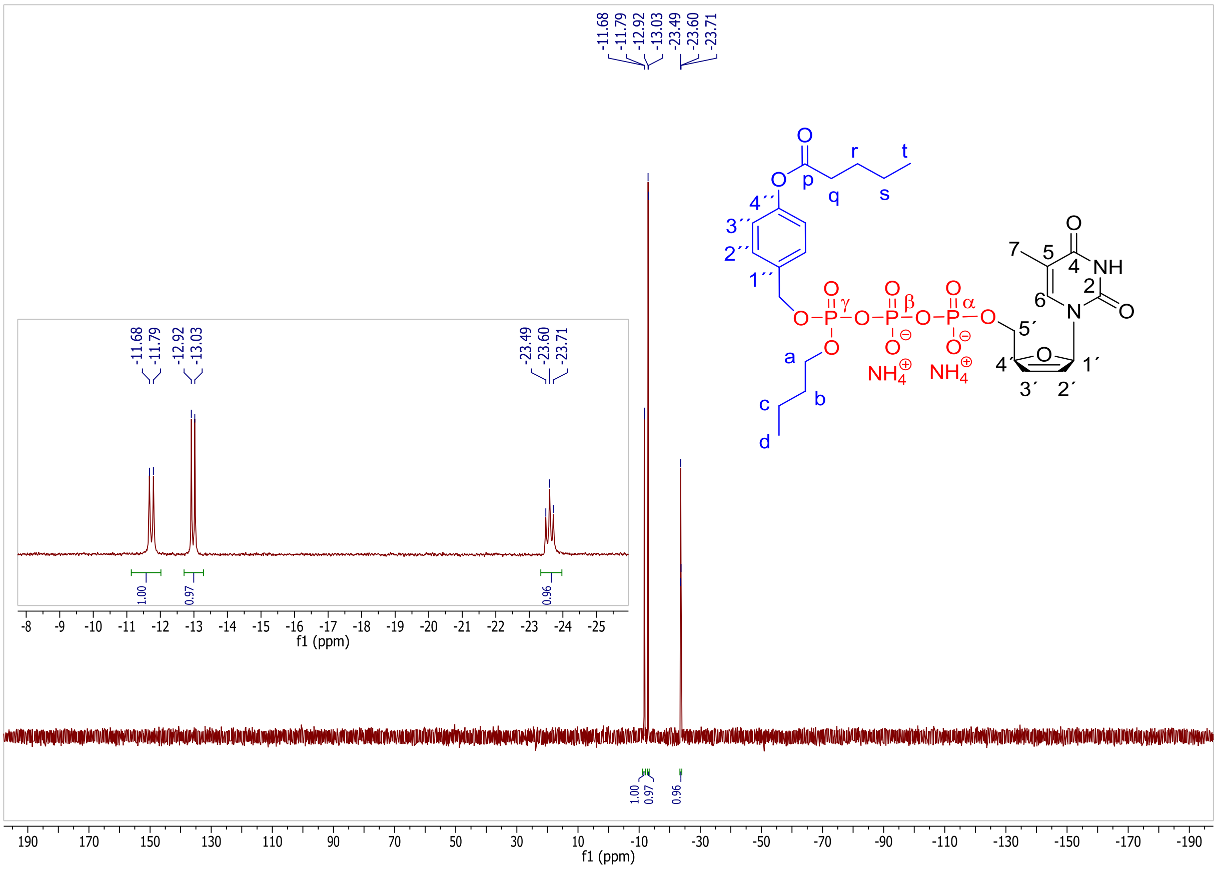

γ-(AB-C_4H_9,alkyl-$C_{15}H_{31}$)-d4TTP 6cy

According to general procedure 2, the reactions were performed under dry conditions using 72 mg *H*-phosphonate **10cy** (0.15 mmol, 1.0 equiv.) and 118 mg d4TMP 2×nBu_4N^+ salt (0.15 mmol, 1.0 equiv.). Yield: 33%, 44 mg, as white cotton. **^{1}H-NMR** (600 MHz, CD_3OD-d_4): *δ* 7.73 -7.66 (m, 1H), 7.50 (d, *J* = 8.7 Hz, 2H), 7.13-7.07 (m, 2H), 6.99-6.93 (m, 1H), 6.56-6.49 (m, 1H), 5.85 (dt, *J* = 6.1, 2.4 Hz, 1H), 5.26-5.16 (m, 2H), 4.99 (s, 1H), 4.36-4.17 (m, 2H), 4.17-4.07 (m, 2H), 2.60 (t, *J* = 7.4 Hz, 2H), 1.92 (d, *J* = 3.3 Hz, 3H), 1.74 (quint, *J* = 7.4 Hz, 2H), 1.64 (quint, *J* = 6.5 Hz, 2H), 1.48 (tq, *J* = 8.6, 8.0 Hz, 2H), 1.38-1.23 (m, 24H), 1.00 (t, *J* = 8.0 Hz, 3H), 0.92 (t, *J* = 6.4 Hz, 3H). **^{13}C-NMR** (151 MHz, CD_3OD-d_4): *δ* 173.73, 166.59, 152.79, 152.34, 138.69, 135.78, 135.17 (d, *J* = 3.5 Hz), 130.39 (d, *J* = 4.2 Hz), 127.18, 122.88, 122.87, 112.07, 90.85, 87.21 (d, *J* = 9.0 Hz), 70.25 (d, *J* = 5.5 Hz), 69.85 (d, *J* = 6.3 Hz), 67.87 (d, *J* = 5.7 Hz), 34.76, 33.08, 31.23 (d, *J* = 7.2 Hz), 30.82, 30.80, 30.78, 30.73, 30.68, 30.49, 30.30, 28.07, 26.55, 23.74, 23.25, 14.45, 14.10, 12.49. **^{31}P-NMR** (243 MHz, CD_3OD-d_4): *δ* -11.81 (d, *J* = 18.7 Hz, **P-α**), -12.98 (d, *J* = 16.7 Hz, **P-γ**), -23.71 (t, *J* = 17.3 Hz, **P-β**). **HRMS (ESI-TOF) m/z**: calculated for $C_{37}H_{58}N_2O_{15}P_3$ [M-H]$^-$ 863.3056, found 863.2956. **IR**: ν [cm^{-1}] = 3182, 3040, 2957, 2923, 2853, 1760, 1690, 1509, 1463, 1248, 1202, 1167, 1128, 1082, 1010, 906, 838, 807, 783, 768, 721, 696, 644, 485, 425, 401.

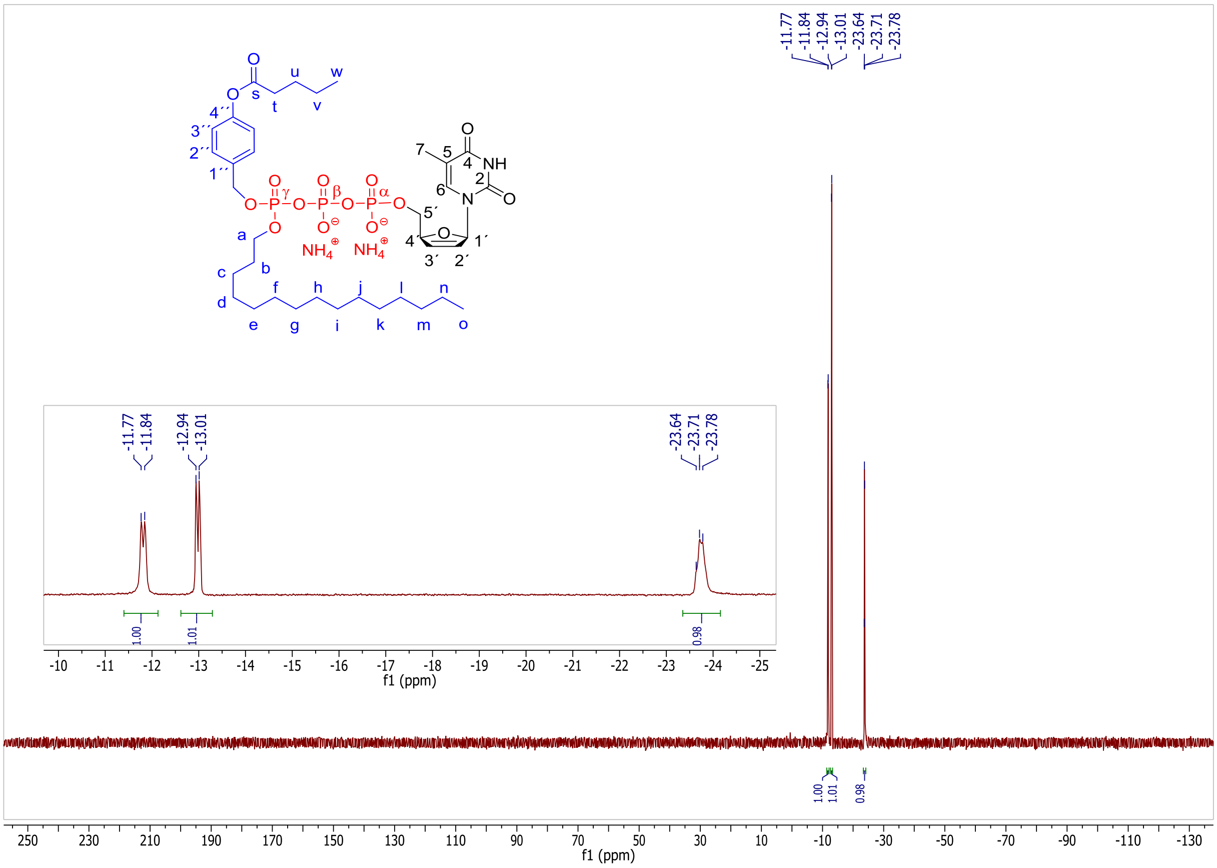

γ-(AB-C_4H_9,alkyl-$C_{18}H_{37}$)-d4TTP 6cz

According to general procedure 2, the reactions were performed under dry conditions using 79 mg *H*-phosphonate **10cz** (0.15 mmol, 1.0 eq.) and 118 mg d4TMP 2×nBu_4N^+ salt (0.15 mmol, 1.0 eq.). Yield: 30%, 42 mg, as white cotton. **^{1}H-NMR** (600 MHz, CD_3OD-d_4): *δ* 7.75-7.67 (m, 1H), 7.50 (dd, *J* = 8.7, 2.5 Hz, 2H), 7.10 (d, *J* = 8.3 Hz, 2H), 6.96 (dt, *J* = 3.6, 1.8 Hz, 1H), 6.56-6.48 (m, 1H), 5.88-5.83 (m, 1H), 5.28-5.16 (m, 2H), 5.02-4.96 (m, 1H), 4.35-4.17 (m, 2H), 4.17-4.06 (m, 2H), 2.60 (t, *J* = 7.4 Hz, 2H), 1.93 (dd, *J* = 3.8, 1.2 Hz, 3H), 1.73 (quint, *J* = 7.6 Hz, 2H), 1.64 (quint, *J* = 7.3 Hz, 2H), 1.48 (tq, *J* = 7.7, 7.4 Hz, 2H), 1.38-1.24 (m, 30H), 1.01 (t, *J* = 7.4 Hz, 3H), 0.92 (t, *J* = 7.0 Hz, 3H). **^{13}C-NMR** (151 MHz, CD_3OD-d_4): *δ* 173.74, 166.56, 152.79, 152.35, 138.68, 135.83, 135.16 (dd, *J* = 7.3, 3.5 Hz), 130.38 (d, *J* = 4.2 Hz), 127.16, 122.87, 122.86, 112.08, 90.86, 87.23 (d, *J* = 9.0 Hz), 70.25 (d, *J* = 6.0 Hz), 69.84 (d, *J* = 6.3 Hz), 67.85 (d, *J* = 5.8 Hz), 34.77, 31.24 (d, *J* = 7.1 Hz), 33.07, 30.80, 30.75, 30.73, 30.67, 30.47, 30.29, 28.07, 26.55, 23.73, 23.25, 14.45, 14.10, 12.49. **^{31}P-NMR** (243 MHz, CD_3OD-d_4): *δ* -11.76 (d, *J* = 18.0 Hz, **P-α**), -12.96 (d, *J* = 16.9 Hz, **P-γ**), -23.61--23.70 (m, **P-β**). **HRMS (ESI-TOF) m/z**: calculated for $C_{40}H_{64}N_2O_{15}P_3$ [M-H]$^-$ 905.3525, found 905.3530. **IR**: ν [cm^{-1}] = 2957, 2922, 2852, 1760, 1690, 1509, 1465, 1249, 1167, 1128, 1082, 1010, 908, 838, 807, 784, 768, 721, 696, 643, 576, 491, 422.

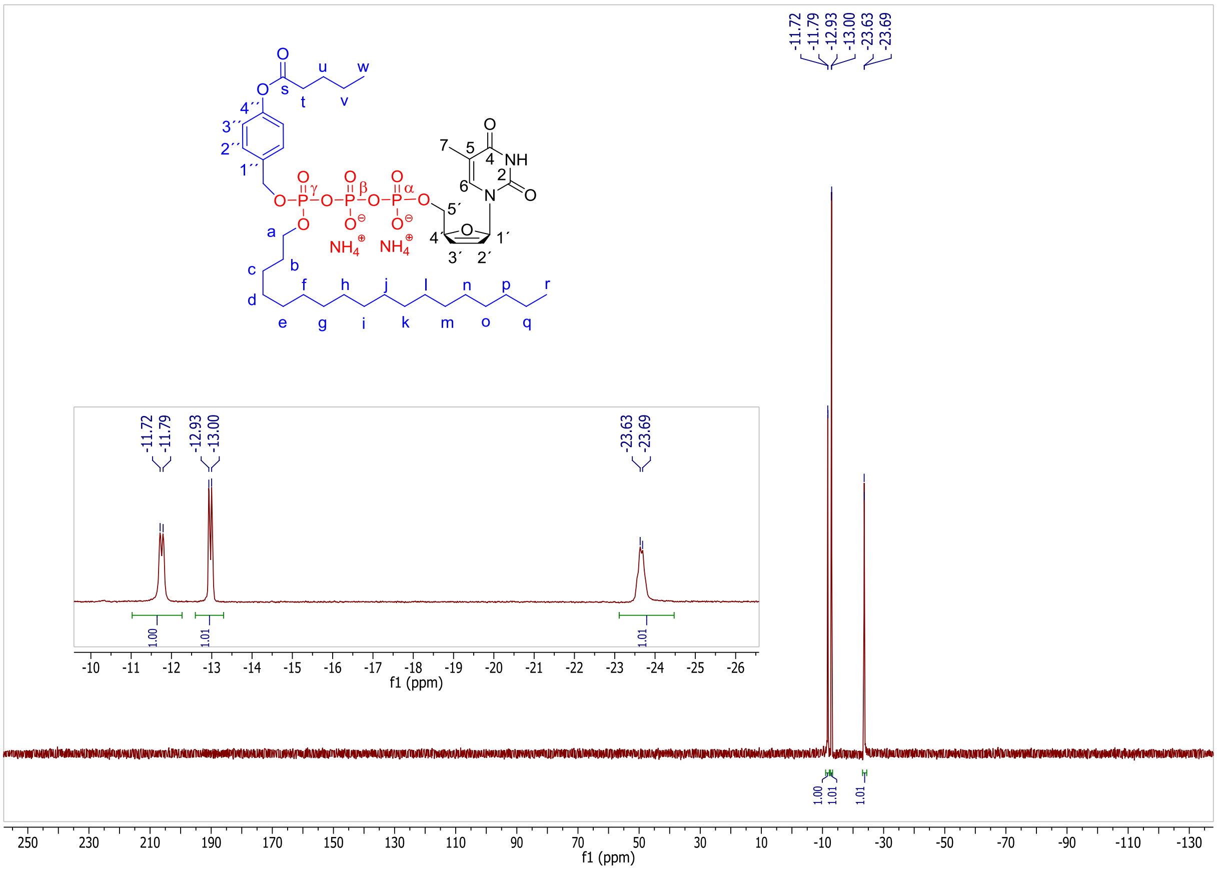

γ-(AB-C_6H_{13},alkyl-$C_{15}H_{31}$)-d4TTP 6dy

According to general procedure 2, the reactions were performed under dry conditions using 57 mg *H*-phosphonate **10dy** (0.11 mmol, 1.0 equiv.) and 66 mg d4TMP 2×nBu$_4$N$^+$ salt (0.08 mmol, 0.75 equiv.). Yield: 59%, 46 mg, as white cotton. ^{1}**H-NMR** (600 MHz, CD$_3$OD-d$_4$): *δ* 7.73-7.65 (m, 1H), 7.50 (dd, *J* = 8.7, 2.3 Hz, 2H), 7.10 (d, *J* = 8.3 Hz, 2H), 6.98-6.93 (m, 1H), 6.52 (dt, *J* = 6.0, 1.7 Hz, 1H), 5.85 (dt, *J* = 6.1, 2.5 Hz, 1H), 5.26-5.16 (m, 2H), 5.02-4.96 (m, 1H), 4.33-4.17 (m, 2H), 4.17-4.07 (m, 2H), 2.59 (t, *J* = 7.4 Hz, 2H), 1.93 (dd, *J* = 3.5, 1.2 Hz, 3H), 1.75 (quint, *J* = 7.4 Hz, 2H), 1.64 (quint, *J* = 6.7 Hz, 2H),1.52-1.42 (m, 2H), 1.42-1.22 (m, 28H), 0.95 (t, *J* = 7.1 Hz, 3H), 0.92 (t, *J* = 7.0 Hz, 3H). ^{13}**C-NMR** (151 MHz, CD$_3$OD-d$_4$): *δ* 173.74, 166.56, 152.81, 152.37, 138.63, 135.81, 135.19 (dd, *J* = 7.2, 2.7 Hz), 130.37 (d, *J* = 3.7 Hz), 127.15, 122.84, 122.83, 112.10, 90.93, 87.23 (d, *J* = 9.1 Hz), 70.25 (d, *J* = 7.4 Hz), 69.86 (d, *J* = 6.4 Hz), 67.90 (d, *J* = 5.9 Hz), 35.08, 33.05, 31.23 (d, *J* = 7.3 Hz), 30.79, 30.77, 30.74, 30.70, 30.64, 30.45, 30.27, 32.62, 29.84, 26.54, 25.94, 23.70, 23.54, 14.40, 14.34, 12.45. ^{31}**P-NMR** (243 MHz, CD$_3$OD-d$_4$): *δ* -11.72 (d, *J* = 18.9 Hz, **P-α**), -12.94 (d, *J* = 16.8 Hz, **P-γ**), -23.62 (t, *J* = 17.7 Hz, **P-β**). **HRMS (ESI-TOF) m/z**: calculated for $C_{39}H_{62}N_2O_{15}P_3$ [M-H]$^-$891.3369, found 891.3350. **IR**: ν [cm^{-1}] = 3181, 2956, 2921, 2852, 1759, 1690, 1509, 1466, 1379, 1250, 1167, 1128, 1113, 1083, 1009, 910, 838, 807, 784, 768, 722, 696, 644, 492, 423, 403.

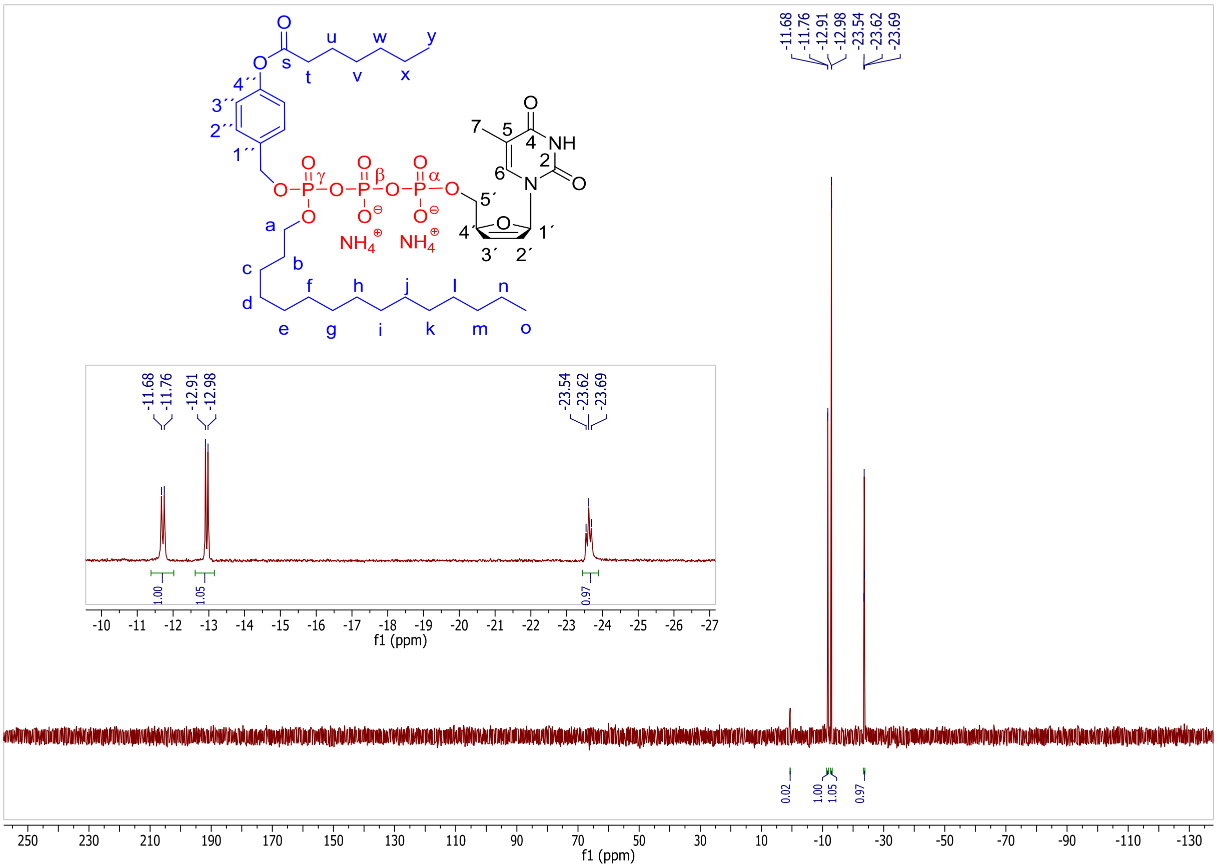

γ-(AB-C_6H_{13},alkyl-$C_{18}H_{37}$)-d4TTP 6dz

According to general procedure 2, the reactions were performed under dry conditions using 83 mg *H*-phosphonate **10dz** (0.15 mmol, 1.0 equiv.) and 118 mg d4TMP 2×nBu$_4$N$^+$ salt (0.15 mmol, 1.0 equiv.). Yield: 36%, 52 mg, as white cotton. **^{1}H-NMR** (600 MHz, CD_3OD-d$_4$): *δ* 7.73-7.63 (m, 1H), 7.47 (dd, *J* = 8.6, 2.6 Hz, 2H), 7.07 (d, *J* = 8.1 Hz, 2H), 6.93 (dt, *J* = 3.3, 1.5 Hz, 1H), 6.49 (dt, *J* = 5.9, 1.8 Hz, 1H), 5.82 (dt, *J* = 6.1, 1.8 Hz, 1H), 5.25-5.13 (m, 2H), 4.99-4.93 (m, 1H), 4.32-4.15 (m, 2H), 4.15-4.03 (m, 2H), 2.57 (t, *J* = 7.4 Hz, 2H), 1.90 (d, *J* = 3.8 Hz, 3H), 1.72 (quint, *J* = 7.5 Hz, 2H), 1.60 (quint, *J* = 6.9 Hz, 2H),1.46-1.39 (m, 2H), 1.39-1.19 (m, 34H), 0.92 (t, *J* = 6.9 Hz, 3H), 0.89 (t, *J* = 7.0 Hz, 3H). **^{13}C-NMR** (151 MHz, CD_3OD-d$_4$): *δ* 173.72, 166.58, 152.79, 152.35, 138.69, 135.80, 135.15 (dd, *J* = 7.2, 3.6 Hz), 130.40 (d, *J* = 4.2 Hz), 127.16, 122.87, 122.86, 112.07, 90.86, 87.23 (d, *J* = 9.1 Hz), 70.25 (d, *J* = 7.3 Hz), 69.84 (d, *J* = 6.3 Hz), 67.85 (d, *J* = 5.7 Hz), 35.05, 31.23 (d, *J* = 7.3 Hz), 33.08, 30.82, 30.81, 30.77, 30.74, 30.68, 30.48, 30.30, 32.67, 29.87, 26.55, 25.94, 23.74, 23.59, 14.46, 14.40, 12.50. **^{31}P-NMR** (243 MHz, CD_3OD-d$_4$): *δ* -11.75 (d, *J* = 19.0 Hz, **P-α**), -12.94 (d, *J* = 17.0 Hz, **P-γ**), -23.63 (t, *J* = 17.9 Hz, **P-β**). **HRMS (ESI-TOF) m/z**: calculated for $C_{42}H_{68}N_2O_{15}P_3$ [M-H]$^-$933.3838, found 933.3800. **IR**: ν [cm^{-1}] = 3184, 2956, 2921, 2852, 1759, 1691, 1509, 1466, 1379, 1250, 1168, 1127, 1113, 1082, 1010, 911, 839, 807, 784, 767, 722, 696, 644, 576, 492, 425, 400.

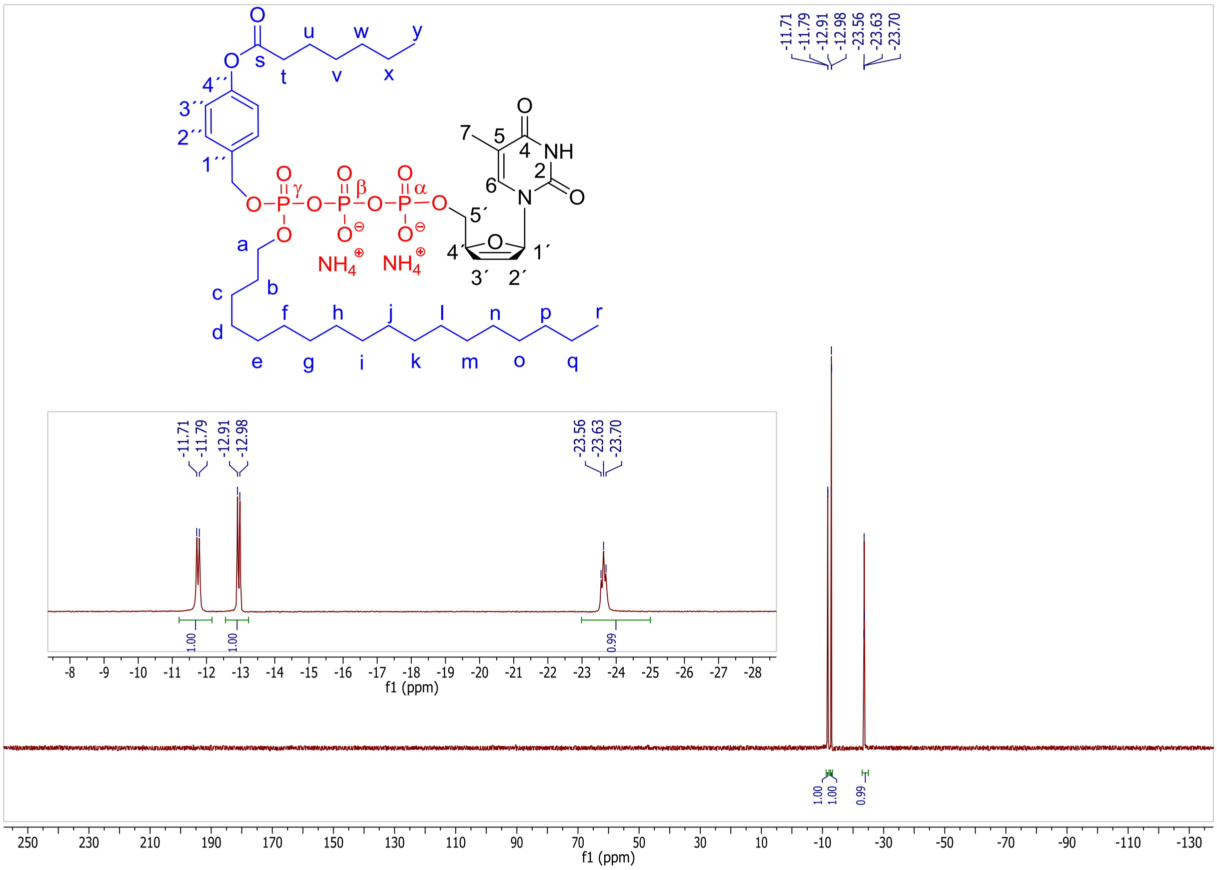

γ-(AB-$C_{15}H_{31}$,alkyl-C_4H_9)-d4TTP 6ew

According to general procedure 2, the reactions were performed under dry conditions using 73 mg *H*-phosphonate **10ew** (0.15 mmol, 1.0 equiv.) and 88 mg d4TMP 2×nBu$_4$N$^+$ salt (0.11 mmol, 0.75 equiv.). Yield: 33%, 29 mg, as white cotton. **^{1}H-NMR** (600 MHz, CD$_3$OD-d$_4$): *δ* 7.75-7.68 (m, 1H), 7.53-7.47 (m, 2H), 7.12-7.07 (m, 2H), 6.98-6.94 (m, 1H), 6.53 (dt, *J* = 6.0, 1.8 Hz, 1H), 5.85 (dt, *J* = 6.0, 1.4 Hz, 1H), 5.28-5.17 (m, 2H), 5.03-4.96 (m, 1H), 4.37-4.18 (m, 2H), 4.17-4.11 (m, 2H), 2.60 (t, *J* = 7.4 Hz, 2H), 1.93 (dd, *J* = 3.9, 1.2 Hz, 3H), 1.75 (quint, *J* = 7.4 Hz, 2H), 1.64 (quint, *J* = 6.6 Hz, 2H), 1.49-1.23 (m, 26H), 0.95-0.89 (m, 6H). **^{13}C-NMR** (151 MHz, CD$_3$OD-d$_4$): *δ* 173.81, 166.60, 152.81, 152.34, 138.74, 135.87, 135.22 (d, *J* = 3.1 Hz) 130.40 (d, *J* = 4.3 Hz), 127.12, 122.86, 112.08, 90.85, 87.27 (d, *J* = 9.2 Hz), 70.23 (d, *J* = 4.1 Hz), 69.51 (d, *J* = 6.3 Hz), 67.85 (d, *J* = 5.7 Hz), 35.02, 33.28 (d, *J* = 7.6 Hz), 33.08, 30.79, 30.78, 30.76, 30.71, 30.60, 30.48, 30.40, 30.16, 25.96, 23.74, 19.70, 14.44, 13.95, 12.48. **^{31}P-NMR** (243 MHz, CD$_3$OD-d$_4$): *δ* -11.81 (d, *J* = 19.4 Hz, **P-α**), -13.02 (d, *J* = 16.9 Hz, **P-γ**), -23.76 (t, *J* = 18.3 Hz, **P-β**). **HRMS (ESI-TOF) m/z**: calculated for $C_{37}H_{58}N_2O_{15}P_3$ [M-H]$^-$ 863.3056, found 863.3045. **IR**: ν [cm^{-1}] = 3184, 3049, 2958, 2850, 1759, 1692, 1508, 1465, 1249, 1167, 1127, 1082, 1012, 908, 838, 784, 768, 720, 646, 517, 492, 419, 401.

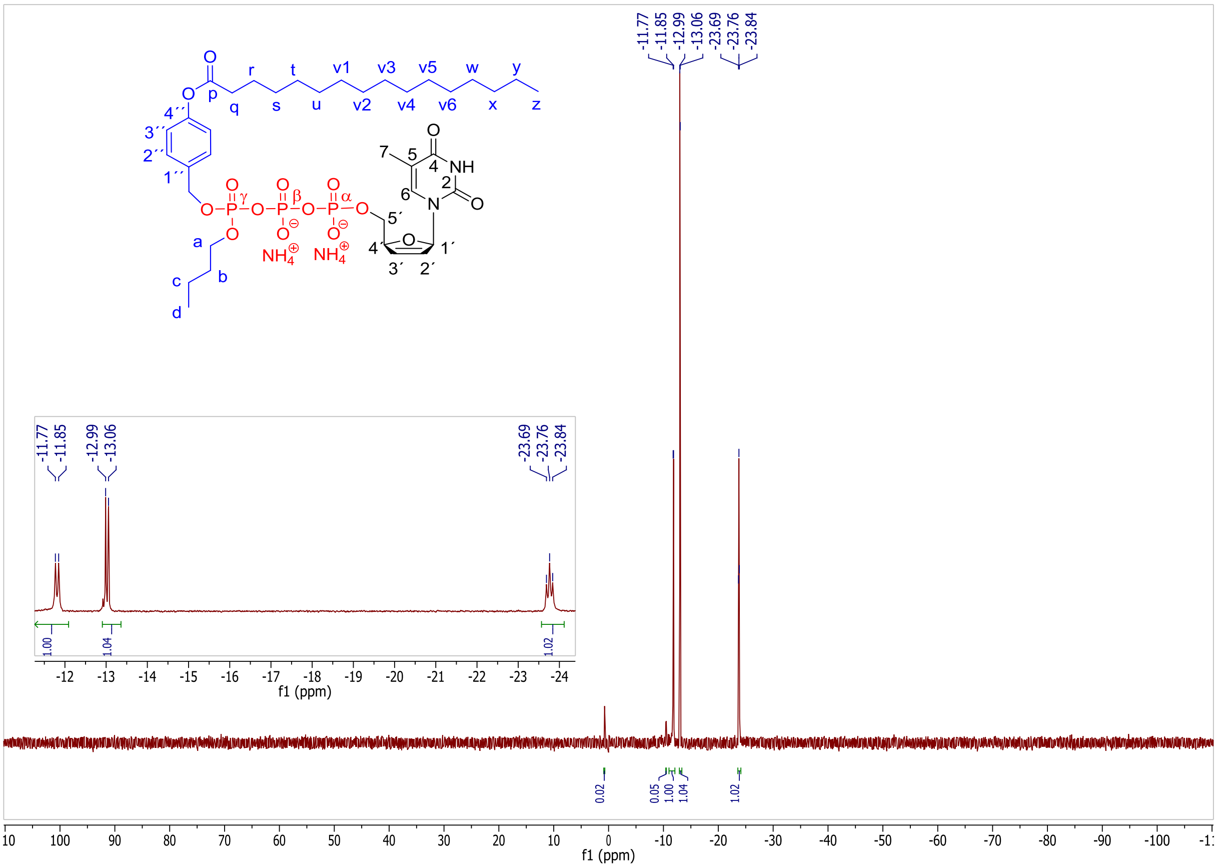

γ-(AB-$C_{15}H_{31}$,alkyl-$C_{15}H_{31}$)-d4TTP 6ey

According to general procedure 2, the reactions were performed under dry conditions using 97 mg *H*-phosphonate **10ey** (0.15 mmol, 1.0 equiv.) and 89 mg d4TMP 2×nBu_4N^+ salt (0.11 mmol, 0.75 equiv.). Yield: 44%, 52 mg, as white cotton. **^{1}H-NMR** (600 MHz, CD_3OD-d_4): *δ* 7.71 (d, $^4J_{HH}$ = 2.7 Hz, 1H), 7.56-7.45 (m, 2H), 7.15-7.06 (m, 2H), 6.98-6.94 (m, 1H), 6.53 (dt, *J* = 6.0 Hz, *J* = 1.7 Hz, 1H), 5.85 (dt, *J* = 5.8, 1.8 Hz, 1H), 5.29-5.17 (m, 2H), 5.01-4.97 (m, 1H), 4.34-4.17 (m, 2H), 4.16-4.06 (m, 2H), 2.59 (t, *J* = 7.4 Hz, 2H), 1.93 (d, *J* = 3.8 Hz, 3H), 1.75 (quint, *J* = 7.5 Hz, 2H), 1.62 (quint, *J* = 7.2 Hz, 2H),1.49-1.42 (m, 2H), 1.39-1.19 (m, 46H), 0.92 (t, *J* = 7.0 Hz, 6H). **^{13}C-NMR** (151 MHz, CD_3OD-d_4): *δ* 173.72, 166.57,152.80, 152.35, 138.70, 135.83, 135.15 (dd, $^3J_{CP}$ = 7.1, 3.6 Hz), 130.42 (d, $^4J_{CP}$ = 4.1 Hz), 127.16, 122.87, 122.86, 112.09, 90.85, 87.24 (d, $^3J_{CP}$ = 9.0 Hz), 70.27 (d, $^2J_{CP}$ = 7.5 Hz), 69.83 (d, $^2J_{CP}$ = 6.2 Hz), 67.85 (d, $^2J_{CP}$ = 5.7 Hz), 35.05, 33.10, 33.08, 31.23 (d, $^3J_{CP}$ = 7.3 Hz), 30.85, 30.83, 30.80, 30.78, 30.76, 30.72, 30.69, 30.61, 30.52, 30.48, 30.42, 30.31, 30.18, 26.56, 25.98, 23.76, 23.74, 14.47, 14.45, 12.49. **^{31}P-NMR** (243 MHz, CD_3OD-d_4): *δ* -11.76 (d, $^2J_{PP}$ = 19.2 Hz, **P-α**), -12.92 (d, $^2J_{PP}$ = 16.6 Hz, **P-γ**), -23.66 (t, $^2J_{PP}$ = 18.0 Hz, **P-β**). **HRMS (ESI-TOF) m/z**: calculated for $C_{48}H_{80}N_2O_{15}P_3$ $[M-H]^-$ 1017.4777, found 1017.4731. **IR**: ν [cm^{-1}] = 3183, 2918, 2850, 1759, 1692, 1509, 1467, 1379, 1331, 1250, 1219, 1168, 1127, 1083, 1009, 913, 839, 784, 768, 721, 644, 515, 493, 427.

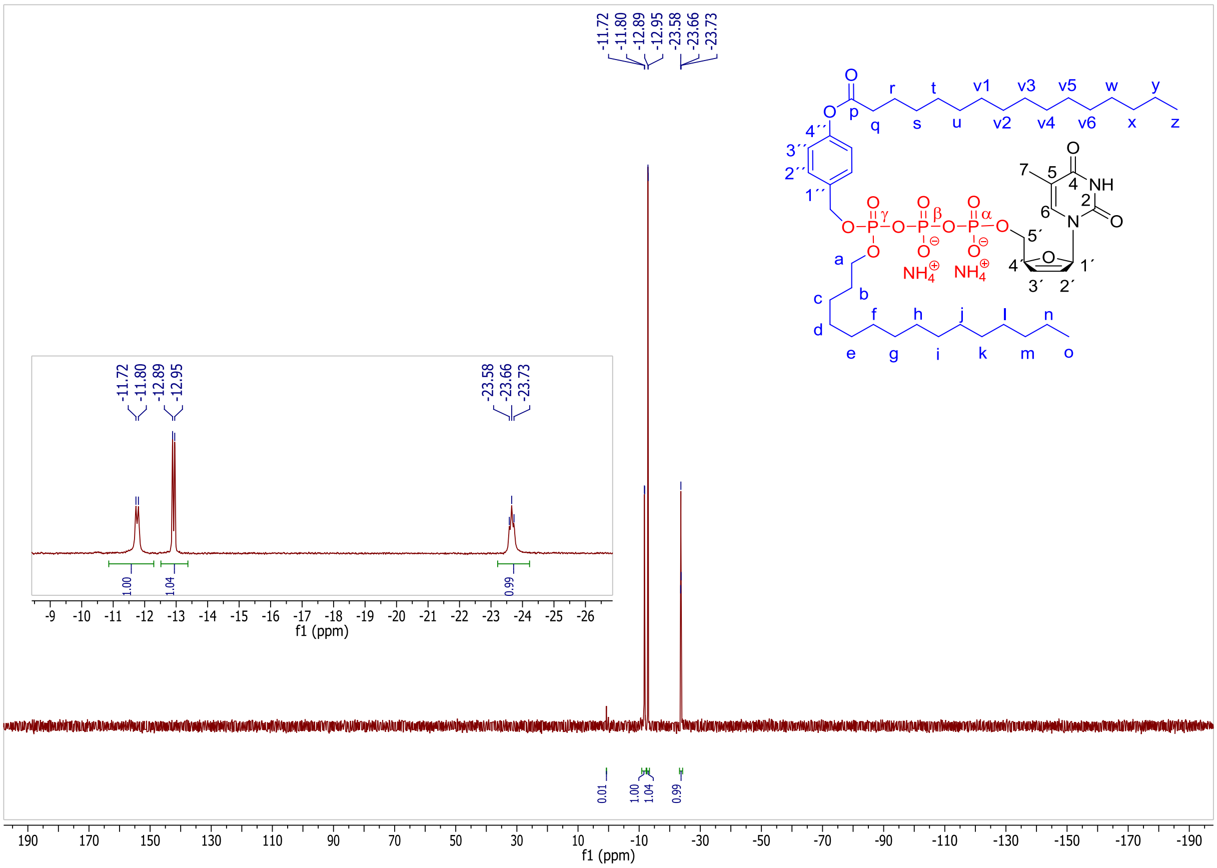

(β-cyanoethyl,alkyl-C_4H_9)-*H*-phosphonate 10w

According to general procedure 1, 0.40 mL diphenyl phosphonate (2.1 mmol, 1.05 equiv.) was dissolved in pyridine at 0 °C. 0.14 g 3-hydroxypropionitrile (2.0 mmol, 1.0 equiv.) was added and followed by 0.16 g 1-butanol (2.2 mmol, 1.1 equiv.). The mixture was stirred overnight at room temperature. Column chromatography (SiO_2, petrol ether/ethylacetate/CH_3COOH 2:8:0.005 v/v/v). Yield: 38%, 0.145 g, as colorless oil. **^{1}H-NMR** (400 MHz, $CDCl_3$-d_1): δ 6.90 (d, *J* = 712 Hz, 1H), 4.30 (dt, *J* = 8.9 Hz, 6.2 Hz, 2H), 4.22-4.05 (m, 2H), 2.77 (t, *J* = 6.6 Hz, 2H), 1.70 (quint, *J* = 7.9 Hz, 2H), 1.49-1.1.36 (m, 2H), 0.95 (t, *J* = 7.4 Hz, 3H). **^{13}C-NMR** (151 MHz, $CDCl_3$-d_1): *δ* 116.27, 66.24 (d, $^2J_{CP}$ = 6.2 Hz), 59.90 (d, $^2J_{CP}$ = 5.3 Hz), 32.26 (d, $^3J_{CP}$ = 6.2 Hz), 20.61, 19.97 (d, $^3J_{CP}$ = 6.5 Hz,), 13.46. **^{31}P-NMR** (243 MHz, $CDCl_3$-d_1): *δ* 7.73. **HRMS (ESI-TOF) m/z**: calculated for $C_7H_{14}NNaO_3P$ $[M+Na]^+$ 214.0604, found 214.0608. **IR**: ν [cm^{-1}] = 2963, 2875, 2254, 1751, 1720, 1467, 1389, 1252, 1214, 1036, 969, 833, 762, 607, 579, 545, 449, 399.

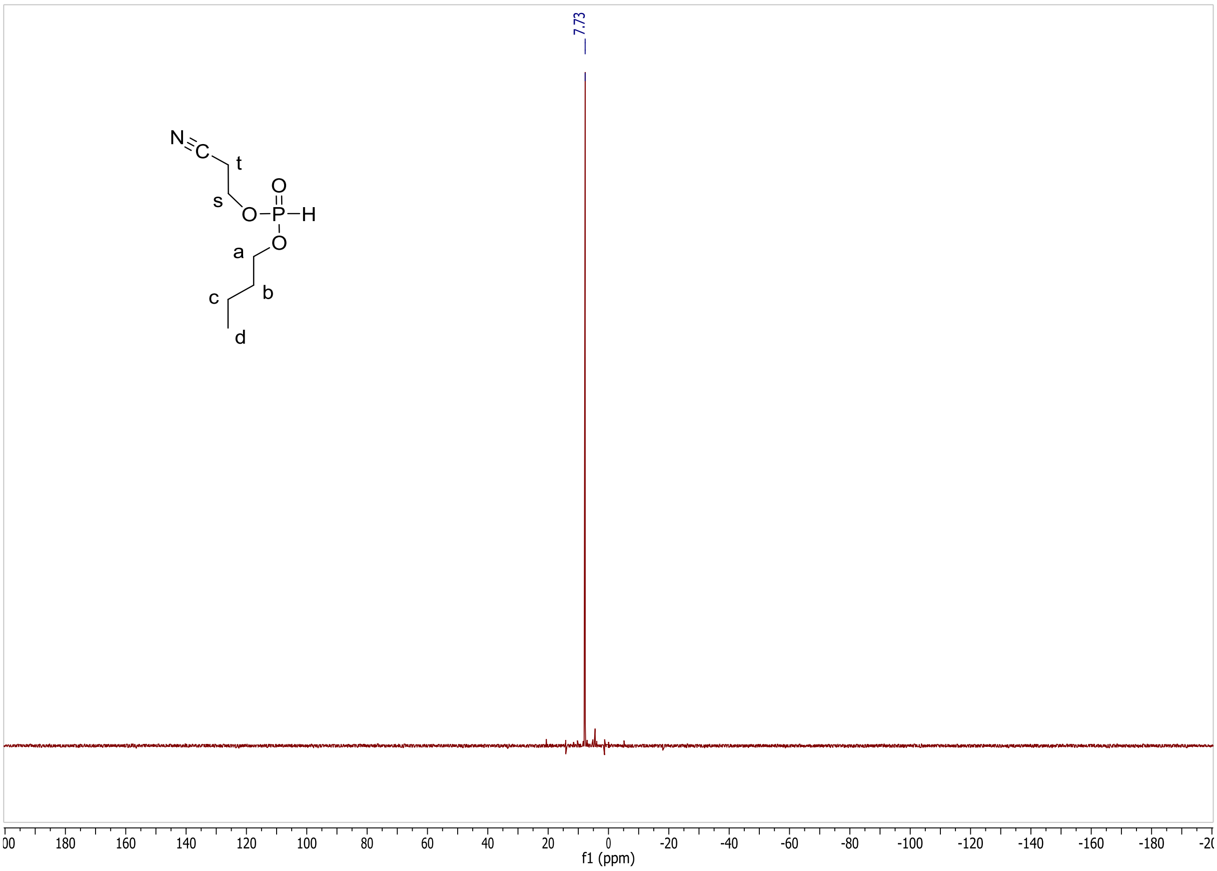

(β-cyanoethyl,alkyl-$C_{11}H_{23}$)-*H*-phosphonate 10x

According to general procedure 1, with 0.49 mL diphenyl phosphonate (2.1 mmol, 1.05 equiv.) at 0 °C; 0.14 g 3-hydroxypropionitrile (2.0 mmol, 1.0 equiv.) was added and following with 0.38 g 1-undecanol **8x** (2.2 mmol, 1.1 equiv.). Reaction time was 8 h at room temperature. Column chromatography (SiO_2, petrol ether/ethyl acetate/CH_3COOH 2:8:0.005 v/v/v). Yield: 54%, 0.31 g, as white solid. **^{1}H-NMR** (400 MHz, $CDCl_3$-d_1): δ 6.89 (d, *J* = 708 Hz, 1H), 4.29 (dt, *J* = 9.0, 6.2 Hz, 2H), 4.18-4.03 (m, 2H), 2.77 (t, *J* = 6.2 Hz, 2H), 1.71 (quint, *J* = 6.5 Hz, 2H), 1.45-1.18 (m, 16H), 0.88 (t, J_{HH} = 6.6 Hz, 3H). **^{13}C-NMR** (101 MHz, $CDCl_3$-d_1): *δ* 116.24, 66.49 (d, $^{2}J_{CP}$ = 6.2 Hz), 59.80 (d, $^{2}J_{CP}$ = 5.3 Hz), 30.33 (d, $^{3}J_{CP}$ = 6.3 Hz), 31.88, 29.55, 29.52, 29.45, 29.29, 29.06, 25.42, 22.66, 20.00 (d, $^{3}J_{CP}$ = 6.5 Hz), 14.09. **^{31}P-NMR** (162 MHz, $CDCl_3$-d_1): *δ* 7.63. **HRMS (ESI-TOF) m/z**: calculated for $C_{14}H_{28}NNaO_3P$ [M+Na]$^+$ 312.1699, found 312.1706. **IR**: ν [cm^{-1}] =2922, 2853, 1750, 1720, 1466, 1415, 1378, 1338, 1249, 1052, 969, 832, 764, 721, 543, 420, 395.

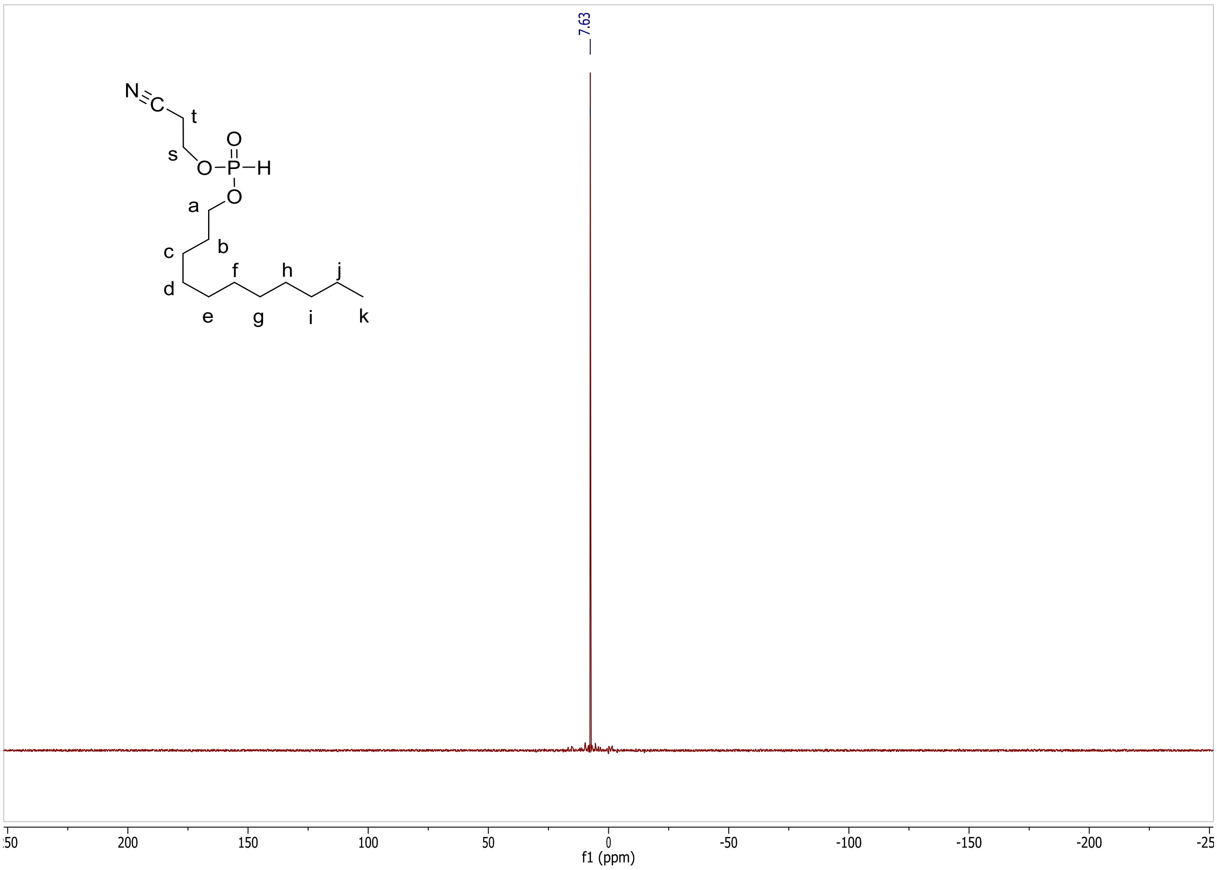

(β-cyanoethyl,alkyl-$C_{18}H_{37}$)-*H*-phosphonate 10z

According to general procedure 1, with 0.23 mL diphenyl phosphonate (1.2 mmol, 1.2 equiv.) at 0 °C; 0.27 g 1-octadecanol **8z** (1.0 mmol, 1.0 equiv.) was added and following with 94 µL 3-hydroxypropionitrile (1.4 mmol, 1.4 equiv.). Reaction time was 3 h at room temperature. Column chromatography (SiO_2, ethyl acetate/CH_3COOH 1:0.005 v/v). Yield: 66%, 0.26 g, as white solid. **^{1}H-NMR** (600 MHz, $CDCl_3$-d_1): δ 6.89 (d, *J* = 690 Hz, 1H), 4.36-4.20 (m, 2H), 4.18-4.05 (m, 2H), 2.77 (t, *J* = 6.2 Hz, 2H), 1.64 (quint, *J* = 6.8 Hz, 2H), 1.42-1.33 (m, 2H), 1.33-1.18 (m, 28H), 0.87 (t, *J* = 7.1 Hz, 3H). **^{13}C-NMR** (151 MHz, $CDCl_3$-d_1): *δ* 116.26, 66.53 (d, *J* = 6.2 Hz), 59.84 (d, *J* = 5.3 Hz), 30.31 (d, *J* = 6.5 Hz), 31.90, 29.67, 29.63, 29.60, 29.52, 29.45, 29.33, 29.06, 25.42, 22.66, 19.99 (d, *J* = 6.5 Hz), 14.09. **^{31}P-NMR** (243 MHz, $CDCl_3$-d_1): *δ* 7.63. **HRMS (ESI-TOF) m/z**: calculated for $C_{21}H_{46}N_2O_3P$ $[M+NH_4]^+$ 405.3241, found 405.3244. **IR**: ν [cm^{-1}] = 3317, 2956, 2916, 2848, 1472, 1462, 1423, 1372, 1332, 1300, 1186, 1124, 1061, 1039, 1022, 1004, 983, 967, 935, 905, 889, 730, 719, 525, 493, 422, 389.

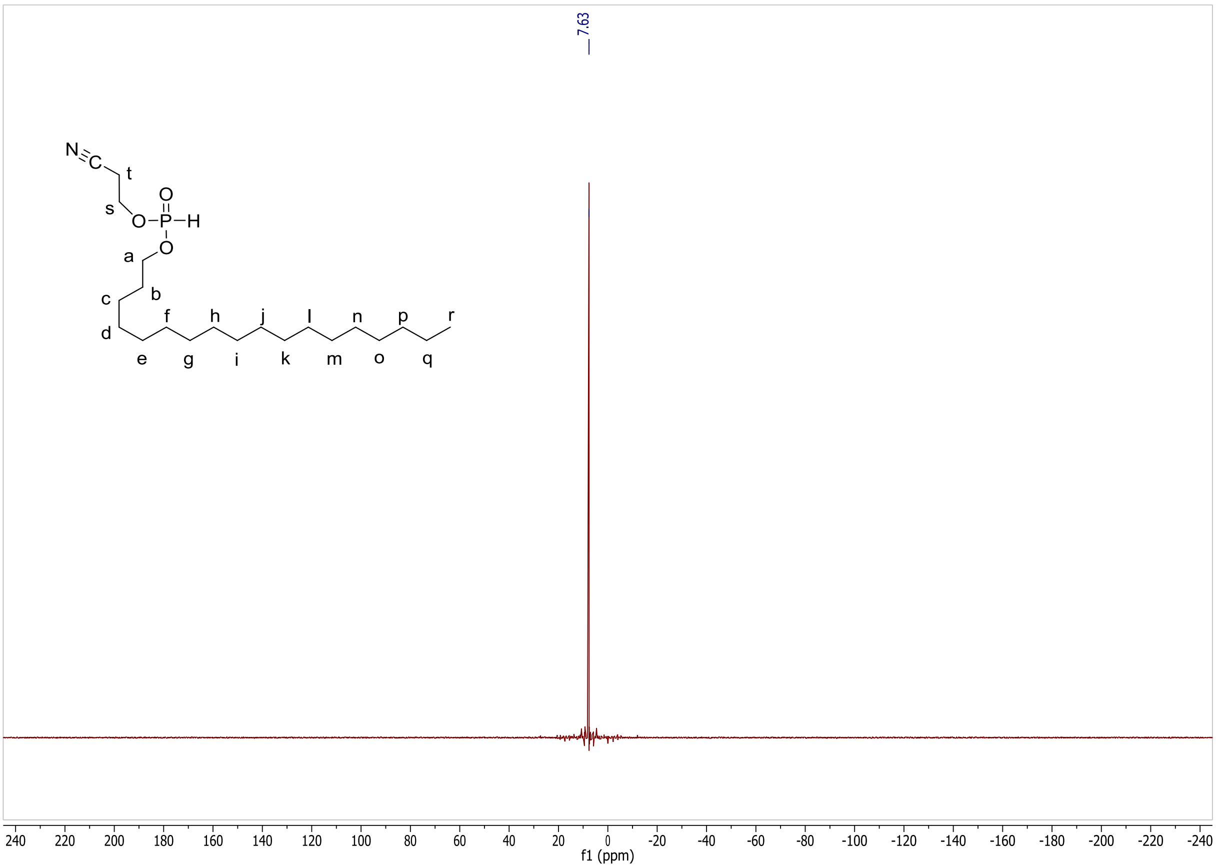

γ-(β-cyanoethyl,alkyl-$C_{18}H_{37}$)-d4TTP 13z

According to general procedure 2, the reactions were performed under dry conditions using 58 mg *H*-phosphonate **10z** (0.15 mmol, 1.0 equiv.), 94 mg d4TMP 2×nBu$_4$N$^+$ salt (0.12 mmol, 0.80 equiv.). Yield: 65%, 63 mg, as white cotton. **^{1}H-NMR** (400 MHz, CD_3OD-d$_4$): *δ* 7.75-7.66 (m, 1H), 7.01-6.95 (m, 1H), 6.57 (dt, *J* = 6.0, 1.8 Hz, 1H), 5.89 (ddd, J_{HH} = 5.9, 2.4, 1.5 Hz, 1H), 5.06-4.96 (m, 1H), 4.39 (dt, *J* = 7.8 Hz, 1.8 Hz, 1H), 4.35-4.14 (m, 4H), 2.94 (t, *J* = 4.8 Hz, 2H), 1.95 (d, *J* = 1.2 Hz, 3H), 1.73 (quint, *J* = 6.8 Hz, 2H), 1.50-1.22 (m, 30H), 0.92 (t, *J* = 7.0 Hz, 3H). **^{13}C-NMR** (101 MHz, CD_3OD-d$_4$): *δ* 166.66, 152.87, 138.73, 135.90, 127.12, 118.63, 112.11, 90.90, 87.22 (d, $^3J_{CP}$ = 9.7 Hz), 70.10 (d, $^2J_{CP}$ = 6.4 Hz), 67.85 (d, $^2J_{CP}$ = 5.1 Hz), 64.22 (d, $^2J_{CP}$ = 5.5 Hz), 31.30 (d, $^3J_{CP}$ = 7.4 Hz), 33.08, 31.33, 31.26, 30.80, 30.76, 30.75, 30.70, 30.48, 30.35, 26.57, 23.74, 20.0 (d, $^3J_{CP}$ = 8.2 Hz), 14.44, 12.48. **^{31}P-NMR** (243 MHz, CD_3OD-d$_4$): *δ* -11.81 (d, $^2J_{PP}$ = 19.9 Hz, **P-α**), -13.59 (d, $^2J_{PP}$ = 16.7 Hz, **P-γ**), -23.78 (t, $^2J_{PP}$ = 18.5 Hz, **P-β**). **HRMS (ESI-TOF) m/z**: calculated for $C_{31}H_{53}N_3O_{13}P_3$ [M-H]$^-$ 768.2797, found 768.2898. **IR**: ν [cm^{-1}] = 3181, 2921, 2851, 1691, 1464, 1247, 1128, 1114, 1079, 1011, 908, 837, 806, 783, 720, 697, 644, 578, 519, 488, 423, 401.

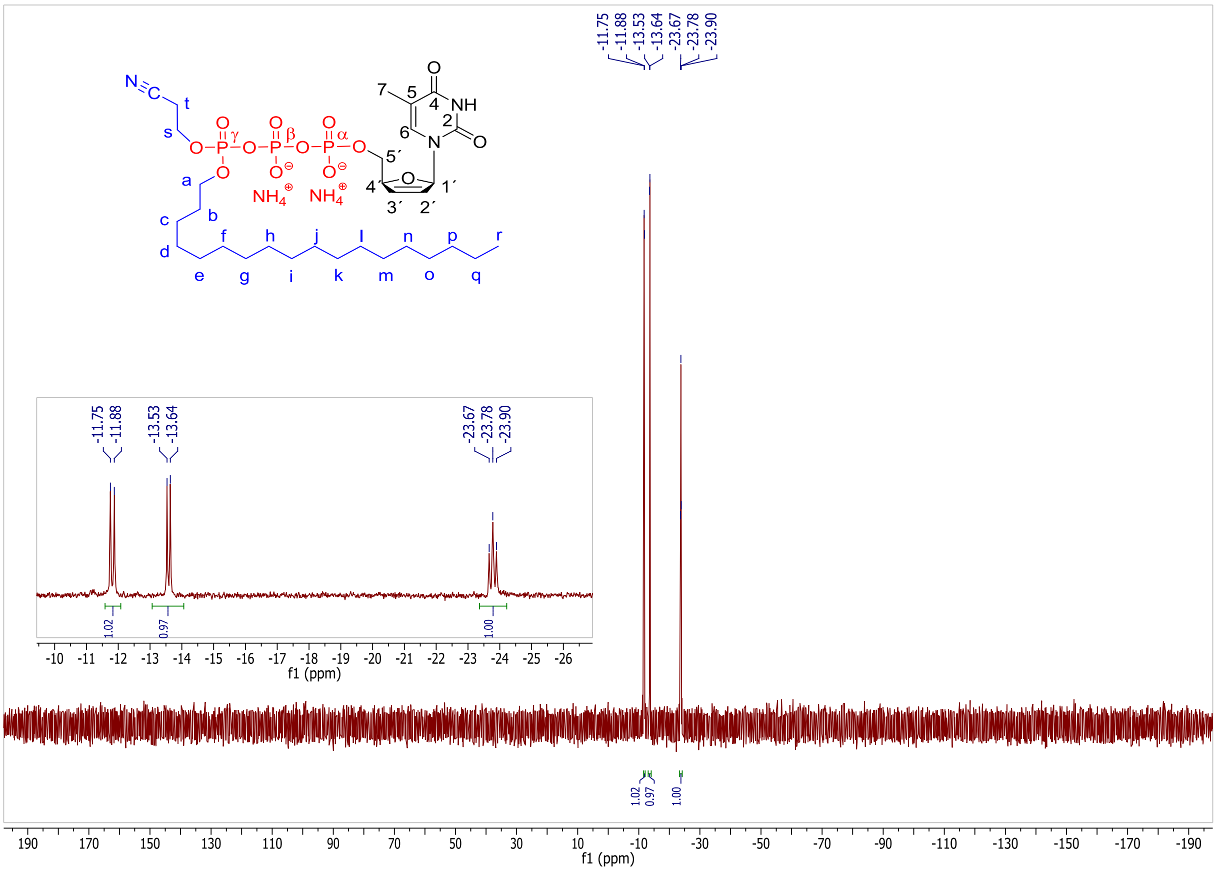

γ-(C_4H_9)-d4TTP 7w

According to general procedure 2, the reactions were performed under dry conditions using 47 mg *H*-phosphonate **10w** (0.15 mmol, 1.0 equiv.), 94 mg d4TMP 2×nBu$_4$N$^+$ salt (0.12 mmol, 0.80 equiv.). After the crude product was concentrated in vacuum, the cleavage of the 9-fluorenylmethyl moiety was achieved in a mixture of 5 mL CH_3CN and 0.97 mL 40% nBu$_4$N$^+$OH$^-$ in H_2O (1.50 mmol, 10 equiv.) and then stirred for 8 h at room temperature followed by automatic rp18 flash-chromatography. The counterion was exchanged to the ammonium-form with Dowex 50WX8 ion-exchange resin and then purified with rp18 chromatography. Product-containing fractions were collected and the organic solvent evaporated. The remaining aqueous solutions were freeze-dried and the desired product obtained. Yield: 31%, 21 mg, as colorless solid. **^{1}H-NMR** (600 MHz, CD_3OD-d$_4$): *δ* 7.73 (d, *J* = 1.3 Hz, 1H), 6.98 (dt, *J* = 3.1, 1.8 Hz, 1H), 6.58 (dt, *J* = 6.2, 1.7 Hz, 1H), 5.89 (dt, *J* = 6.0, 2.4 Hz, 1H), 5.06-4.99 (m, 1H), 4.33-4.14 (m, 2H), 3.91-3.80 (m, 2H), 1.92 (d, *J* = 1.4 Hz, 3H), 1.66 (quint, *J* = 6.9 Hz, 2H), 1.50-1.36 (m, 2H), 0.96 (t, *J* = 7.1 Hz, 3H). **^{13}C-NMR** (151 MHz, CD_3OD-d$_4$): *δ* 166.62, 152.85, 138.67, 135.92, 127.05, 112.01, 90.94, 87.26 (d, $^3J_{CP}$ = 9.1 Hz), 67.41 (d, $^2J_{CP}$ = 5.8 Hz), 67.01 (d, $^2J_{CP}$ = 6.0 Hz) 33.82 (d, $^3J_{CP}$ = 8.1 Hz), 19.98, 14.16, 12.47. **^{31}P-NMR** (162 MHz, CD_3OD-d$_4$): *δ* -9.99 (d, $^2J_{PP}$ = 17.8 Hz, **P-α**), -10.93 (d, $^2J_{PP}$ = 17.1 Hz, **P-γ**), -22.18 (t, $^2J_{PP}$ = 19.4 Hz, **P-β**). **MALDI-MS** (m/z): calculated $C_{14}H_{23}N_2NaO_{13}P_3$ [M+Na]$^+$ 543.031, found 542.986; calculated $C_{14}H_{23}KN_2O_{13}P_3$ [M+K]$^+$ 559.005, found 558.960; calculated $C_{14}H_{22}N_2O_{13}P_3$ [M-H]$^-$ 519.034, found 519.219. **IR**: ν [cm^{-1}] = 3185, 2958, 1661, 1428, 1212, 1114, 1062, 985, 891, 835, 784, 736, 643, 482.

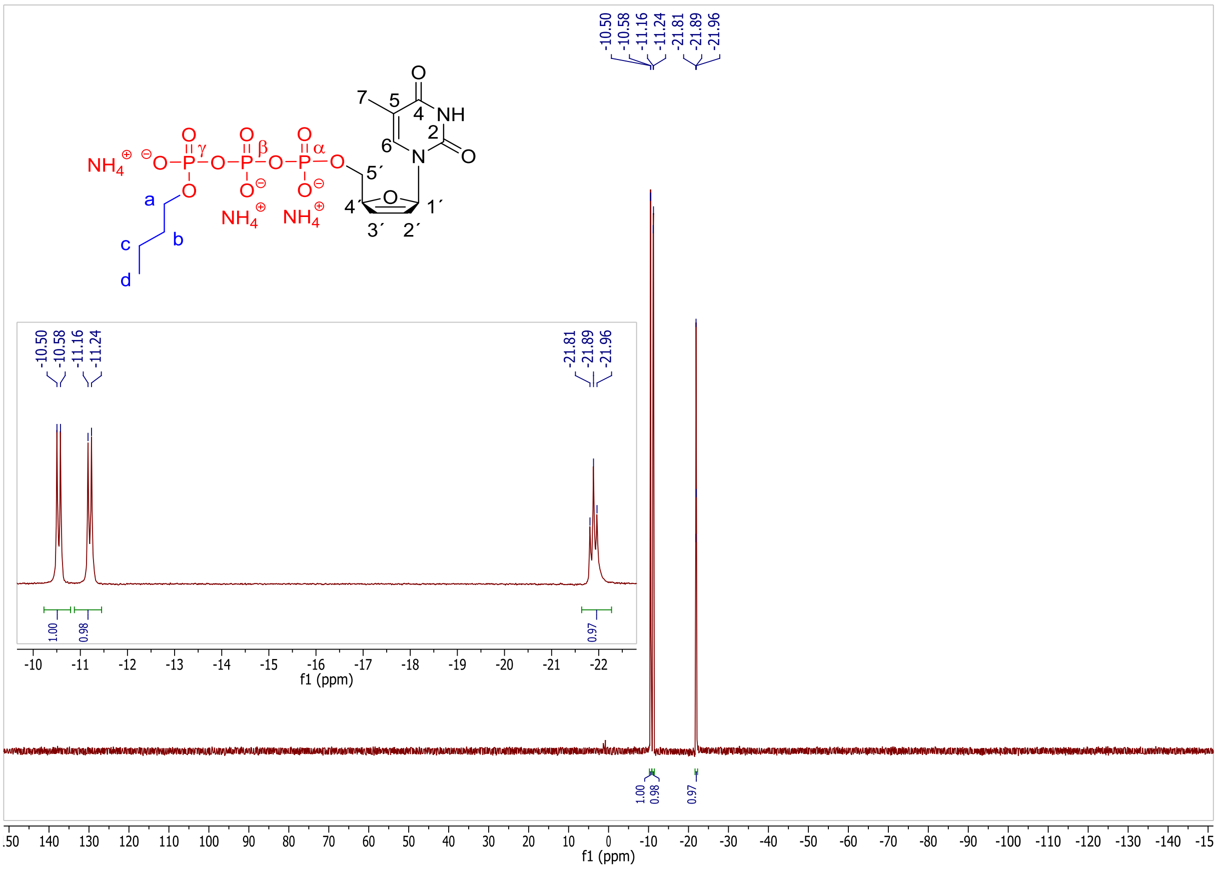

γ-($C_{11}H_{23}$)-d4TTP 7x

According to general procedure 2, the reactions were performed under dry conditions using 43 mg *H*-phosphonate **10x** (0.15 mmol, 1.0 equiv.), 94 mg d4TMP 2×nBu$_4$N$^+$ salt (0.12 mmol, 0.80 equiv.). After the crude product was concentrated in vacuum, the cleavage of the β-cyanoethyl moiety was achieved in a mixture of 5 mL CH_3CN and 0.97 mL 40% nBu$_4$N$^+$OH$^-$ in H_2O (1.50 mmol, 10 equiv.) and then stirred for 8 h at room temperature followed by automatic rp18 flash chromatography. The counterion was exchanged to the ammonium-form with Dowex 50WX8 ion-exchange resin and then purified with RP18 chromatography. Product-containing fractions were collected and the organic solvent evaporated. The remaining aqueous solutions were freeze-dried and the desired product obtained. Yield: 28%, 23 mg, as white cotton. **^{1}H-NMR** (600 MHz, CD_3OD-d$_4$): *δ* 7.73 (d, *J* = 1.3 Hz, 1H), 6.97 (dt, *J* = 3.4, 1.7 Hz, 1H), 6.59 (dt, *J* = 6.1, 1.7 Hz, 1H), 5.88 (dt, *J* = 6.1, 2.4 Hz, 1H), 5.07-4.98 (m, 1H), 4.31 (ddd, *J* = 11.6, 6.8, 3.4 Hz, 1H), 4.20 (ddd, *J* = 11.6, 5.5, 3.2 Hz, 1H), 4.00 (q, *J* = 6.6 Hz, 2H), 1.94 (d, *J* = 1.2 Hz, 3H), 1.66 (quint, *J* = 6.9 Hz, 2H), 1.48-1.26 (m, 16H), 0.92 (t, *J* = 7.0 Hz, 3H). **^{13}C-NMR** (151 MHz, CD_3OD-d$_4$): *δ* 166.61, 152.85, 138.67, 135.91, 127.07, 112.02, 90.94, 87.25 (d, $^3J_{CP}$ = 9.1 Hz), 67.79 (d, $^2J_{CP}$ = 5.8 Hz), 67.39 (d, $^2J_{CP}$ = 5.8 Hz), 33.06, 31.82 (d, $^3J_{CP}$ = 8.1 Hz), 30.78, 30.76, 30.58, 30.47, 26.90, 23.73, 14.43, 12.47. **^{31}P-NMR** (243 MHz, CD_3OD-d$_4$): *δ* -10.76 (d, $^2J_{PP}$ = 19.3 Hz, **P-α**), -11.48 (d, $^2J_{PP}$ = 19.1 Hz, **P-γ**), -22.48 (t, $^2J_{PP}$ = 19.2 Hz, **P-β**). **MALDI-MS** (m/z): calculated $C_{21}H_{37}N_2NaO_{13}P_3$ [M+Na]$^+$ 641.140, found 641.105. **IR**: ν [cm^{-1}] = 2922, 2852, 1688, 1661, 1429, 1217, 1126, 1064, 1045, 991, 900, 836, 783, 768, 736, 644, 488, 427, 400.

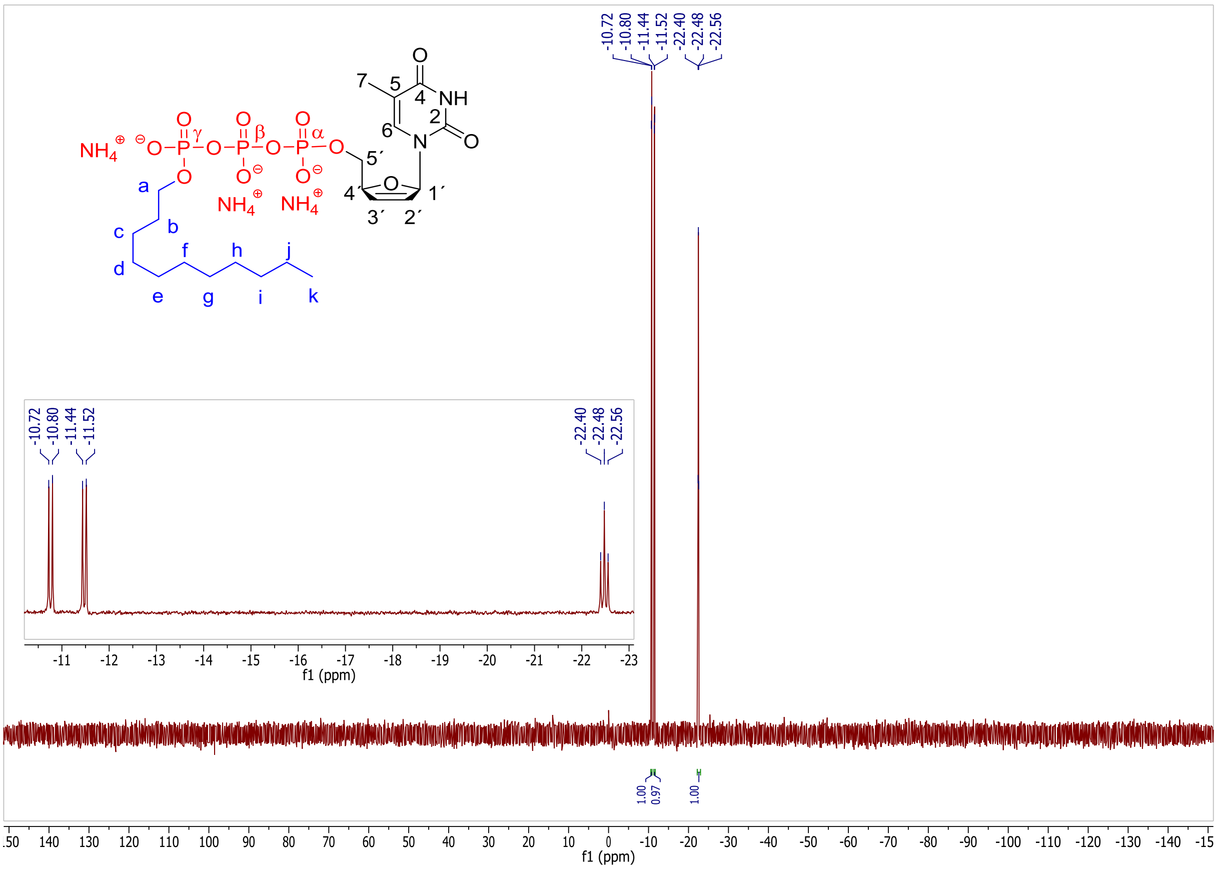

γ-($C_{18}H_{37}$)-d4TTP 7z

According to general procedure 2, the reactions were performed under dry conditions using 58 mg *H*-phosphonate **10z** (0.15 mmol, 1.0 equiv.), 94 mg d4TMP 2×nBu$_4$N$^+$ salt (0.12 mmol, 0.80 equiv.). After the crude product was purified by automatic rp18 flash chromatography, the cleavage of the β-cyanoethyl-moiety was achieved in a mixture of 5 mL CH_3CN and 0.97 mL 40% nBu$_4$N$^+$OH$^-$ in H_2O (1.50 mmol, 10 equiv.) and then stirred for 20 h at room temperature followed by ion-exchange to the ammonium-form with Dowex 50WX8 ion-exchange resin and a second rp18 chromatography purification step. Product-containing fractions were collected and the organic solvent evaporated. The remaining aqueous solutions were freeze-dried and the desired product obtained. Yield: 46%, 43 mg, as white solid. **^{1}H-NMR** (600 MHz, CD_3OD-d$_4$): *δ* 7.75-7.66 (m, 1H), 7.02-6.93 (m, 1H), 6.62-6-53 (m, 1H), 5.85 (dt, *J* = 5.9, 2.8 Hz, 1H), 5.06-5.00 (m, 1H), 4.35-4.14 (m, 2H), 4.00 (q, *J* = 6.7 Hz, 2H), 1.93 (d, *J* = 1.2 Hz, 3H), 1.66 (quint, *J* = 6.8 Hz, 2H), 1.48-1.23 (m, 30H), 0.92 (t, *J* = 7.0 Hz, 3H). **^{13}C-NMR** (101 MHz, CD_3OD-d$_4$): *δ* 166.64, 152.86, 138.69, 135.86, 127.13, 112.04, 90.91, 87.22 (d, *J* = 9.2 Hz), 67.80 (d, *J* = 6.0 Hz), 67.54 (d, *J* = 5.8 Hz), 31.77 (d, *J* = 8.1 Hz), 33.08, 30.80, 30.76, 30.58, 30.48, 26.89, 23.74, 14.45, 12.48. **^{31}P-NMR** (243 MHz, CD_3OD-d$_4$): *δ* -10.84 (d, *J* = 18.9 Hz, **P-α**), -11.46 (d, *J* = 18.6 Hz, **P-γ**), -22.57 (t, *J* = 18.9 Hz, **P-β**). **HRMS (ESI-TOF) m/z**: calculated for $C_{28}H_{50}N_2O_{13}P_3$ [M-H]$^-$ 715.2531, found 715.2518. **IR**: ν [cm^{-1}] = 3190, 3025, 2921, 2851, 1689, 1455, 1221, 1128, 1067, 1045, 994, 904, 838, 784, 768, 737, 721, 644, 489, 424, 402.

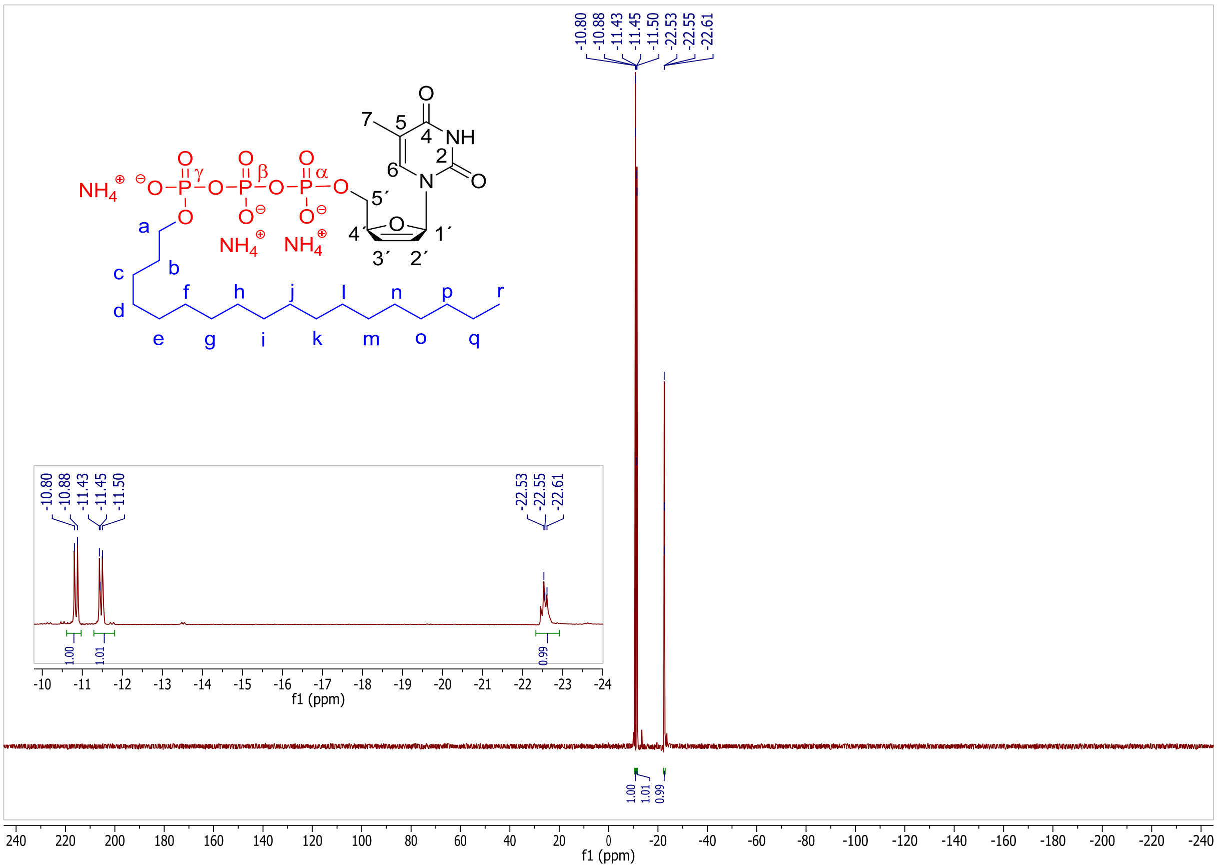

γ-(C_4H_9)-TTP 15w

According to general procedure 2 the reactions were performed under dry conditions using 29 mg H-phosphonate **10w** (0.15 mmol, 1.0 equiv.), 94 mg d4TMP 2×nBu4N+ salt (0.12 mmol, 0.80 equiv.).After the crude product was concentrated in vacuum, the cleavage of the protection group was achieved in a mixture of 5 mL CH3CN and 0.97 mL 40% $n\text{Bu}_4\text{N}^+\text{OH}^-$ in H2O (1.50 mmol, 10 equiv.) and then stirred for 8 h at room temperature followed by automatic rp18 flash chromatography. The counter ion was exchanged to the ammonium-form with Dowex 50WX8 ion-exchange resin and then purified with rp18 chromatography. Product-containing fractions were collected and the organic solvent evaporated. The remaining aqueous solutions were freeze-dried and the desired product obtained. Yield: 27%, 19 mg, as colorless solid. **^{1}H-NMR** (600 MHz, CD_3OD-d_4): δ 7.83 (s, 1H), 6.32 (t, J = 6.7 Hz, 1H), 4.61 (dt, J_{HH} = 6.7, 3.4 Hz), 4.31-4.16 (m, 2H), 4.08-4.03 (m, 1H), 4.06 (q, J = 6.6 Hz, 2H), 2.35-2.20 (m, 2H), 1.96 (d, J = 1.3 Hz, 3H), 1.64 (quint, J = 6.7 Hz, 2H), 1.44 (m, 2H), 0.95 (t, J = 7.4 Hz, 3H). **^{13}C-NMR** (151 MHz, CD_3OD-d_4): δ 166.52, 152.43, 138.22, 111.93, 87.30 (d, $^3J_{CP}$ = 8.9 Hz), 86.15, 72.08, 67.04 (d, J = 6.2 Hz), 66.63 (d, J = 5.4 Hz), 40.61, 33.85 (d, $^3J_{CP}$ = 8.0 Hz), 19.99, 14.19, 12.61. **^{31}P-NMR** (243 MHz, CD_3OD-d_4): δ -10.54 (d, $^2J_{PP}$ = 18.2 Hz, **P-α**), -11.20 (d, $^2J_{PP}$ = 18.0 Hz, **P-γ**), -21.89 (t, $^2J_{PP}$ = 18.1 Hz, **P-β**). **MALDI-MS** (m/z): calculated $C_{14}H_{25}N_2NaO_{14}P_3$ $[M+Na]^+$ 561.041, found 560.988. **IR**: ν [cm^{-1}] = 3170, 2958, 1659, 1427, 1209, 1124, 1060, 995, 915, 890, 824, 728, 616, 489, 414.

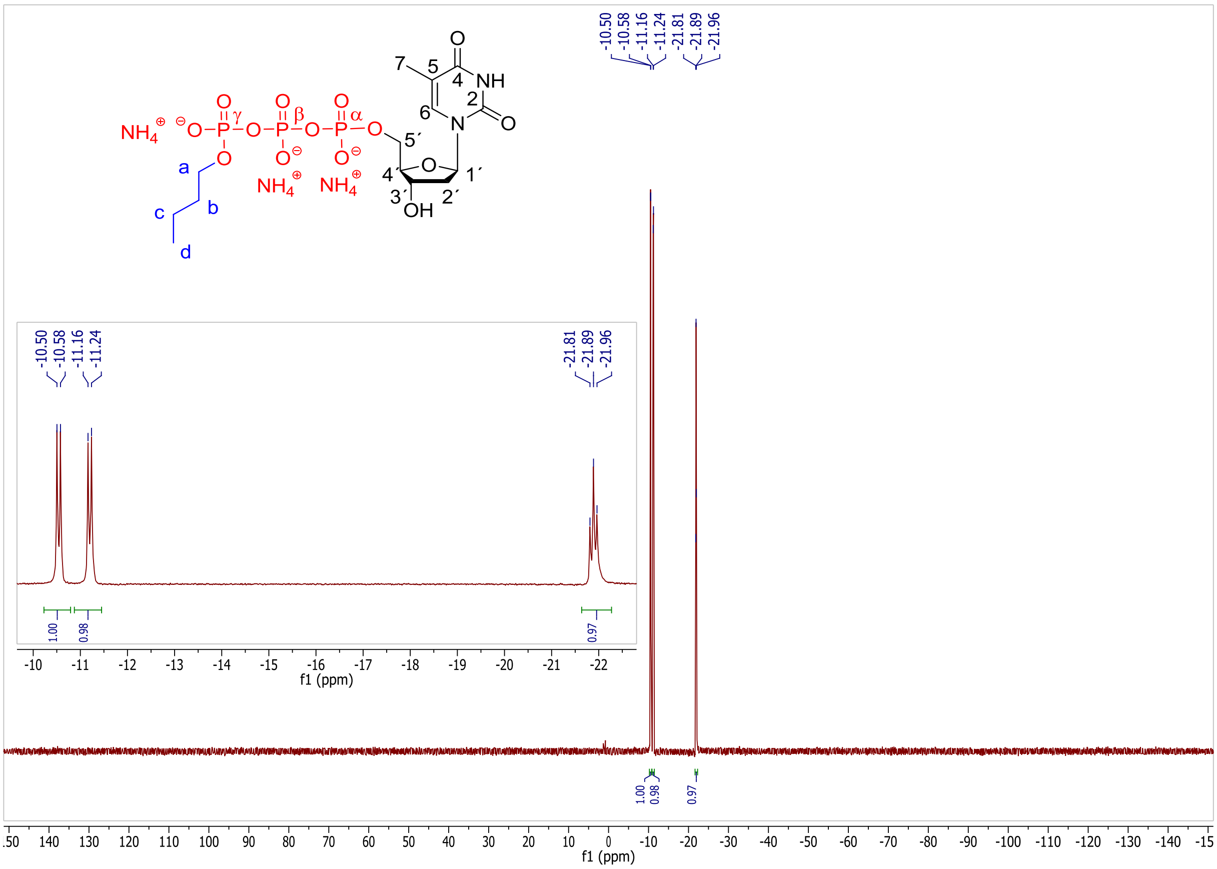

γ-($C_{18}H_{37}$)-TTP 15z

According to general procedure 2, the reactions were performed under dry conditions using 58 mg *H*-phosphonate **10z** (0.15 mmol, 1.0 equiv.), 97 mg dTMP 2×nBu_4N^+ salt (0.12 mmol, 0.80 equiv.). After the crude product was concentrated in vacuum, the cleavage of the cleavage of the acyloxybenzyl-moiety was achieved in a mixture of 5 mL CH_3CN and 0.97 mL 40% $nBu_4N^+OH^-$ in H_2O (1.50 mmol, 10 equiv.) and then stirred for 8 h at room temperature followed by automatic RP18 flash chromatography. The counter ion was exchanged to the ammonium-form with Dowex 50WX8 ion-exchange resin and then purified with RP18 chromatography. Product-containing fractions were collected and the organic solvent evaporated. The remaining aqueous solutions were freeze-dried and the desired product obtained. Yield: 43%, 41 mg, as white cotton. **^{1}H-NMR** (600 MHz, CD_3OD-d_4): *δ* 7.85 (q, *J* = 1.2 Hz, 1H), 6.33 (t, *J* = 6.2 Hz, 1H), 4.63 (dt, *J* = 6.2 Hz, 3.2 H), 4.34-4.18 (m, 2H), 4.08-3.98 (m, 3H), 2.33-2.21 (m, 2H), 1.96 (d, $^4J_{HH}$ = 1.3 Hz, 3H), 1.67 (quint, *J* = 6.8 Hz, 2H), 1.46-1.26 (m, 30H), 0.92 (t, *J* = 7.0 Hz, 3H). **^{13}C-NMR** (151 MHz, CD_3OD-d_4): *δ* 166.54, 152.43, 138.17, 111.96, 87.39 (d, $^3J_{CP}$ = 9.1 Hz), 86.03, 72.05, 67.58 (d, $^2J_{CP}$ = 6.0 Hz), 66.69 (d, $^2J_{CP}$ = 5.4 Hz), 40.70, 33.07, 31.77 (d, $^3J_{CP}$ = 7.8 Hz), 30.81, 30.79, 30.75, 30.57, 30.46, 26.88, 23.73, 14.43, 12.60. **^{31}P-NMR** (243 MHz, CD_3OD-d_4): *δ* -10.97 (d, $^2J_{PP}$ = 19.3 Hz, **P-α**), -11.56 (d, $^2J_{PP}$ = 19.5 Hz, **P-γ**), -22.82 (t, $^2J_{PP}$ = 19.4 Hz, **P-β**). **MALDI-MS** (m/z): calculated $C_{28}H_{53}N_2NaO_{14}P_3$ $[M+Na]^+$ 757.260, found 757.216; calculated $C_{28}H_{53}KN_2O_{14}P_3$ $[M+K]^+$ 773.234, found 773.190. **IR**: ν [cm^{-1}] = 3185, 3027, 2918, 2850, 1681, 1465, 1432, 1222, 1124, 1066, 996, 921, 854, 822, 720, 494, 423, 380.

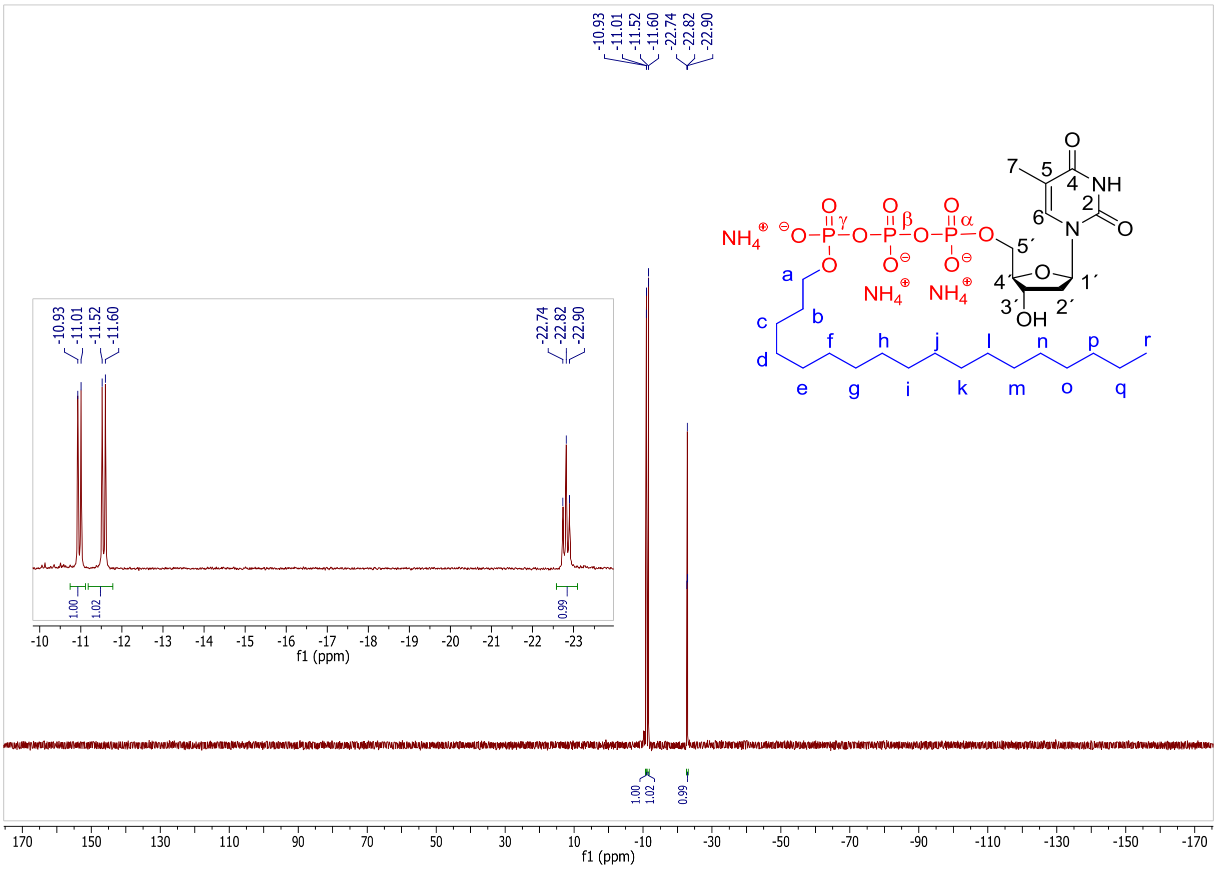

Synthesis of non-symmetric (AB, AB) *H*-phosphonates 5

γ-(AB-C_4H_9,AB-$C_{17}H_{31}$)-*H*-phosphonate 16b

According to general procedure 1 with 0.23 mL diphenyl phosphonate (1.2 mmol, 1.2 equiv.) at 0 °C; 0.391 g 4-(hydroxymethyl)phenyloctadecanoate (1.0 mmol, 1.0 equiv.) was added and following with 0.292 g 4-(hydroxymethyl)phenylpentanoate**9c** (1.4 mmol, 1.4 equiv.). Reaction time was 3 h at room temperature. Column chromatography (SiO_2, petrol ether/ethyl acetate/CH_3COOH 8:2:0.005 v/v/v). Yield: 43%, 0.280 g, as white solid. **^{1}H NMR** (600 MHz, $CDCl_3$-d_1): δ 7.39-7.33 (m, 4H), 7.11-7.05 (m, 4H), 6.93 (d, *J* = 708 Hz, 1H), 5.16-4.91 (m, 4H), 2.56 (t, *J* = 7.5 Hz, 2H), 2.55 (t, *J* = 7.5 Hz, 2H), 1.74 (pd, *J* = 7.6, 3.2 Hz, 4H), 1.49-1.20 (m, 30H), 0.97 (t, *J* = 7.3 Hz, 3H), 0.88 (t, *J* = 7.0 Hz, 3H). **^{13}C-NMR** (151 MHz, $CDCl_3$-d_1): *δ* 172.08,172.06, 151.0, 132.95, 132.91, 129.2, 121.90, 66.67,66.63, 34.3, 34.1, 31.9, 29.66, 29,64, 29.62, 29.61, 29.57, 29.43, 29.33, 29.22, 29.08, 26.9, 24.9, 22.7, 22.2, 14.1, 13.7. **^{31}P-NMR** (243 MHz, Chloroform-d): *δ* 7.71. **HRMS (ESI-TOF) m/z**: calculated for $C_{37}H_{61}NO_7P$ $[M+NH_4]^+$ 662.4180, found 662.4200. **IR**: ν [cm^{-1}] =2956, 2915, 2848, 1748, 1608, 1510, 1465, 1383, 1251, 1235, 1220, 1167, 1150, 1104, 1062, 1010, 997, 925, 878, 834, 769, 720, 510.

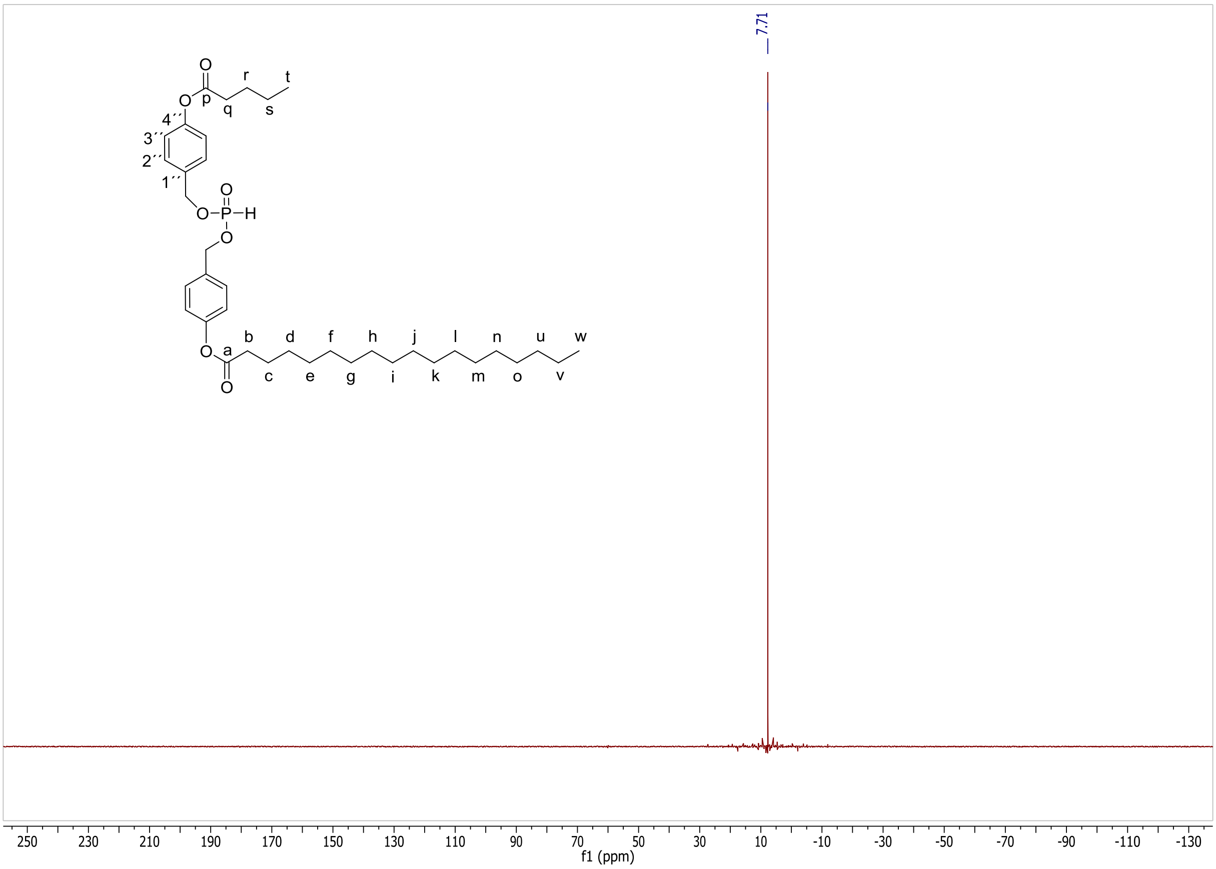

Synthesis of the γ-non-symmetric-(AB,AB)-NTPs prodrug

γ-(AB-C_4H_9, AB-$C_{17}H_{35}$)-d4TTP 5b

According to general procedure 2, the reactions were performed under dry conditions using 97 mg *H*-phosphonate **16b** (0.15 mmol, 1.0 equiv.) and 118 mg d4TMP 2×nBu$_4$N$^+$ salt (0.15 mmol, 1.0 equiv.). Yield: 40%, 64 mg, as white cotton. **^{1}H-NMR** (600 MHz, CD_3OD-d$_4$): *δ* 7.71-7.66 (m, 1H), 7.44-7.38 (m, 4H), 7.09-7.04 (m, 4H), 6.94 (dt, *J* = 3.5, 1.6 Hz, 1H), 6.48 (dt, *J* = 6.0, 1.8 Hz, 1H), 5.81 (ddd, *J* = 6.1, 2.4, 1.3 Hz, 1H), 5.17 (d, *J* = 8.2 Hz, 4H), 4.98-4.93 (m, 1H), 4.33-4.15 (m, 2H), 2.63-2.56 (m, 4H), 1.91 (d, *J* = 1.2 Hz, 3H), 1.79-1.69 (m, 4H), 1.52-1.23 (m, 30H), 1.01 (t, *J* = 7.4 Hz, 3H), 0.92 (t, *J* = 7.0 Hz, 3H). **^{13}C-NMR** (151 MHz, CD_3OD-d$_4$): *δ* 173.77, 166.53, 152.76, 152.35, 138.67, 135.76, 134.93 (d, $^3J_{CP}$ = 7.5 Hz), 130.48 (d, $^4J_{CP}$ = 2.9 Hz), 127.16, 122.88, 112.05, 90.83, 87.20 (d, $^3J_{CP}$ = 9.1 Hz), 70.38, 67.87 (d, $^2J_{CP}$ = 5.6 Hz), 35.03, 34.76, 33.07, 30.79, 30.77, 30.75, 30.72, 30.61, 30.47, 30.42, 30.18, 28.07, 25.96, 23.74, 23.26, 14.45, 14.11, 12.49. **^{31}P-NMR** (243 MHz, CD_3OD-d$_4$): *δ* -11.77 (d, *J* = 19.6 Hz, **P-α**), -13.20 (d, *J* = 17.3 Hz, **P-γ**), -23.70 (t, *J* = 18.1 Hz, **P-β**). **HRMS (ESI-TOF) m/z**: calculated for $C_{47}H_{68}N_2O_{17}P_3$ [M-H]$^-$ 1025.3736, found 1025.3703. **IR**: ν [cm^{-1}] = 3040, 2920, 2851, 1755, 1690, 1509, 1465, 1380, 1249, 1219, 1168, 1128, 1082, 1007, 910, 838, 784, 768, 721, 644, 491, 420.

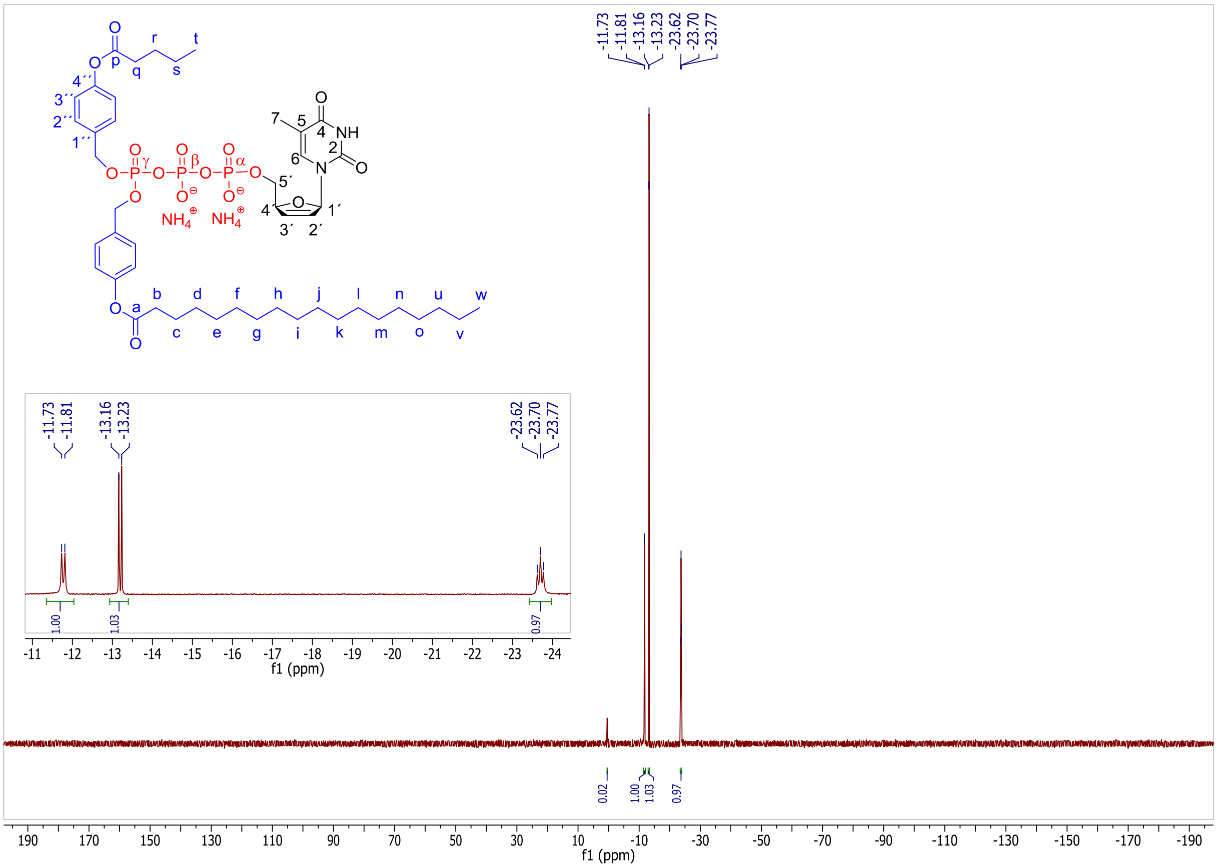

Results and Discussion

Delivery mechanism of the Tri*PPP*ro-compounds **5**

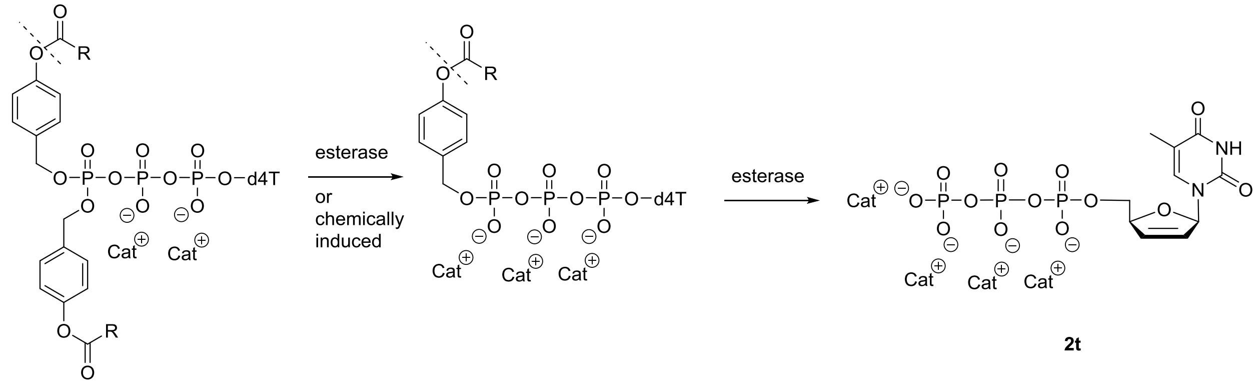

Scheme SI1: Examples for nucleoside triphosphate prodrugs (Tri*PPP*ro's) and their delivery pathway.

Figure SI1: γ-C18-d4TTP **7z** in PBS (pH 7.3).

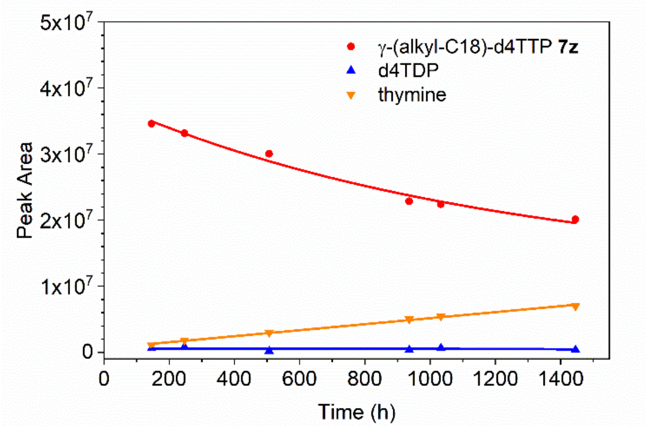

Figure SI2: γ-C18-d4TTP **7z** in CEM/0 cell extracts.

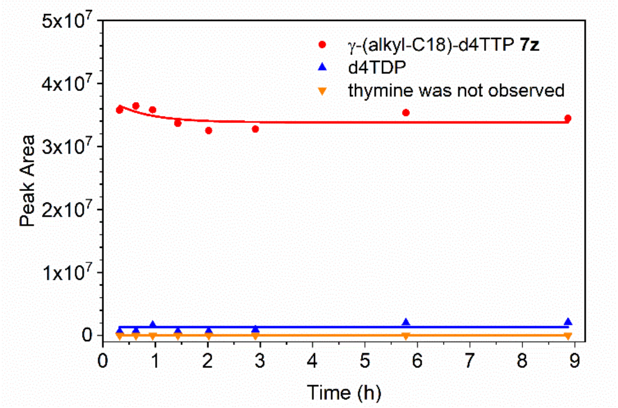

Figure SI3: Primer extension experiment with incubation period of 15 min using 6U:

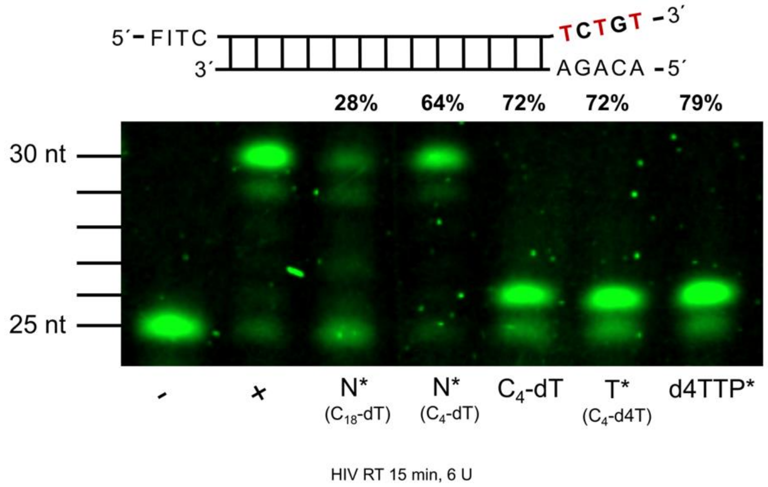

lanes (-): A, G, C, T without HIV RT.
lanes (+): A, G, C, T with HIV RT
lanes (N*): A, G, C and γ-(Alkyl-$C_{18}H_{37}$)-TTP **15z**
lanes (N*): A, G, C and γ-(Alkyl-C_4H_9)-TTP **15w**
lanes (C_4-dT): γ-(Alkyl-C_4H_9)-TTP **15w**
lanes (T*): A, G, C and γ-(alkyl-C_4H_9)-d4TTP **7w**
lanes (d4TTP*): A, G, C and d4TTP

Assay condition:
50 mM Tris-HCl (pH 8.6 at 22 °C), 10 mM $MgCl_2$, 40mM KCl, dNTPs 66 µM, HIV RT 6 U, Hybrid 0.32 µM in a reaction volume of 10 µL, incubated 37 °C for 15 min, 80 °C for 3 min; 50 mA, 45w for 4 h.**Figure SI4:** Primer extension experiment with incubation period of 30 min using 6U HIV RT:

Figure SI4: HIV RT, 6U 30 min:

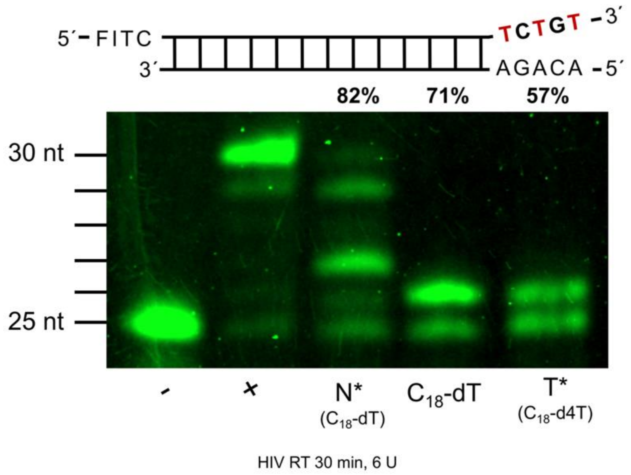

lanes (-): A, G, C, T without HIV RT.
lanes (+): A, G, C, T with HIV RT
lanes (N*): A, G, C and γ-(alkyl-$C_{18}H_{37}$)-TTP **15z**
lanes (T*): γ-(Alkyl-$C_{18}H_{37}$)-TTP **15z**
lanes (d4T*): A, G, C and γ-(Alkyl-$C_{18}H_{37}$)-d4TTP **7z**

Assay condition:
50 mM Tris-HCl (pH 8.6 at 22 °C), 10 mM $MgCl_2$, 40mM KCl, dNTPs 66 µM, HIV RT 6 U, Hybrid 0.32 µM in a reaction volume of 10 µL, incubated 37 °C for 30 min, 80 °C for 3 min; 50 mA, 45w for 4 h.

Figure SI5: Primer extension experiment with different incubation period using 6U HIV RT:

(γ-C4-d4TTP, γ-C11-d4TTP vs γ-C18-d4TTP from 10 min, 20 min, 40 min, 60min)

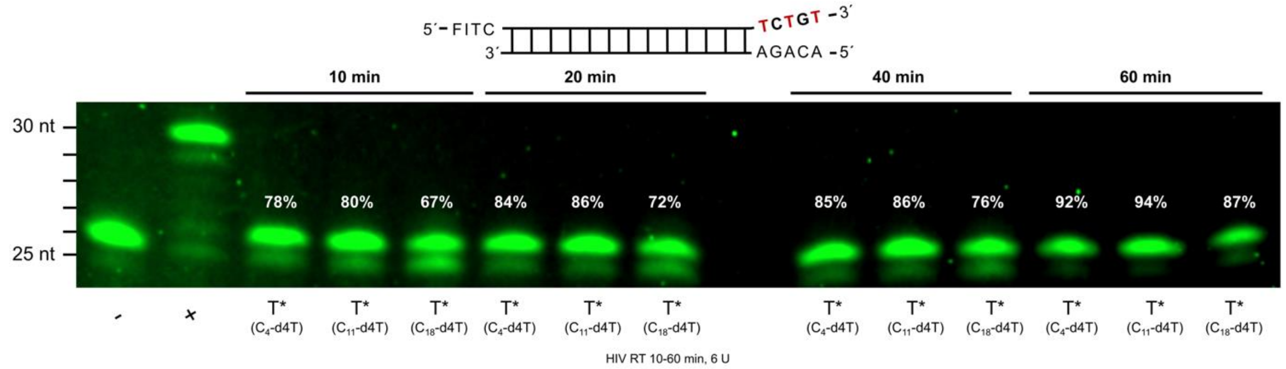

lanes (-): A, G, C, T without HIV RT.
lanes (+): A, G, C, T with HIV RT
lanes (T*): A, G, C and γ-modified-d4TTP

Assay condition:
50 mM Tris-HCl (pH 8.6 at 22 °C), 10 mM $MgCl_2$, 40mM KCl, NTPs 66 µM, HIV RT 6 U, Hybrid 0.32 µM in a reaction volume of 10 µL, incubated 37 °C for 10 min, 20 min, 40 min and 60 min, 80 °C for 3 min; 50 mA, 45w for 4 h. The conversion rate of the primer to the n+1 product was calculated using the software of ImageJ.

Figure SI6: Primer extension assay using DNA polymerase γ and the γ-alkyl-dNTP **7** and **15**.

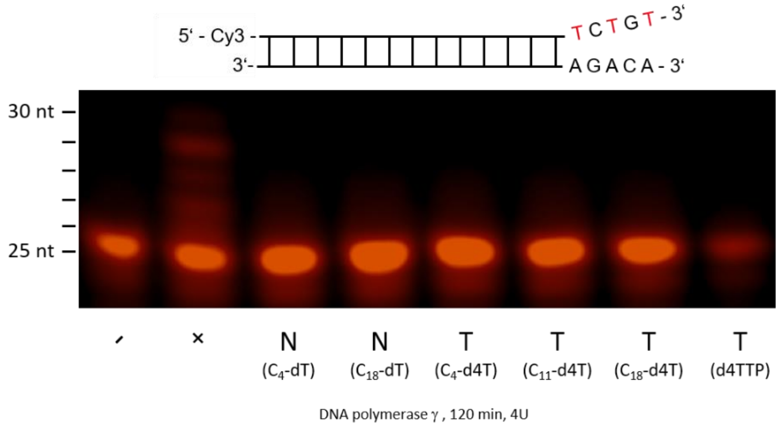

Lane 1 (-): dATP, dGTP, dCTP and TTP without human Pol γ; lane 2 (+): dATP, dGTP, dCTP and TTP with human Pol γ; lane 3 (C_4-T): γ-C4-TTP **14w**; lane 4 (C_{18}-T): γ-C18-TTP **15z**; lane 5 (T, C_4-d4T): γ-C4-d4TTP **7w**; lane 6 (T, C_{11}-d4T): γ-C11-d4TTP **7x**; lane 7 (T, C_{18}-d4T): γ-C18-d4TTP **7z**; lane 8 (d4TTP): d4TTP.

Assay condition: 60 mM Tris-HCl (pH 8), 5 mM MgOAc, 1.0 mM dithiothreitol, 0.01% (w/m) of bovine serum albumin, dNTPs 250 µM, DNA polymerase γ 4 U, hybrid 0.20 µM in a reaction volume of 25 µL, incubated 37 °C for 5 min without dNTPs, then incubated 120 min at 37°C, 80 °C for 3 min; 50 mA, 45w for 3 h.

Figure SI7: Primer extension assay using DNA polymerase α and the γ-alkyl-dNTP **7** and **15**.

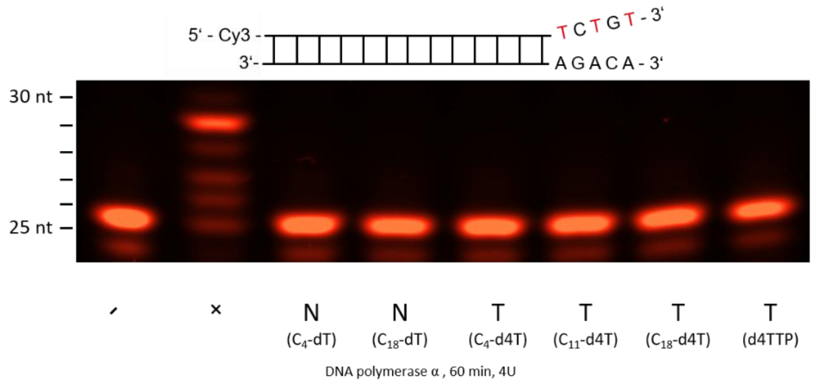

Lane 1 (-): dATP, dGTP, dCTP and TTP without human Pol α; lane 2 (+): dATP, dGTP, dCTP and TTP with human Pol α; lane 3 (C_4-T): γ-C4-TTP **14w**; lane 4 (C_{18}-T): γ-C18-TTP **15z**; lane 5 (T, C_4-d4T): γ-C4-d4TTP **7w**; lane 6 (T, C_{11}-d4T): γ-C11-d4TTP **7x**; lane 7 (T, C_{18}-d4T): γ-C18-d4TTP **7z**; lane 8 (d4TTP): d4TTP.

Assay condition: 60 mM Tris-HCl (pH 8), 5 mM MgOAc, 1.0 mM dithiothreitol, 0.01% (w/m) of bovine serum albumin, dNTPs 250 µM, DNA polymerase α 4 U, hybrid 0.20 µM in a reaction volume of 25 µL, incubated 37 °C for 5 min without dNTPs, then incubated 60 min at 37°C, 80 °C for 3 min; 50 mA, 45w for 3 h.

Author Contributions

CM headed the project; CZ performed the chemical synthesis and did the biochemical assays, SW performed the polymerase α and γ assays. DS and JB carried out the antiviral testing of the synthesized compounds. All authors were involved in the preparation of the manuscript.
